# Discovery of unguisin J, a new cyclic peptide from *Aspergillus heteromorphus* CBS 117.55, and phylogeny-based bioinformatic analysis of UngA NRPS domains

**DOI:** 10.3762/bjoc.20.32

**Published:** 2024-02-19

**Authors:** Sharmila Neupane, Marcelo Rodrigues de Amorim, Elizabeth Skellam

**Affiliations:** 1 Department of Chemistry, University of North Texas, 1155 Union Circle, Denton, TX 76203, USA; 2 BioDiscovery Institute, University of North Texas, 1155 Union Circle, Denton, TX 76203, USA; 3 Instituto de Química de São Carlos, Universidade de São Paulo, CP 780, CEP 13560-970, São Carlos, SP, Brazil

**Keywords:** adenylation domain, condensation domain, fungal non-ribosomal peptide synthetase, heptapeptide, unguisin biosynthesis

## Abstract

Several under-explored *Aspergillus* sp. produce intriguing heptapeptides containing a γ-aminobutyric acid (GABA) residue with as yet unknown biological functions. In this study, a new GABA-containing heptapeptide – unguisin J (**1**) – along with known unguisin B (**2**) were isolated from a solid culture of *Aspergillus heteromorphus* CBS 117.55. The structure of compound **1** was elucidated by extensive 1D and 2D NMR spectroscopic analysis including HSQC, HMBC, COSY, and 2D NOESY as well as HRESIMS. The stereochemistry of **1** and **2** was determined by Marfey’s method. A biosynthetic gene cluster (BGC) encoding unguisins B and J was compared to characterized BGCs in other *Aspergillus* sp. Since the unguisin family of heptapetides incorporate different amino acid residues at different positions of the peptide, the A and C domains of the UngA NRPS were analyzed in an attempt to understand the lack of substrate specificity observed.

## Introduction

**Figure 1:**
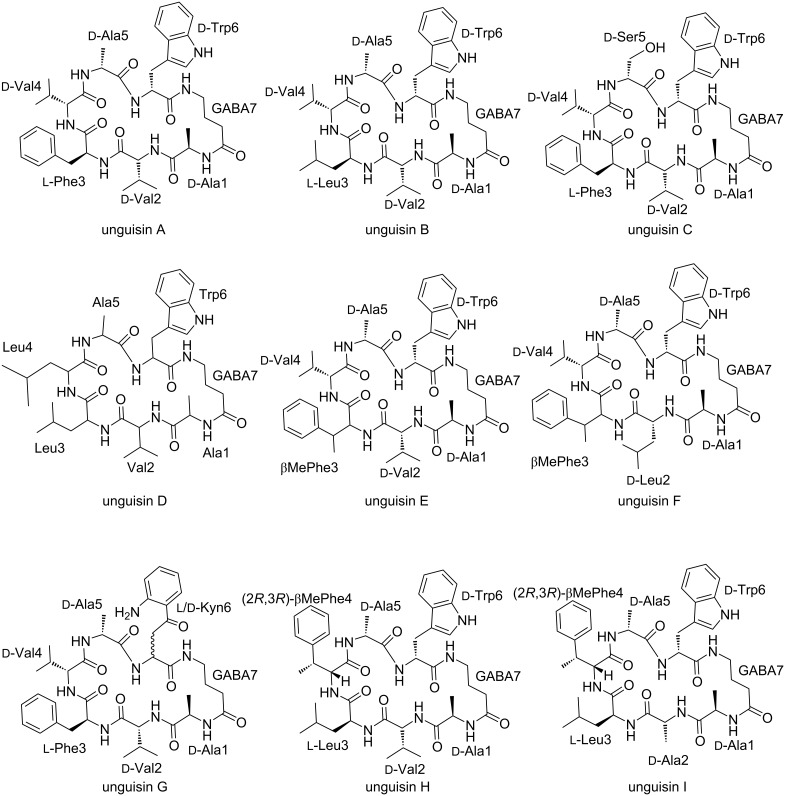
Structures of unguisins.

Unguisins are a small family of fungal cyclic heptapeptides isolated predominantly from *Aspergillus* sp. [1–8]. Distinctive features of these cyclic peptides include the non-proteinogenic amino acid γ-aminobutyric acid (GABA) and the incorporation of up to five ᴅ-amino acids (Figure 1) [1–8]. The amino acids at positions 1 (ᴅ-Ala) and 7 (GABA) are conserved but there is considerable variability at positions 2–6, including the incorporation of additional non-proteinogenic amino acids β-methylphenylalanine (βMePhe) and kynurenine (Kyn) [3–4]. So far, no significant biological activities have been reported for these small peptides [3–4,9], however, unguisin A has been shown to bind a series of anions [10].

Recently the biosynthesis of unguisins A and B from *Aspergillus violaceofuscus* CBS 115571 was reported [5]. A seven module non-ribosomal peptide synthetase (NRPS; UngA) was heterologously expressed in *Aspergillus oryzae* NSAR1 yielding both unguisins A and B, which differ by the incorporation of ᴅ-Phe and ᴅ-Leu at position 3, respectively. The highly conserved ᴅ-Ala at position 1 was shown to be synthesized from ʟ-alanine via the PLP-dependent alanine racemase UngC [5]. A hydrolase/peptidase (UngD) was also discovered that linearized the cyclic unguisins to linear peptides during in vitro investigations, although the linear peptides were not detected from the fungal cultures.

NRPS enzymes are large multifunctional enzymes that often synthesize very important bioactive molecules [11–12]. These enzymes consist of several catalytic domains organized into modules. Typically, a module possesses an adenylation (A) domain for selecting and activating amino- or keto acids, a thiolation (T) domain for shuttling intermediates between catalytic domains, and a condensation (C) domain that catalyzes amide or ester bond formation. Additional common domains include epimerization (E) domains for converting naturally occurring ʟ-amino acids to ᴅ-amino acids, methyltransferase (MT) domains that typically methylate specific N atoms, and terminal condensation (C_T_) domains which cyclize the growing peptide chain and facilitate release from the NRPS. Of the fungal NRPS studied to date, many appear to have some tolerance for the range of amino acids incorporated by the A domains and the C domain has been highlighted as a gatekeeper [13].

Here, we describe the isolation of unguisin B, and a new congener named unguisin J, from *Aspergillus heteromorphus* CBS 117.55. We also perform bioinformatic analysis of the A and C domains of the UngA NRPS enzymes involved in their biosynthesis to try and rationalize the relaxed substrate specificity observed in this family of heptapeptides.

## Results and Discussion

**Figure 2:**
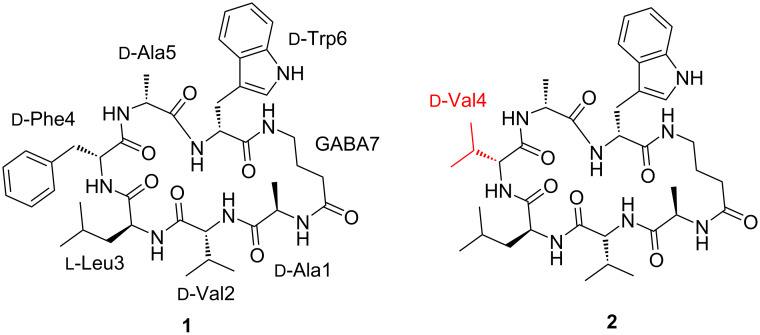
Chemical structures of unguisin J (**1**) and unguisin B (**2**).

The cultivation of *A. heteromorphus* CBS 117.55 on rice solid medium yielded an organic-soluble extract, which was subjected to fractionation using preparative HPLC-PDA-ELSD and purification by semipreparative HPLC-PDA; this led to the isolation of a new cyclic peptide **1**, along with unguisin B (**2**, Figure 2). The structure of the new compound **1** was elucidated by 1D and 2D NMR and HRESIMS/MS. Unguisin B was identified by the ^1^H and ^13^C NMR data with the reported data [1,5].

Compound **1** was obtained as a white amorphous solid optically active, with 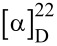 +23.4 (*c* 0.1, MeOH). Its molecular formula was established as C_41_H_56_N_8_O_7_ by HRMS ([M + H]^+^ at *m*/*z* 773.4338, calculated for C_41_H_57_N_8_O_7_^+^, *m*/*z* 773.4345, Δ 0.9 ppm; [M + Na]^+^ at *m/z* 795.4162, calculated for C_41_H_56_N_8_O_7_Na^+^, *m*/*z* 795.4164, Δ 0.3 ppm) and NMR data analysis, corresponding to eighteen indices of hydrogen deficiency. Its UV spectrum exhibited absorption maxima at λ_max_ 219 and 279 nm.

**Figure 3:**
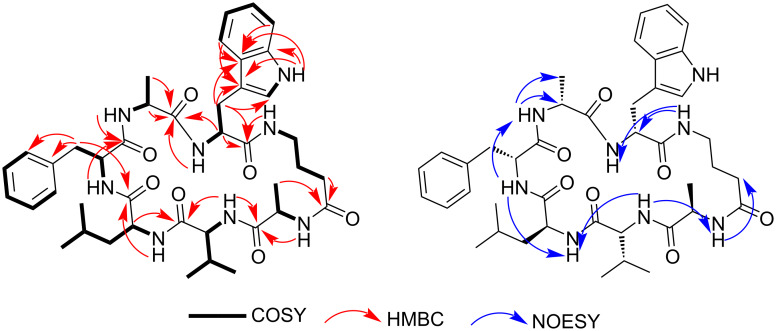
Key *g*HMBC and *g*COSY correlations, and NOESY interactions of **1**.

The ^1^H and ^13^C NMR spectra of **1** revealed the presence of seven amide NH signals between δ_H_ 7.43 and 8.44 ppm supported by the amide carbonyl signals at δ_C_ 173.1, 172.6, 172.6, 172.1, 172.1, 171.1 and 171.0 ppm (Table 1). An additional NH signal at δ_H_ 10.82 ppm and four aromatic signals at δ_H_ 7.50, 7.33, 7.07 and 6.97 ppm, exhibiting key ^1^H,^1^H-COSY and HMBC correlations, suggested a tryptophan aromatic amino acid portion (Figure 3). The other six amino acid residues were assigned based on 2D NMR spectra (^1^H-^1^H COSY, HSQC and HMBC) as Ala (2 equiv), Phe (1 equiv), Leu (1 equiv), Val (1 equiv), and γ-aminobutyric acid (GABA) (1 equiv). In addition to the COSY and HMBC correlations, the NOESY experiment showed important interactions between the NH signals corroborating with the peptide sequence defined to be Ala-1, Val-2, Leu-3, Phe-4, Ala-5, Trp-6, and GABA-7 (Figure 3).

**Table 1:** 1D and 2D NMR data for **1** (^1^H: 500 MHz, ^13^C: 125 MHz; DMSO-*d*_6_).

**1**						

residue/position		δ_C_	δ_H_ (mult., *J* in Hz)	NOESY	HMBC	COSY

alanine	NH	–	8.35 (d, 4.2)	2.14, 1.98, 7.42	172.1, 50.2	3.83
	Cα	50.2	3.83 (m)		17.0	8.35, 1.13
	Cβ	17.0	1.13 (d, 6.9)		172.6, 50.2	3.83
	C=O	172.1	–	–	–	–
valine	NH	–	7.43 (d 8.9)	8.35, 8.14	172.1	3.98
	Cα	58.4	3.98 (t 8.7)		30.0, 18.2, 172.1	7.43, 1.98
	Cβ	30.0	1.98 (m)		19.0, 58.4, 172.6	3.98, 0.68
	Cγ	18.2	0.68 (d, 6.7)		58.4, 30.0, 19.0	1.98, 0.78
	Cγ	19.0	0.78 (d, 6.7)		58.4, 30.0, 18.2	0.68
	C=O	172.1	–	–	–	–
leucine	NH	–	8.14 (d, 7.6)	7.43	171.1, 51.8	3.88
	Cα	51.8	3.88 (m)		39.4, 172.1	8.14, 1.36
	Cβ	39.4	1.36 (m)		23.6, 23.3, 20.8	3.88, 0.98
	Cγ	23.6	0.98 (m)		–	1.36, 0.56
	Cδ	20.8	0.56 (d, 6.6)		39.4, 23.6, 23.3	0.98, 0.69
	Cδ	23.3	0.69 (d, 6.6)		39.4, 23.6, 20.8	0.98, 0.56
	C=O	171.1	–	–	–	–
phenylalanine	NH	–	8.44 (d, 4.7)	8.14, 7.98	173.1	4.15
	Cα	55.8	4.15 (m)		171.1	8.44, 2.93, 3.01
	Cβ	36.1	2.93 (m), 3.01 (m)		55.8, 129.2, 137.2	4.15
	C-1	137.2	–	–	–	–
	C-2, C-6	129.2	7.16 (dd, 7.5, 1.3)		126.4	7.24
	C-3, C-5	128.3	7.24 (d, 7.5)		137.2, 128.3	7.16, 7.19
	C-4	126.4	7.19 (dd, 7.5, 1.3)		129.2	7.24
	C=O	173.1	–	–	–	–
alanine	NH	–	7.98 (d, 5.1)	4.06, 1.12	172.6	4.06
	Cα	48.7	4.06 (m)		173.1, 17.5	7.98, 4.06
	Cβ	17.5	1.12 (d, 6.9)		173.1, 48.7	4.06
	C=O	172.6	–	–	–	–
tryptophan	NH	–	7.86 (d, 6.9)	7.61	172.6	4.01
	Cα	55.3	4.01 (m)		172.6, 171.1, 110.7, 25.0	7.86, 3.22
	Cβ	25.0	3.22 (m)		127.2, 123.7, 110.7, 55.3	4.01
	NH	–	10.82 (d, 1.7)		136.4, 127.2, 123.7, 110.7	7.06
	C-2	123.7	7.06 (br s)		110.7, 127.2, 136.4	10.82
	C-3	110.7	–	–	–	–
	C3a	127.2	–	–	–	–
	C-4	118.3	7.50 (br d, 8.0)		136.4, 121.1, 127.2w, 110.7w	6.97
	C-5	118.4	6.97 (dt, 8.0, 0.9)		111.5, 127.2	7.50, 6.97
	C-6	121.1	7.07 (dt, 8.0, 1.0)		136.4, 118.4, 118.3	6.97, 7.33
	C-7	111.5	7.33 (dt, 8.0, 0.9)		127.2, 118.4, 118.3	7.07
	C-7a	136.4	–	–	–	–
	C=O	171.0	–	–	–	–
GABA	NH	–	7.61 (dd, 5.4, 4.3)	7.86, 4.01	171.0	3.07, 2.99
	Cα	38.8	3.07 (m), 2.99 (m)		25.6	7.61, 1.69, 1.60
	Cβ	25.6	1.69 (m), 1.60 (m)		38.8w	3.07, 2.99, 1.69, 1.60
	Cγ	32.9	2.14 (m), 1.98 (m)		25.6, 38.8	1.69, 1.60
	C=O	172.6	–	–	–	

w. weak.

Analysis of the NMR data of **1** allowed identifying characteristic ^1^H and ^13^C signals very similar to those of unguisin B (**2**) [1,5], the difference being the replacement of the Phe-4 in **1** by Val-4 in **2**. This assignment was confirmed by observation of HMBC correlations from δ_H_ 7.98 (NH) and δ_H_ 8.44 (NH) to C=O (δ_C_ 173.1) and from δ_H_ 2.93 and 3.01 (H_2_-β) to C=O (δ_C_ 171.1) (Figure 3), together with key NOESY interactions between the NH signals at δ_H_ 7.98↔8.44↔8.14.

The absolute configuration of **1** was assigned by Marfey’s method [14]. Comparison of the retention time by LC–MS between the derivatized **1** as well as the authentic amino acid samples determined the structure of **1** as cyclo(ᴅ-alanine-ᴅ-valine-ʟ-leucine-ᴅ-phenylalanine-ᴅ-alanine-ᴅ-tryptophan-GABA). Compound **1** was named as unguisin J.

A second peptide was isolated from the same culture of *A. heteromorphus* CBS 117.55. Compound **2** was obtained as an amorphous white powder, 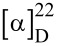 +37 (*c* 0.1, EtOH) [lit **+**40 (*c* 1.0, EtOH)] [5]; for the ^1^H and ^13^C NMR spectroscopic data, see Table S2 in Supporting Information File 1. By comparison with literature data this compound was identified as unguisin B (**2**) [1,5], further corroborating the identification of the new unguisin J (**1**).

To the best of our knowledge these are the first metabolites reported from *A. heteromorphus* CBS 117.55.

**Figure 4:**
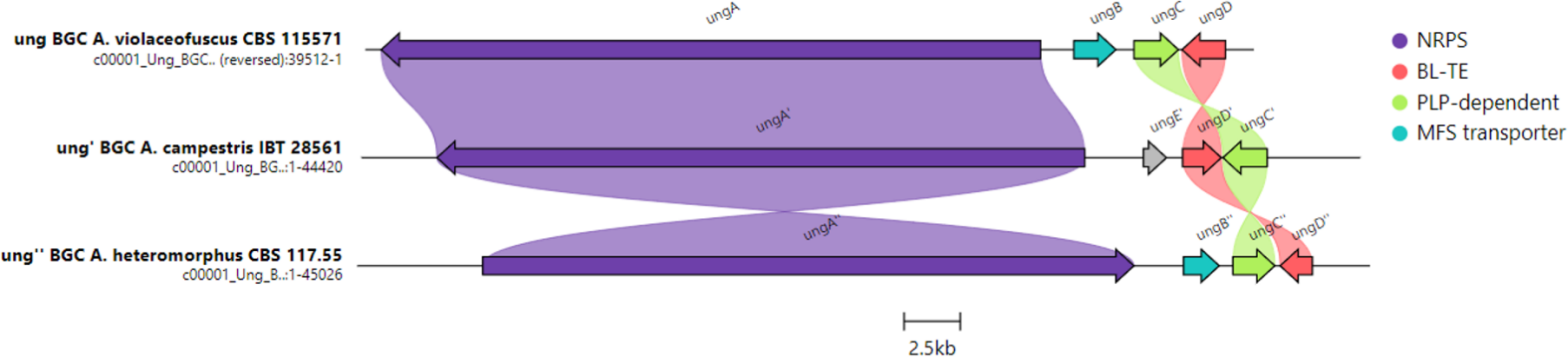
Clinker analysis of identified unguisin-encoding BGCs. UngE’ is a methyltransferase that methylates phenylalanine and appears only in the *A. campestris* BGC.

**Scheme 1:**
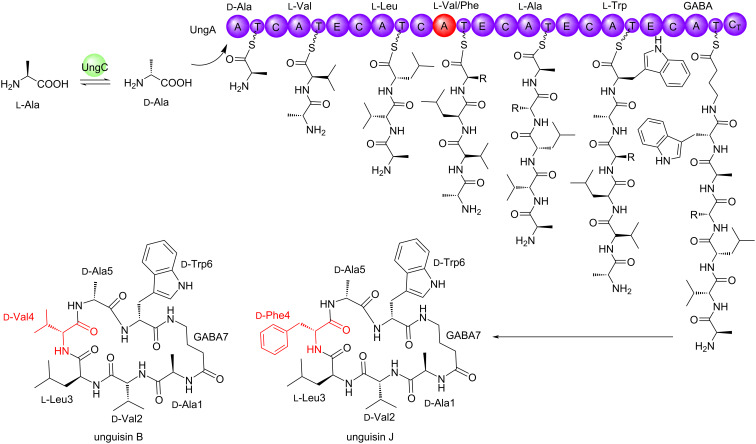
Proposed biosynthesis of unguisins B and J in *A. heteromorphus* CBS 117.55.

The co-isolation of unguisins B and J indicates that module 4 of the NRPS is able to accept two different amino substrates and so may possess subtle differences to UngA from *A. violaceofuscus* CBS 115571 which has relaxed substrate specificity in module 3. We performed genome mining of the publicly available *A. heteromorphus* CBS 117.55 (accession number MSFL00000000.1) [15] using fungiSMASH and identified a four gene BGC encoding a seven module NRPS, an alanine-racemase, a hydrolase, and a transporter. We named this BGC *ung’’* to distinguish it from the *ung* BGC present in *A. violaceofuscus* and the *ung’* BGC in *A. campestris* IBT 28561 which encodes unguisins H and I [5]. Clinker analysis with the *ung* BGCs from *A. violaceofuscus* CBS 115571 and *A. campestris* IBT 28561 indicated a high level of homology (Figure 4). The biosynthesis of unguisins B and J therefore is proposed to arise from this single BGC, similar to the biosynthesis of unguisins A and B in *A. violaceofuscus* CBS 115571 (Scheme 1).

**Figure 5:**
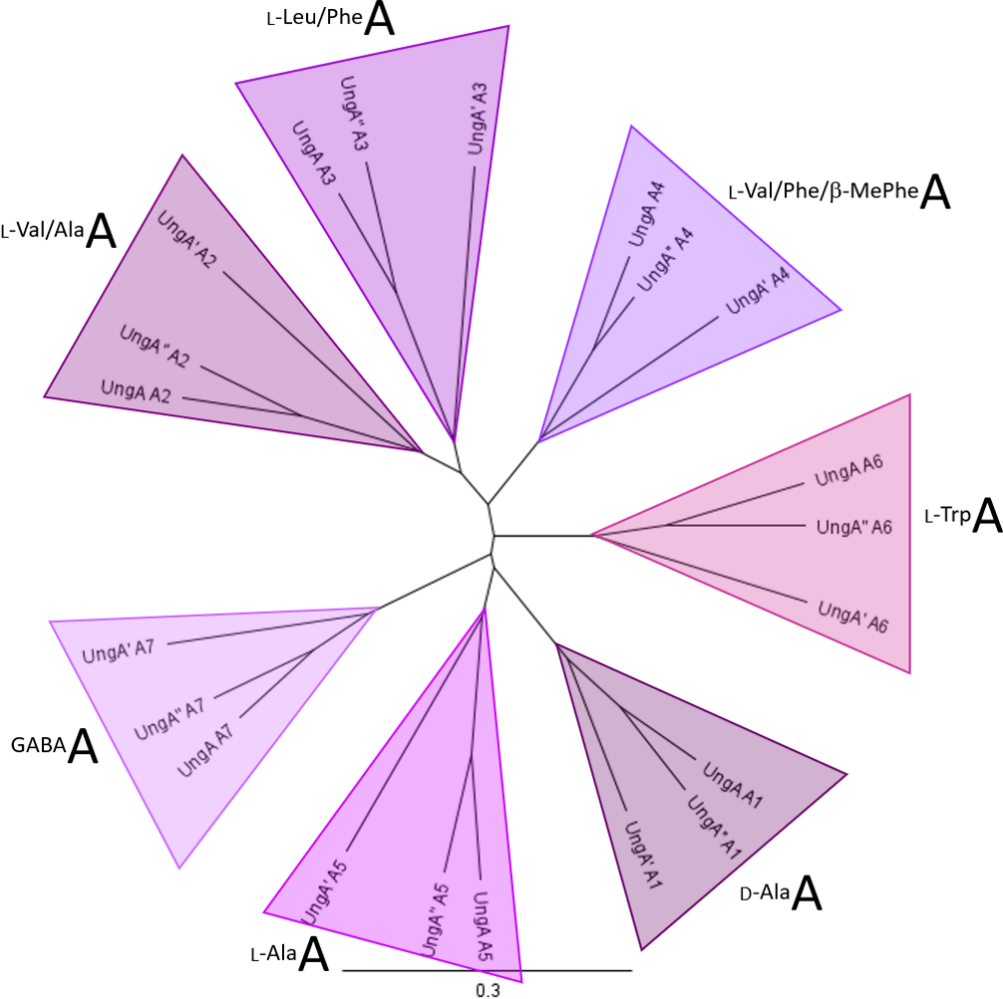
Phylogenetic analysis of A domains extracted from UngA NRPS. The substrate of the A domain is indicated for each clade.

**Figure 6:**
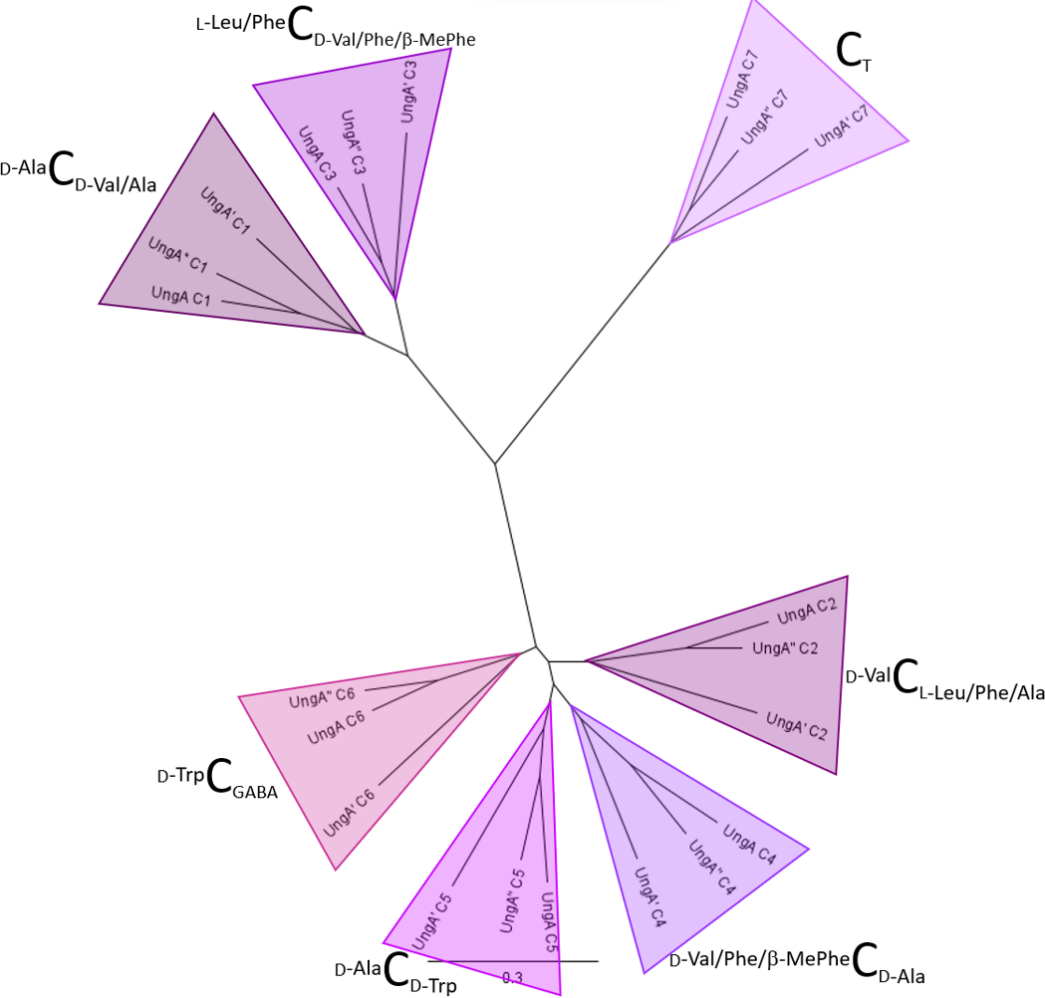
Phylogenetic analysis of C domains extracted from UngA NRPS. The substrates condensed by each C domain is indicated.

Within the unguisin family, there is variability in the amino acids incorporated at positions 2–6 (Figure 1), however, there are usually only one or two residue differences between molecules that are co-isolated from each source, e.g., A and B from *A. violaceofuscus* CBS 115571 [5]; A, B, and C from *Emericella unguis* [1]; A, E, F and G from *Aspergillus candidus* NF2412 [4]; H and I from *A. campestris* IBT 28561 [5]; and B and J from *A. heteromorphus* CBS 117.55 (Figure 1). This implies that only one or two modules per NRPS possesses a noticeable level of relaxed substrate specificity. To explore this observation, the A and C domains were identified in UngA, UngA’ and UngA’’ and phylogenetic analysis of the A and C domains was performed (Figure 5 and Figure 6).

The A domains do not clade according to substrate specificity – instead they clade according to which module they were extracted from. The A domains from modules 2, 3, and 4, which have relaxed substrate specificity, do appear to have evolved differently than A domains from modules 1, 5, 6, and 7 (Figure 5). Perhaps unsurprisingly the domains from UngA and UngA’’ which both synthesize unguisin B, were more closely related than those from UngA’ despite differences in substrate specificity in modules 3 and 4. Previously Matsuda et al. had compared the putative non-ribosomal codes for the UngA and UngA’ A domains and also observed that conventional approaches are inadequate to understand or predict the specificity of fungal A domains [5].

The clades formed by the C domains showed higher divergence than the A domains with the C_T_ domains forming their own branch and C domain from modules 1 and 3 clearly distinct to those from modules 2, 4, 5, and 6 (Figure 6). This separation of the non-terminal C domains could be due to modules 1 and 3 lacking an E domain. Again, the domains from UngA and UngA’’ were more closely related than those from UngA’ regardless of which two amino acids were condensed.

## Conclusion

In this study unguisins B and J were isolated from *A. heteromorphus* CBS 117.55 which has not been extensively investigated for secondary metabolite production. A BGC encoding the unguisins was identified by genome mining with high homology to *ung* BGCs from other *Aspergillus* sp. Phylogenetic analysis of the A and C domains extracted from the UngA NRPS indicates that domains within modules are more closely related – even when substrate specificity differs – than domains within other modules that accept the same substrates.

## Experimental

### General experimental procedures

*A. heteromorphus* CBS 117.55 (also known as A. heteromorphus NRRL 4747) was purchased from the ARS Culture Collection. Rice solid medium was purchased from RiceSelect Organic Texmati. All solvents used for conducting LC analysis were purchased from Fisher Scientific. DMSO-*d*_6_ NMR solvent was purchased from Sigma-Aldrich. *N*^α^-(5-Fluoro-2,4-dinitrophenyl)-ᴅ-leucinamide was purchased from TCI Chemicals. Authentic amino acid samples were purchased from Thermo Scientific. Sodium bicarbonate purchased from Fisher Chemical.

1D and 2D NMR experiments were recorded on a Varian INOVA 500 instrument (^1^H: 500 MHz; ^13^C: 125 MHz). The chemical shifts (δ) were expressed in ppm and recorded with reference to solvent signals (^1^H NMR: DMSO-*d*_6_ 2.50 ppm; ^13^C NMR: DMSO-*d*_6_ 39.5 ppm). Optical rotation was measured on a Jasco P-2000 polarimeter with a path length of 100 mm. Analytical HPLC-PDA-MS system was a Shimadzu instrument (LC2030C 3D Plus Prominence) coupled to a Shimadzu LCMS-2020 mass spectrometer. Analyses were performed using a Phenomenex Kinetex RP_18_ column (100 mm × 4.6 mm i.d., 2.6 μm) along with the Security Guard RP_18_ protective guard column (4.6 mm i.d.) and eluting with H_2_O + 0.1% formic acid and MeCN + 0.1% formic acid using a gradient from 90:10 to 10:90 of H_2_O/MeCN over 15 min, maintaining in 10:90 H_2_O/MeCN for 3 min, from 10:90 to 90:10 in 1 min, and maintaining at 90:10 for 1 min, using a flow rate of 1.0 mL/min. The PDA detector scanned between λ = 190 and 700 nm. The MS was optimized using the following conditions: interface voltage 4.5 kV; interface temperature 350 °C; DL temperature 250 °C; heat block 200 °C; ESI mode, acquisition range 100 to 1000 Da; nebulizing gas 1.5 L min^−1^; drying gas flow 15 L min^−1^. The fractionation of the sample was performed on a Shimadzu LC-20AP preparative liquid chromatograph (SCL-40 System Controler Deliver and LH-40 Liquid Handler) coupled to a Shimadzu SPD-M40 Photo Diode Array Detector (PDA) system using a RP-18 column (Phenomenex, Kinetex 250 × 30 mm i.d., 5 µm, flow rate of 18.0 mL min^−1^). The purification of compounds was performed on a Shimadzu LC-20AD liquid chromatography (CBM-20A Communication Bus Module, CTO-20A column oven, DGU-20A Degassing Unit and SIL-20A AutoSampler) coupled to a Shimadzu SPD-20A UV–vis Detector system using a RP-18 column (Shimadzu, Premier 250 × 10 mm i.d., 5 µm, flow rate of 3.0 mL min^−1^). High-resolution mass spectra were recorded on an ABSciex TripleTOF 6600+ mass spectrometer. Direct infusion of compounds **1** and **2** through the high-resolution mass spectrometry (HRMS) was performed using a flow rate of 10 μL min^−1^ which the samples were diluted at 10 ppm with a solution of MeCN/H_2_O (50:50; v/v) containing 0.1% formic acid. The parameters such as declustering and entrance potentials remained constant for MS and MS/MS were set up at 150 V and 10 V, respectively. Collision energy for MS and MS^2^ scan surveys was 10 V and 45 V, respectively, with a collision energy spread of 12 V for MS^2^ scan survey. Precursor ion was impacted with three different collision energies (33, 45, 57 V), and the resulting MS^2^ spectra were combined into one final MS^2^ spectrum. The mass spectra were acquired using Turbo Spray Ionization set to 5.5 kV in positive ion mode with an accumulation time of 100 ms. The mass ranges for MS and MS^2^ scan surveys were 500–800 amu and 30–800 amu, respectively. The curtain gas (nitrogen), nebulizing and heating gas were fixed at 25 psi, 20 psi and 15 psi, respectively. The temperature of the source was 25 °C. MS spectra were acquired and processed using Analyst TF 1.8.1 software.

### Fungal growth and extraction

*A. heteromorphus* CBS 117.55 was cultivated in 2 Erlenmeyer flasks (500 mL), each containing 90 g of rice and 150 mL of H_2_O [16]. The medium was autoclaved at 121 °C for 20 min. After sterilization, the medium was inoculated with the spore solution of *A*. *heteromorphus* (1 mL) and incubated in static mode at 25 °C for 21 days. The following day, the cultured mass in the flasks was ground and extracted with ethyl acetate (EtOAc, 3 × 100 mL). The EtOAc fraction was dried using a rotary evaporator and then dissolved in CH_3_CN for defatting with hexane by partitioning. The CH_3_CN fraction was evaporated, yielding 0.601 g of soluble-organic extract.

### Fractionation and isolation of unguisins J and B

The soluble-organic extract was fractionated by preparative HPLC-PDA using Kinetex RP18 column (250 mm × 30 mm i.d., 5 μm) and UV detector at λ_max_ = 254 nm. The mobile phase consisted of H_2_O + 0.05% formic acid (eluent A) and MeCN + 0.05% formic acid (eluent B), which was eluted of 20–100% of B with flow rate of 18 mL min^−1^, yielding 20 fractions.

Fractions Fr13 and Fr15 were subjected to a purification by semipreparative HPLC-UV using a Premier RP_18_ column (250 mm × 10 mm i.d., 5 μm) and UV detector at λ_max_ = 210 nm. The gradient elution consisted from 65:35 to 35:65 of H_2_O/MeCN over 20 min, using a flow rate of 3.0 mL min^−1^. Fractions Fr13 and Fr15 resulted in the isolation of **1** (11.5 mg) and **2** (14.1 mg), respectively.

**Unguisin J (1).** Amorphous white powder, 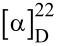 +23.4 (*c* 0.1, MeOH); UV (photodiode array, MeCN/H_2_O) λ_max_ = 219 and 279 nm; HRESIMS *m*/*z*: [M + H]^+^ calcd for C_41_H_57_N_8_O_7_^+^, 773.4345; found, 773.4338, [M + Na]^+^ calcd for C_41_H_56_N_8_O_7_Na^+^, 795.4164; found, 795.4162. For the ^1^H and ^13^C NMR spectroscopic data, see Table 1.

### Bioinformatics

The *A. heteromorphus* CBS 117.55 genome was initially screened using fungiSMASH to identify scaffolds/contigs encoding secondary metabolites. Scaffold MSFL01000005.1 was further investigated using FGENESH [17] to further refine gene boundaries, introns, and resulting protein sequence (Table S1, Supporting Information File 1). Comparative genomics/Clinker analysis was performed using Cagecat [18–19]. A and C domains were identified via Scan Prosite [20] and fungiSMASH [21]. Sequence alignments (Figures S1 and S2, Supporting Information File 1) and phylogenetic trees were generated using Geneious^TM^. Functional domain motifs were visualized using Web Logo [22] and compared to literature motifs (Figures S3 and S4, Supporting Information File 1).

### Marfey’s method to determine the absolute configuration of **1** and **2**

Samples **1** and **2** (0.3 mg each) were hydrolyzed in 0.5 mL of 6 N HCl at 115 °C for 20 h. After cooling, the reaction mixture was evaporated under nitrogen gas flow. The residue was dissolved in 0.5 mL of H_2_O, and dried using speedvac to remove the residual HCl. The hydrolysates of **1** and **2** as well as authentic amino acid samples were treated with 200 μL of 1 N sodium bicarbonate solution and 100 μL of 1% Marfey’s reagent (*N*^α^-(5-fluoro-2,4-dinitrophenyl)-ᴅ-leucinamide) in acetone [14]. The samples were incubated for 1 h at 60 °C followed by neutralization with 100 μL of 2 N HCl. The HCl was removed by using N_2_ gas. Then, the samples were diluted by 2 mL of MeCN/H_2_O (1:1, v/v) solution, filtered with 0.22 μm filter and analyzed by LC–MS for comparison of the retention times.

## Supporting Information

File 1 Spectroscopic and spectrometric data of **1** and **2**. Bioinformatic data of the biosynthetic gene clusters.

## Data Availability

All data that supports the findings of this study is available in the published article and/or the supporting information to this article.
