# Supplementary material for: Discovery of unguisin J, a new cyclic peptide from Aspergillus heteromorphus CBS 117.55, and phylogeny-based bioinformatic analysis of UngA NRPS domains
Source: Beilstein J Org Chem. 2024 Feb 19;20:321–30. doi: 10.3762/bjoc.20.32 (PMC10896222; doi:10.3762/bjoc.20.32)
Supplement: File 1 — Spectroscopic and spectrometric data of 1 and 2. Bioinformatic data of the biosynthetic gene clusters. [file Beilstein_J_Org_Chem-20-321-s001.pdf]

BEILSTEIN JOURNAL OF ORGANIC CHEMISTRY

Supporting Information

for

Discovery of unguisin J, a new cyclic peptide from *Aspergillus heteromorphus* CBS 117.55, and phylogeny-based bioinformatic analysis of UngA NRPS domains

Sharmila Neupane, Marcelo Rodrigues de Amorim and Elizabeth Skellam

Beilstein J. Org. Chem. **2024,** *20,* 321–330. doi:10.3762/bjoc.20.32

Spectroscopic and spectrometric data of 1 and 2. Bioinformatic data of the biosynthetic gene clusters

License and Terms: This is a supporting information file under the terms of the Creative Commons Attribution License (https://creativecommons.org/licenses/by/4.0). Please note that the reuse, redistribution and reproduction in particular requires that the author(s) and source are credited and that individual graphics may be subject to special legal provisions.
The license is subject to the *Beilstein Journal of Organic Chemistry* terms and conditions: (https://www.beilstein-journals.org/bjoc/terms)

Table of Contents

List of supplementary tables:

Table 1S: DNA and protein sequences of ung" BGC mined from *A. heteromorphus* CBS 117.55. ....S3
Table 2S: ^{1}H and ^{13}C NMR data for **2** (^{1}H: 500MHz, ^{13}C:125MHz; DMSO-*d*6). S46
Table 3S: Summary of additional NRPS identified in the genome of *A. heteromorphus* CBS 117.55. Abbreviations as main text: C_T = cyclizing C domain; X = undefined region after CDS analysis; R = reductase. S47

List of supplementary figures:

Figure S1: A domain sequence alignment comparing UngA, UngA', and UngA''. S13
Figure S2: C domain sequence alignment comparing UngA, UngA', and UngA''.......S13
Figure S3: A domain AMP binding motif identified in extracted A domains. S13
Figure S4: C domain active site identified in extracted C domains. S14
Figure S5: ^{1}H NMR spectrum of **1** (500 MHz, DMSO-*d*6). S15
Figure S6: Expansion of the ^{1}H NMR spectrum of **1** (500 MHz, DMSO-*d*6). S16
Figure S7: Expansion of the ^{1}H NMR spectrum of **1** (500 MHz, DMSO-*d*6). S17
Figure S8: Expansion of the ^{1}H NMR spectrum of **1** (500 MHz, DMSO-*d*6). S18
Figure S9: ^{13}C NMR spectrum of **1** (125 MHz, DMSO-*d*6). S19
Figure S10: *g*HSQC spectrum of **1** (^{1}H:500 MHz; ^{13}C:125 MHz, DMSO-*d*6). S20
Figure S11: *g*HMBC spectrum of **1** (^{1}H:500 MHz; ^{13}C:125 MHz, DMSO-*d*6). S21
Figure S12: *g*COSY spectrum of **1** (500 MHz, DMSO-*d*6). S22
Figure S13: 2D-NOESY spectrum of **1** (500 MHz, DMSO-*d*6). S23
Figure S14: HRESIMS spectrum of **1** in positive ionization mode. S24
Figure S15: HRESIMS/MS spectra of **1** and fragmentation patterns in positive ionization mode. S25
Figure S16: UV spectrum (photodiode array, H_2O:MeCN) of **1**. S26
Figure S17: HPLC-PDA chromatogram of unguisin J (**1**). S27
Figure S18: ^{1}H NMR spectrum of **2** (500 MHz, DMSO-*d*6). S28
Figure S19: Expansion of the ^{1}H NMR spectrum of **2** (500 MHz, DMSO-*d*6).............S29
Figure S20: Expansion of the ^{1}H NMR spectrum of **2** (500 MHz, DMSO-*d*6).............S30
Figure S21: Expansion of the ^{1}H NMR spectrum of **2** (500 MHz, DMSO-*d*6).............S31
Figure S22: ^{13}C NMR spectrum of **2** (125 MHz, DMSO-*d*6). S32
Figure S23: *g*HSQC NMR spectrum of **2** (^{1}H:500 MHz; ^{13}C:125 MHz, DMSO-*d*6). S33
Figure S24: Expansion of the *g*HSQC NMR spectrum of **2** (^{1}H:500 MHz; ^{13}C:125 MHz, DMSO-*d*6). S34
Figure S25: UV spectrum (photodiode array, H_2O:MeCN) of **2**. S35
Figure S26: HRESIMS spectrum of **2** in positive ionization mode. S36

Figure S27: HPLC-PDA chromatogram of unguisin B (**2**)...S37
Figure S28: HPLC-PDA chromatogram of soluble-organic extract from rice culture of *A. heteromorphus*. Peak labels represent unguisin J (**1**) and Unguisin B (**2**)..................S38
Figure S29: LC-MS analysis of *N*$^{\alpha}$-(5-fluoro-2,4-dinitrophenyl)-D-leucinamide derivatives of the hydrolysates of **1** and **2** as well as the alanine amino acid standards…...S39
Figure S30: LC-MS analysis of *N*$^{\alpha}$-(5-fluoro-2,4-dinitrophenyl)-D-leucinamide derivatives of the hydrolysates of **1** and **2** as well as the valine amino acid standards…… S40
Figure S31: LC-MS analysis of *N*$^{\alpha}$-(5-fluoro-2,4-dinitrophenyl)-D-leucinamide derivatives of the hydrolysates of **1** and **2** as well as the leucine amino acid standards…...S41
Figure S32: LC-MS analysis of *N*$^{\alpha}$-(5-fluoro-2,4-dinitrophenyl)-D-leucinamide derivative of the hydrolysate of **1** as well as the phenylalanine amino acid standards……........S42
Figure S33: LC-MS analysis of *N*$^{\alpha}$-(5-fluoro-2,4-dinitrophenyl)-D-leucinamide derivatives of the hydrolysates of **1** and **2** as well as the D-tryptophan standard. S43
Figure S34: Colonies of *Aspergillus heteromorphous* CBS 117.55 with 7 days of growth at 25 ℃. Colonies on DPY agar plate verse (**A**) and obverse (**B**)...............................S44
Figure S35: Colonies of *Aspergillus heteromorphous* CBS 117.55 with 21 days of growth at 25 ℃ on solid rice in Erlenmeyer flasks. S45

Table S1: DNA and protein sequences of ung" BGC mined from *A. heteromorphus* CBS 117.55.

Gene	Putative DNA sequence	Predicted protein sequence
ungA''	ATGCCGACAGAGAATGGGGAGGTCCAGCACCGGATCGACGGCCTTCAGGATCAGGATACTGTGGAGATATTGCTGGTTACCTTGGAATC	MPTENGEVQHRIDGLQDQDTVEILLVTLES
	ATGGGCCCTGCAGGCTGTGGAGGAGTTCAAAGCCAGCAAGGGCATGCTCGAGATAGGCAGCGTGAGCGACAGATCGAACGATGCCCG	WALQAVEEFKASKGMLEIGSVSDRSNDARK
	TAAGGTGAAGATTGAGCTAGATGGGGGAACTGAGAATATATTGGCGCAATTGCGAGGGGAATGGGTGCGCCAGCTGGTGCACCAGCAC	VKIELDGGTENILAQLRGEWVRQLVHQHHE
	CACGAAGCAGCCAGTAATTTTGCGGCGTCCATAATTTTGGATGGCAGCCCAGAGAGGCTGCTGGTCTTGCGGTTTATGGAAAGGAGGG	AASNFAASIILDGSPERLLVLRFMERREAWVS
	AAGCCTGGGTGTCGGAGGAGGCGCCTAAGAGCTGGTGCCAGGAAAGGAGGTGTGCACCGTTCCTAGAGATGCATTGCCAGGCTCGCC	EEAPKSWCQERRCAPFLEMHCQARPEELQF
	CAGAAGAGCTGCAATTCCATATCGTCTCGCCTCCTCCTGTTGCGGCGCAAGCATTTGTGCAGCACCTGGATTTGCGGATCCGACAGTCCA	HIVSPPPVAAQAFVQHLDLRIRQSISNRSLAD
	TTTCCAATCGCAGTCTCGCAGACGTGCCGGTAGAGACTGTGCCTGCCGCGTCGGACAGCGAGCTCAGGGCCATCTGGGGCTGGAATGG	VPVETVPAASDSELRAIWGWNGTLPEGVQR
	CACGCTTCCTGAGGGAGTGCAGAGATGCGTTCACGATATCATCGCCCAGCAGGCGCGACTGCGGCCACACGCGCCAGCAGTCTCTGCCT	CVHDIIAQQARLRPHAPAVSAWDGELDYQQ
	GGGACGGCGAGCTGGACTATCAGCAACTGGATCGGATCTCCACTCGATTGGCCCATTACCTGGTGCAGCTGGGTGCTGGCCCAGATGAT	LDRISTRLAHYLVQLGAGPDDIIPLCFEKSKW
	ATCATCCCCCTCTGCTTCGAGAAGTCCAAGTGGATGATGGTGGCCATTCTGGCCGTGATGAAGTCAGGGGCTGTCATCGCTGCCCTGGAC	MMVAILAVMKSGAVIAALDPTQPEDRLRSIV
	CCGACACAGCCCGAGGATCGCCTGCGAAGCATTGTGAAGCAGCTGCAGCCACGCTGGATTCTGACATCGCCGGCCCAGATCGAAGTGG	KQLQPRWILTSPAQIEVAARLEISNVIALDEGR
	CCGCACGCCTGGAGATCAGCAATGTCATCGCTCTCGACGAAGGCCGCCTACGGCAACTGCCAGACTCCGAAGGAAGACATCTCCCATGC	LRQLPDSEGRHLPCVDPSRNLYIVFTSGSTGT
	GTTGATCCCTCCCGTAACCTGTACATTGTTTTCACGTCGGGCAGCACTGGAACGCCCAAGGGAGTCATGATCAATCACACCAACTTTAGC	PKGVMINHTNFSSAIAYQHEALGMDNTARV
	AGCGCCATCGCCTACCAGCATGAGGCATTGGGGATGGATAACACCGCGCGAGTGTTCGACTTCGCTTCATACGCCTTTGATCTGGCCTGG	FDFASYAFDLAWGNIIHTLAAGGCLCIPSESE
	GGGAACATCATCCATACCCTCGCGGCGGGGGGGTGTCTATGCATCCCCAGTGAAAGTGAGCGCCGTGGAAACATCGCCGAGGCAATCC	RRGNIAEAIRRLGVNHLQLTPSVARLIDPRDIP
	GCAGACTTGGGGTGAACCATCTCCAGCTGACCCCCAGTGTTGCTCGCCTCATCGATCCGCGAGATATCCCCGCAGTGCGATGGATCCTCC	AVRWILLIGEPMTQADVAQWTPYCKLINSYG
	TGATCGGAGAGCCAATGACCCAGGCCGATGTTGCTCAATGGACGCCATACTGCAAACTGATCAATTCCTACGGCCCAGCGGAATGCACG	PAECTVAVTFQTIPHGRPWDSSMGKGVACS
	GTGGCGGTGACCTTCCAGACAATCCCCCACGGCCGACCTTGGGACTCGAGTATGGGGAAAGGCGTCGCGTGCAGTACCTGGATCGTTG	TWIVDEEHGETLVPLGHTGELWLEGPLVGQ
	ACGAGGAGCATGGGGAGACACTGGTCCCCCTTGGCCACACAGGCGAGTTGTGGCTGGAAGGACCGCTGGTCGGCCAGGGATATCTAG	GYLGDLEKSAASFIDSPAWLTRGIPGVVPGRR
	GAGACCTGGAGAAGAGCGCGGCGAGTTTCATCGATAGCCCTGCCTGGCTGACCCGGGGAATACCAGGGGTCGTCCCCGGCCGGCGAG	GRLYKTRDLVRYNPDGSLVYVARKDTQIKIRG
	GCCGACTATATAAAACTAGAGATCTGGTGCGCTACAACCCGGACGGCTCGCTGGTTTACGTGGCTCGCAAGGATACGCAGATCAAGATCC	QRVELGDVEYHLKLALPDKIPSVAAEAITPRG
	GGGGCCAGCGGGTGGAACTGGGCGATGTGGAATATCACCTGAAGCTTGCACTGCCCGACAAGATTCCATCTGTTGCAGCCGAGGCCAT	SSSTILVAYLALGEEATGAAESTRESLASCLHG
	CACCCCGCGGGGTAGCAGCAGCACCATCCTCGTCGCATACCTGGCCCTCGGCGAAGAAGCAACTGGCGCCGCGGAGAGCACCCGGGA	VEEYLADRLPRYMVPSLYLAVPEIPMTATGKT
	GAGTCTAGCCAGTTGCCTGCATGGGGTGGAAGAGTACCTGGCCGACCGACTGCCTCGCTACATGGTGCCCAGCCTGTATCTTGCAGTCC	DRLRLREIGSSLTLDQLAALQPSRAVEIKAPET
	CCGAGATCCCCATGACTGCCACCGGCAAGACAGACCGGCTACGCCTGCGTGAGATCGGGTCTTCTTTAACCCTGGATCAGCTGGCAGCC	EMEHRLQQLWAATLNISPSSIGTGDSFLRIGG
	TTGCAGCCCTCGCGAGCAGTGGAGATCAAGGCTCCCGAGACCGAGATGGAGCACCGCCTTCAACAGCTCTGGGCGGCAACATTGAATA	ESMAAIRLVQLARKEGIILTVADIFNHPRLGD
	TCAGCCCGAGCAGCATAGGGACAGGCGACAGTTTCCTCCGAATCGGCGGAGAATCGATGGCAGCCATCCGCCTGGTGCAGCTCGCCCG	MAQEAQRERAQDVTTIPPFSLLRRGHDVRD
	GAAAGAGGGCATAATCCTGACAGTGGCCGATATTTTCAACCACCCGCGCCTCGGCGATATGGCGCAAGAAGCCCAACGAGAACGGGCC	ACALAAAECRVSANSIADLLPCTPLQEGLLAL
	CAGGACGTGACGACCATCCCTCCCTTCTCCCTGCTGCGTAGGGGCCACGACGTAAGAGATGCCTGCGCGTTGGCTGCAGCCGAATGCCG	TVKQAGEYVRQMVSELPVDVDLARFRAAW
	CGTTTCGGCAAACTCCATCGCCGACCTCCTGCCCTGCACCCCGCTGCAGGAAGGGCTGTTGGCCCTGACTGTCAAGCAGGCCGGGGAA	AQVIHEAAILRTRIVDLPNAGLLQVVVADQPE
	TATGTCCGCCAGATGGTATCAGAGCTTCCAGTGGATGTGGATCTGGCGCGCTTCCGCGCTGCCTGGGCCCAAGTGATTCATGAAGCGGC	WATGSDLGHFLESEKGKPMGLGTSLARFGLV
	GATTTTGCGAACGCGGATTGTGGACCTGCCCAACGCCGGGCTCCTGCAGGTCGTAGTCGCAGACCAGCCCGAATGGGCCACGGGGAG	SDQAQGKVLFVWTIHHALYDGWSLPAMLER
	CGATCTCGGTCATTTCTTGGAGTCAGAGAAAGGAAAGCCCATGGGACTGGGGACGTCGTTGGCGCGGTTTGGTCTGGTGTCGGATCAA	VEAIYAGGSCDMLPSFAGFVKYLADGTVEDA
	GCGCAAGGCAAGGTGCTTTTCGTCTGGACCATCCACCACGCCTTGTATGATGGCTGGTCGCTGCCGGCGATGCTGGAACGGGTGGAAG	QGYWQSQFNGIQAAVFPALPSPDYQPKCQD
	CCATTTATGCGGGTGGCAGCTGCGATATGCTCCCCTCGTTTGCGGGGTTTGTGAAATATCTGGCGGATGGTACAGTGGAAGATGCGCAG	LLQYHVASVSWPGNDITASTAVRTAWAIVAS
	GGTTACTGGCAATCCCAGTTCAATGGGATCCAGGCCGCAGTCTTCCCAGCCTTGCCCTCCCCAGACTACCAGCCCAAGTGTCAGGATCTG	RYTQSPDVIFGATVSGRQAPVPFVERMAGPT
	CTCCAATACCACGTCGCGAGTGTCTCCTGGCCCGGGAATGATATCACGGCCTCCACTGCAGTGCGAACAGCATGGGCAATTGTCGCAAG	IATVPVRVNVQGDATVASLMQSVQTQAVA
	CCGTTATACCCAGTCTCCGGATGTGATCTTTGGGGCCACAGTGTCCGGGCGACAGGCACCGGTCCCGTTCGTTGAACGCATGGCCGGTC	MIPYEQTGLNQIRRINSDADQATQFQSLLVV
	CAACCATTGCGACTGTTCCAGTCCGCGTGAATGTGCAGGGGGATGCCACGGTGGCCAGTTTGATGCAGAGCGTTCAGACACAGGCCGT	QPPSSKTSRRPDECLFRVDLDAGDEFRSINTY

	GGCTATGATTCCGTACGAGCAGACGGGATTGAACCAGATCCGGCGGATCAATTCGGATGCAGACCAGGCAACCCAGTTCCAGTCCCTGC	SLMLECHLESDGMRLRMSYDRLVIDAEQVK
	TGGTTGTACAGCCTCCAAGCTCGAAGACCTCTCGTCGGCCGGACGAGTGCTTGTTCCGAGTGGATCTGGATGCTGGTGACGAATTTCGC	RIARQFEGVLRQVCSEVNAQELVGTVSAASE
	AGTATAAACACTTATTCGCTTATGTTGGAGTGCCATCTCGAGTCCGACGGGATGCGCCTGCGCATGAGCTATGATCGCCTGGTCATCGACG	ADLAQVWAWNATVPQTVQGSVNDLIAQRV
	CGGAGCAGGTTAAGAGGATAGCCAGGCAGTTTGAAGGGGTGCTGCGCCAGGTCTGCAGTGAAGTGAATGCGCAGGAGCTGGTGGGC	QQQPDALAICAWDGQLSYQELDTLSTQLALS
	ACTGTCTCAGCTGCCAGTGAAGCCGACCTGGCCCAGGTGTGGGCCTGGAATGCAACTGTCCCCCAGACCGTCCAGGGCTCTGTCAACG	LVQQGAGRGSVIPLCFEKTMWTPVAMLAG
	ACCTCATTGCGCAACGCGTTCAGCAACAGCCCGATGCCCTGGCGATCTGCGCGTGGGATGGACAGCTGAGCTACCAGGAACTCGATACC	MKAGSTVVTMDPSQPEDRLQSIIQQTQPPLI
	CTCTCCACGCAGCTGGCCCTATCGCTCGTGCAACAGGGGGCAGGCCGTGGCTCGGTGATTCCCTTATGCTTCGAGAAGACAATGTGGAC	LTSDTYKPLASRLAGVVIRVNRDTLQALPQHD
	GCCCGTGGCCATGCTGGCTGGCATGAAGGCTGGCAGTACTGTGGTTACAATGGACCCCAGCCAGCCGGAGGATCGATTGCAGTCAATC	LDTPDLPTIHPTDGLYIAFTSGSTGNPKGAVM
	ATCCAACAGACCCAACCGCCACTCATCCTCACCTCCGACACATACAAGCCTCTGGCATCTCGACTCGCAGGGGTGGTCATCCGCGTGAAT	SHQNIRSAIHHSTGALGFTPTTRVLGFSSYAF
	CGGGATACCCTGCAGGCACTGCCACAGCATGACCTTGATACTCCAGATCTCCCAACCATCCACCCGACGGACGGACTCTACATCGCGTTC	DAVWVEFLYAMAVGGCLCIPSDAQRNSGDL
	ACCTCGGGGAGCACTGGAAACCCCAAGGGGGCAGTCATGTCCCATCAGAACATTCGCAGCGCAATCCACCACAGTACGGGCGCCTTGG	AGCMEGLRVDLALLTPSTARLLDAEAVPSLKT
	GCTTTACGCCTACCACCCGGGTGCTTGGATTTTCCTCCTATGCATTTGACGCGGTGTGGGTGGAGTTTCTCTATGCGATGGCCGTCGGGG	LVLIGEPVTSDDLARWTGNVDLRNGYGPAEC
	GCTGTCTGTGTATCCCTTCGGACGCGCAGAGAAACAGTGGTGATCTTGCAGGCTGCATGGAAGGGTTGCGAGTGGACCTTGCTCTCCTG	SAITAAYKFQGPNDQPSIVGHAVGLVAWVVE
	ACCCCGTCCACGGCCCGGCTTCTCGACGCAGAGGCAGTCCCCAGCCTGAAAACCCTGGTCCTGATCGGGGAGCCCGTCACGAGCGATG	PSDGASLSPLGAVGELWVEGPLVGKGYHGD
	ACCTGGCCAGATGGACCGGCAACGTTGATTTGAGGAATGGGTACGGCCCTGCAGAATGCTCCGCAATAACCGCAGCCTATAAATTTCAG	PEKTAMSFVHDPVWLLQGTLGYPGRPGRLY
	GGTCCAAATGACCAGCCCAGTATCGTTGGCCACGCGGTGGGCCTCGTGGCTTGGGTGGTTGAACCATCGGACGGCGCCAGCCTCAGCC	KTGDLVRYTSDGKLVCVGRKDTQVKIRGQRV
	CCCTCGGAGCGGTGGGGGAGCTGTGGGTGGAAGGGCCGTTGGTGGGAAAGGGGTATCATGGGGATCCAGAGAAGACAGCCATGAGT	ELAEIEHYIKEATRASVVVDMARPQGSRGPV
	TTTGTCCATGACCCCGTCTGGCTGCTCCAGGGAACACTGGGCTATCCTGGGCGGCCAGGTCGACTCTACAAGACCGGCGATCTTGTTCG	LVAYVALGQVAAVGSPEAVRTALRRCIQGVED
	ATATACCTCCGATGGCAAGCTGGTTTGCGTGGGGAGAAAGGACACGCAGGTCAAGATCCGCGGCCAGCGGGTCGAACTGGCCGAGAT	HLSKHLPRYMVPSFYIPVVDIPLTATGKTDRR
	CGAGCATTATATCAAAGAGGCCACCAGAGCTTCCGTCGTGGTGGATATGGCCAGGCCCCAGGGCAGCAGAGGCCCCGTGCTGGTGGCC	ALRNTGSSFAIDQLAELQPSGGQKRTLPQSE
	TATGTGGCTTTAGGGCAAGTTGCAGCCGTGGGCTCCCCCGAGGCAGTGAGGACGGCACTTCGGCGCTGCATCCAGGGGGTTGAAGATC	MERSLQQLWAEVLNINPSQIGMDESFFLLG
	ATCTGAGCAAGCACCTGCCCCGGTATATGGTGCCGAGTTTCTATATCCCCGTGGTCGATATCCCATTAACAGCCACGGGCAAGACCGACC	GDSISAMQVSAKSRARGVRVTVPDIFKFKTIA
	GACGGGCATTACGCAATACCGGATCCTCCTTCGCCATCGACCAGCTGGCAGAGCTGCAGCCCTCTGGAGGCCAGAAGCGCACCCTGCCC	RLARREIQIDDLAIDDREMLDTPFALSPIQQF
	CAGAGTGAGATGGAGCGGAGCCTGCAGCAGCTCTGGGCGGAGGTGTTGAACATCAATCCCTCTCAGATCGGCATGGATGAGAGCTTTT	FFDAQKDKQGHFNQSFLVRTTKPQQPDAVL
	TCCTGCTCGGGGGGGACTCTATCTCGGCCATGCAGGTCTCTGCCAAATCTCGCGCGAGGGGGGTTCGCGTCACAGTGCCAGATATCTTC	RAVQFIVARHSMLRARFHRGTDGVWTQQIT
	AAATTCAAGACAATTGCGCGTCTAGCACGACGCGAGATCCAGATCGATGACCTGGCCATTGACGACCGAGAGATGCTCGACACCCCCTT	SRADESYACRHHRLASVEDAVPVLNSSQRSL
	TGCCCTATCTCCCATCCAACAGTTTTTCTTCGACGCCCAGAAAGACAAGCAGGGTCACTTCAATCAAAGCTTCCTCGTGCGCACTACCAA	DVQAGPIFAVDLIDTPPDCQYLFLTAHHLVVD
	GCCCCAACAGCCCGACGCTGTACTGCGCGCCGTGCAATTCATTGTTGCCCGACACTCGATGCTGCGTGCTCGCTTCCACCGGGGCACCG	LVSWRIILEELEEYLLTGTVAGSSAPLSFQTWC
	ATGGGGTGTGGACGCAACAGATCACCTCGCGTGCTGATGAATCCTATGCTTGCCGTCATCATCGCCTGGCTTCGGTGGAGGACGCCGTTC	RLQERHAQDHLTPGTAFPFDIQPPCEAYWGL
	CGGTGCTGAATAGCAGCCAGCGGTCCTTGGACGTCCAGGCTGGCCCCATATTTGCGGTTGATCTGATCGACACTCCTCCAGACTGTCAGT	SPELNTHDAIQEAGFTLSKKTTSILLGPANHAF
	ACCTGTTTCTCACGGCGCACCATCTCGTTGTCGACCTTGTCTCCTGGCGGATTATCCTGGAGGAGCTGGAGGAGTACCTGTTGACTGGCA	QTQPIELFLAALLHSFMDSFTDRDPPTIFNEG
	CTGTTGCTGGTTCCTCTGCGCCACTCTCATTCCAGACCTGGTGCCGGCTTCAGGAGAGACACGCGCAGGACCATCTGACCCCCGGTACT	HGREPWNSAIDLSRTVGWFTTLAPISVEAGS
	GCCTTCCCGTTCGATATTCAGCCCCCGTGTGAGGCGTACTGGGGACTATCGCCGGAGCTCAATACGCACGATGCTATCCAAGAGGCCGG	HHLSIPQFVRYIKDRRRQIPHNGWSYFTSRYL
	GTTTACCCTCAGCAAGAAAACGACCAGCATACTCCTGGGACCAGCGAACCATGCGTTCCAAACTCAGCCCATCGAACTGTTCCTAGCCGC	NTEGKTVFSRHACPEVTFNYFGLYQQLERSD
	GCTGCTGCACTCTTTCATGGACAGCTTCACTGACCGGGATCCTCCTACCATCTTCAACGAGGGGCACGGTAGAGAGCCATGGAACTCAG	ALFQSCPSLQDRVLDVADRMARFALIDVSAE
	CAATCGACCTATCCCGGACGGTTGGCTGGTTTACCACCCTGGCGCCAATCTCAGTGGAAGCGGGCTCCCATCACCTTAGCATCCCCCAGT	VTQDCLQFRFLSSQRMQKQDALARWIAACE
	TCGTCCGTTACATCAAGGATCGCCGGCGACAGATTCCTCACAATGGGTGGTCGTACTTCACCTCACGGTACTTGAACACCGAGGGGAAG	RLLEATAVQLLDMQPSYTLADFPLLPPTDAT
	ACGGTCTTTAGCCGCCATGCCTGCCCTGAGGTTACGTTCAACTACTTTGGCCTCTATCAGCAACTGGAGAGGAGCGACGCGCTCTTCCAA	WKQLETLPRLGLAYGDVEDIYPCSPLQQGILL
	TCGTGTCCCAGTCTGCAGGACCGAGTGCTGGATGTTGCAGACCGCATGGCCCGGTTTGCCCTGATTGATGTTTCTGCCGAGGTCACGCA	SQAKSPEMYWTRVRWVVRSSTGASSPVDA
	AGACTGCCTGCAGTTCCGGTTTCTTTCCAGCCAGCGCATGCAGAAGCAGGACGCCCTGGCTCGGTGGATTGCGGCGTGCGAGCGTTTG	DRLERAWRKVVARHAALRTRFSDSPFSDGYS
	TTAGAAGCCACAGCCGTGCAGCTGCTAGATATGCAACCCAGCTACACGTTGGCCGACTTCCCATTGCTGCCCCCTACAGATGCAACGTGG	YQIVLKDPPPTIHTIQAADPMDAVVEYRAAS
	AAGCAGCTGGAGACGCTCCCACGGCTTGGCCTTGCCTACGGTGACGTGGAGGATATCTATCCATGCTCGCCCTTGCAGCAGGGGATTCT	GSQVRPVHSLVLCPMASGDLVCDLQINHAII

	GTTGAGTCAGGCAAAAAGCCCGGAGATGTATTGGACCCGAGTGCGTTGGGTTGTCCGGTCCAGTACGGGGGCATCATCTCCGGTTGAT	DAISIRVLKRELCAAYDDALPADPGPRYSDYIS
	GCAGACCGACTGGAACGTGCCTGGCGGAAGGTCGTCGCACGCCATGCTGCACTCAGGACTAGATTCTCTGACAGCCCCTTCTCAGATGG	HLQSLPTTEAKEYWQTQLAGAQPCIFPTLNE
	ATATAGTTACCAGATAGTCTTGAAAGACCCTCCCCCCACTATTCACACCATCCAGGCTGCGGACCCGATGGACGCCGTTGTCGAGTATCGA	PIAEPKDAAAVTSLPISPETDQALRQFCRSHA
	GCAGCCAGCGGGTCGCAGGTCCGGCCAGTGCACTCTCTGGTCCTTTGCCCTATGGCCAGCGGAGACCTCGTGTGTGACCTGCAGATCA	LTPANVFSLAWMLVLRSYTSSESVCYGYLISGR
	ACCACGCCATCATCGATGCCATATCCATCCGGGTTCTGAAACGCGAGCTGTGTGCCGCGTACGACGACGCCCTCCCTGCGGACCCAGGG	DVPIHQVDRAVGPFINMVVNHVEIDSNRTLL
	CCCCGGTACAGTGACTATATCAGCCATCTCCAGTCCTTGCCGACGACGGAGGCGAAAGAATACTGGCAGACGCAGTTGGCTGGCGCTCA	AMLQEVQAGYLGSLKYNQYPLAEILHDLSTE
	GCCATGCATCTTCCCTACCCTGAATGAGCCCATCGCCGAACCCAAGGACGCGGCCGCCGTTACATCTCTGCCGATCAGCCCGGAGACGG	GQPFFNTVLSVQSGGRADSAQPPDTPTITLE
	ACCAGGCCTTGCGCCAGTTCTGCCGAAGCCATGCCCTGACCCCGGCCAACGTGTTCAGTCTCGCGTGGATGTTGGTCCTACGCAGCTAC	NETWHDPNEYDIAASVLLLDDNPRITLNYSR
	ACCAGCAGCGAGTCCGTCTGCTACGGCTATCTGATCTCTGGCCGGGATGTGCCCATACACCAGGTAGATCGAGCGGTTGGACCGTTCATT	HLLSERQASAVGATFVEALANIVRHPDRNLC
	AACATGGTGGTCAACCATGTCGAGATAGACAGCAACCGAACCCTCCTAGCAATGCTGCAAGAGGTTCAGGCAGGGTACCTAGGAAGTCT	DLETTISPQDLATIWDWNAKVPATLSSSVPEL
	GAAGTACAACCAATATCCGCTGGCCGAGATCCTGCATGACCTCAGCACCGAGGGGCAGCCCTTCTTCAATACGGTTCTGTCGGTGCAGA	FSRWVKQQPDAQAVCAWDGDLTYRQLDEG
	GTGGCGGCAGGGCCGACTCAGCCCAGCCCCCAGACACACCCACCATCACCCTCGAGAACGAGACGTGGCACGATCCGAACGAATACGA	SSRLAHHLLARGVRPQSILPLCFEKSRWVPVA
	CATTGCCGCATCGGTCCTGCTGCTGGACGACAATCCCAGAATCACGCTGAACTACTCCCGCCATCTGCTTTCGGAGCGACAGGCCAGTGC	MLGVLKAGCAVVTMDPEQPVERLQLVVQKT
	CGTCGGGGCGACGTTTGTGGAAGCCCTGGCCAATATCGTCCGCCATCCGGACAGAAACCTCTGCGATCTGGAGACGACGATCAGTCCCC	QGVILTSPACQGLASQLRPEAVVVDGRSLER
	AGGATCTCGCCACCATCTGGGACTGGAATGCCAAGGTTCCCGCGACCTTGTCCAGCTCGGTGCCCGAGCTGTTCTCCCGATGGGTGAAG	MPQPDPLSLPTIHPTDRLFLVFSSGTTGTPKG
	CAACAGCCTGATGCGCAGGCCGTCTGTGCCTGGGATGGAGACTTGACCTATCGTCAACTCGACGAGGGGTCGTCCCGGCTGGCTCACC	SVMSHQNACSAVHHQQARLGLPPAARMID
	ATCTGCTGGCTCGGGGTGTCCGCCCGCAGTCAATACTGCCCCTGTGCTTCGAGAAATCCCGGTGGGTGCCCGTGGCCATGCTGGGGGTG	CLSYAFDAPWFNFLHAFSSGGCLCVPSDQQR
	CTCAAGGCAGGCTGCGCGGTAGTCACGATGGATCCGGAACAGCCCGTGGAGCGCCTACAGCTCGTGGTGCAGAAGACTCAAGGGGTG	KDNLAGCIESLKANYALLTPSIARVLDPAAVPG
	ATTCTGACATCGCCAGCCTGTCAGGGCCTAGCATCCCAGCTACGTCCTGAGGCTGTCGTCGTGGATGGGCGATCGCTTGAGAGGATGCC	LETLAFGGEAIRAEDISRWHEVKLMGYYGPS
	TCAACCAGACCCGCTGTCTCTGCCCACGATCCATCCCACGGACAGGCTCTTTCTGGTGTTTTCCTCCGGCACCACAGGGACCCCCAAAGG	ECTVVSTIHQFESKTEEPRMLGYSSGLTAWVV
	GTCTGTGATGAGCCATCAGAACGCATGCTCGGCAGTTCACCACCAGCAGGCACGTCTTGGGCTGCCGCCCGCAGCCCGAATGATCGATT	DPLQGRRLAPLGATGELCVEGPLVGQGYIDE
	GCCTCTCATACGCGTTCGACGCTCCCTGGTTCAACTTTTTGCATGCCTTTAGCTCAGGCGGTTGTCTGTGTGTTCCGTCCGATCAACAGCG	PQKTAASFVEDPSWLLRGGGPDFPGRRGRV
	GAAGGACAATCTGGCGGGCTGTATCGAGAGCCTCAAGGCCAACTACGCGCTTCTTACGCCCTCGATTGCCAGAGTCCTGGACCCTGCAG	YKTGDLVRFRPDGSLLYVGRKDTQVKIRGQR
	CGGTGCCTGGCCTGGAGACACTTGCATTCGGCGGGGAGGCTATACGCGCGGAGGATATCAGTCGATGGCACGAGGTCAAGCTGATGGG	VELGEVEHHIRQTLPAGVDVPVVVELVHPHA
	GTATTATGGTCCCTCGGAGTGCACTGTCGTCTCAACCATCCACCAGTTCGAGAGCAAAACAGAGGAACCCAGAATGCTCGGTTATTCTTC	SANPVLVAFLPVGKEAAGSSESMRAVLDRYT
	TGGGCTGACAGCCTGGGTGGTGGACCCGTTGCAGGGGAGACGCCTGGCACCGCTTGGTGCGACAGGAGAGCTCTGTGTCGAGGGTC	HGVEDRLMARLPSYMVPRMYIPVPAIPFTTT
	CGCTTGTCGGACAAGGGTATATCGATGAGCCCCAGAAGACTGCGGCCAGCTTTGTGGAAGATCCCAGCTGGCTTCTCCGTGGTGGAGG	GKTDRRSLQRVASSYTLEQLAALQPSRDTRR
	GCCAGACTTCCCTGGACGTCGGGGCCGGGTGTATAAAACAGGAGATCTCGTCCGATTTCGTCCCGATGGATCGCTCCTGTATGTCGGGC	APTTAMERRLQGMWATILDIDAVTIAATDSF
	GCAAGGATACTCAAGTGAAGATCCGGGGCCAGCGGGTGGAGTTGGGCGAGGTTGAGCATCACATCCGCCAGACGCTGCCAGCCGGCG	LRIGGDSIGAIRLVRLAAEQDILLTVAAIFKSPIL
	TGGATGTGCCGGTGGTTGTGGAACTGGTCCACCCCCATGCAAGTGCGAATCCGGTGCTGGTGGCCTTTTTGCCCGTGGGAAAGGAAGC	CDMAQVATLGSTSGPHDIPPFSLLKQDVDIS
	TGCTGGGTCATCCGAGTCAATGCGTGCAGTCCTGGACCGCTATACGCACGGGGTGGAGGACCGCCTGATGGCCCGACTGCCCAGCTATA	QARLQVAAWCELSPSAVEDILPCTPLQEGLLS
	TGGTCCCTCGCATGTACATCCCCGTGCCAGCGATCCCATTCACGACGACGGGGAAGACAGACCGGCGCAGCCTGCAACGGGTCGCGTC	LTVKNQGAYVNRQVLQLRDEVDLGRFQTAW
	TTCGTATACCCTGGAGCAGCTGGCAGCGCTTCAGCCCTCGCGGGACACTCGTCGTGCGCCCACAACGGCGATGGAGCGGCGTCTGCAA	NQVAMATSILRTRIVDLPGQGLVQVVTSEVP
	GGGATGTGGGCTACTATCCTGGATATCGATGCAGTGACCATCGCGGCCACCGACAGCTTCCTGCGGATTGGAGGTGACTCAATCGGAGC	RWNHGRSLDALVQDDLQRPVTLGTPLATFG
	CATTCGACTCGTGCGACTGGCTGCAGAGCAAGACATTCTGCTCACGGTGGCAGCCATCTTCAAATCTCCCATTCTGTGTGACATGGCGCA	LVDAGDVGGDRQRYFLLTLHHALYDGWSLSL
	GGTGGCGACACTGGGATCGACTTCCGGACCTCATGACATCCCCCCATTTTCACTGCTCAAGCAAGACGTGGATATCTCGCAGGCGCGATT	LLDEVSKAYHGTASDGLVSFKTMIKYVTELGA
	GCAGGTGGCGGCGTGGTGTGAGCTCAGCCCGTCTGCTGTCGAGGATATCCTTCCGTGTACACCTCTCCAGGAAGGTCTGCTTTCTCTGA	EADSYWQGSLDGLVAEPFPALPSPMYQPRA
	CGGTCAAGAATCAGGGCGCCTATGTGAACCGCCAGGTCCTGCAACTTCGAGACGAGGTAGACCTTGGACGCTTCCAAACCGCCTGGAA	QDILEHNVLGLRWLQNHHITPATTLRAAWAI
	TCAAGTCGCCATGGCCACGTCGATCCTGCGAACCCGCATCGTGGACCTTCCGGGGCAAGGGTTGGTGCAAGTCGTTACCAGCGAGGTG	LTTHYTQSADVLFGSTVTGRQAPIHRVGLVEG
	CCACGGTGGAACCATGGCCGTAGCCTCGACGCACTCGTGCAAGACGATCTGCAACGTCCGGTAACCCTTGGTACCCCCTTGGCCACATT	PTIATVPVRIAIQEKATLAGLLDQVQEQSVDM
	TGGCTTGGTGGACGCCGGTGACGTTGGTGGGGATCGGCAACGGTACTTCCTGCTAACGCTTCATCATGCGCTCTATGACGGATGGTCTCT	IPYEQVGLQRIRRLSADTERACQFQTLLVVQP
	GTCGCTGCTGCTGGACGAGGTCAGCAAGGCCTACCACGGGACCGCGAGCGACGGCCTTGTCTCCTTCAAGACAATGATCAAGTATGTG	APEPSATDGPSALFHAEDDTLSQAALSSFNSY

	ACAGAGCTGGGTGCCGAGGCGGACTCCTACTGGCAAGGCAGCCTGGATGGTCTGGTGGCCGAGCCATTCCCTGCCCTGCCCTCTCCCAT	ALLLQCQLTATGVSMQMSYDSHVMAQPQV
	GTACCAGCCACGCGCGCAAGACATCCTGGAGCACAACGTATTAGGGCTACGGTGGTTGCAAAACCACCACATCACACCGGCAACCACTC	QRLARQFEHLVRLLCDESQHSSSVAHIDAVCE
	TTCGCGCGGCCTGGGCCATTCTCACAACCCACTACACCCAATCTGCGGATGTCCTTTTTGGATCGACCGTGACTGGACGGCAGGCTCCCA	DDLRRIWALNSPPPARVDACMHDLITEQAQ
	TTCACCGCGTGGGACTGGTCGAAGGGCCGACAATTGCAACTGTGCCGGTCCGCATTGCCATCCAGGAAAAGGCTACCCTGGCAGGCCT	RRPVSQAVAAWDGELTYRELDELSTQLAYGL
	ACTCGACCAGGTCCAAGAGCAGTCTGTCGATATGATTCCCTACGAACAGGTCGGGTTGCAGCGAATCCGGAGGCTCAGCGCCGACACC	VNLGVGPHAVVALCFEKSMWMPVAMLGV
	GAGCGAGCTTGTCAGTTCCAGACGCTCCTGGTGGTGCAGCCAGCCCCGGAACCGAGCGCCACAGATGGACCCTCTGCACTGTTCCATG	MKAGGASVAMDITQPEDRLRTVVQQVQPP
	CAGAAGATGACACTCTCAGCCAGGCGGCTCTCAGCAGCTTCAATTCATACGCTCTCTTGCTGCAATGCCAGCTGACGGCCACGGGGGTG	LVLSSLEAEDLARRLSDRPVHVVSQDRLQTTS
	TCCATGCAGATGAGCTACGACTCGCATGTCATGGCACAACCGCAGGTGCAACGGCTGGCCCGGCAGTTCGAGCATCTAGTACGACTCCT	LGSGQGGQLPPVQPTDRLYIAFTSGSTGVPK
	TTGCGATGAGTCTCAGCACTCATCCTCTGTTGCTCACATAGATGCGGTCTGCGAGGATGATCTGCGAAGGATCTGGGCGCTGAACTCCCC	GAVMSHQNFTSAVHHQTALEFTASARVFDF
	GCCCCCGGCCAGAGTGGACGCCTGCATGCACGACTTGATCACCGAGCAGGCCCAGCGGCGGCCAGTCTCACAGGCCGTCGCTGCATG	SSYAFDACWLNFLHTMAAGACLCIPSEEERK
	GGATGGAGAGCTCACGTATCGCGAGCTGGACGAGCTCTCCACCCAGTTGGCATATGGCCTCGTCAACTTGGGGGTCGGCCCCCATGCG	SDITGCMRRLAVTYANLTPSTARLIDPTSVPSL
	GTGGTCGCTCTGTGCTTTGAGAAATCAATGTGGATGCCCGTGGCAATGCTTGGAGTCATGAAAGCCGGCGGTGCATCGGTGGCCATGGA	QTLVLVGEPVAQQDITQWKAHVQLKNGYGP
	TATCACGCAGCCAGAGGACCGGCTTCGCACGGTAGTGCAGCAGGTACAGCCTCCTCTGGTCCTGAGCTCCCTGGAGGCAGAGGACCTG	AECSAISTTFDLGQSDHDPATIGAGRGMVT
	GCACGGCGTCTGAGCGATAGGCCGGTGCACGTTGTCTCTCAAGACAGACTGCAGACGACGTCGCTGGGCAGCGGCCAGGGCGGCCAA	WVVEPTESRHLSPYGAVGELWVEGPLVGLGY
	TTGCCCCCAGTTCAGCCGACAGACAGGCTGTATATTGCCTTCACCTCAGGTAGTACGGGGGTCCCGAAAGGCGCGGTCATGTCCCACCA	LGRPDLSAASFVDNPPWLLRGGPQGFPGRH
	GAACTTCACCAGTGCCGTGCACCACCAAACCGCGCTCGAGTTTACTGCGTCGGCCAGGGTGTTTGACTTTTCGTCCTACGCGTTCGATGC	GRLYRTGDLVRYNLDGTLVCIGRKDAQVKIRG
	TTGCTGGCTGAACTTTCTCCATACGATGGCGGCGGGTGCCTGTCTCTGCATCCCCTCCGAAGAGGAGCGCAAGAGCGACATCACTGGCT	QRVELAEVEHHLHQALPSAAVDVSVAVEVIT
	GCATGCGCCGCCTGGCCGTGACTTATGCGAACCTCACGCCATCCACCGCACGTCTGATCGATCCGACGTCCGTCCCCAGCCTCCAGACCC	LQGSANPLLVAFVAMGEAALGPRETVRAKLA
	TCGTTCTGGTCGGAGAGCCGGTGGCACAGCAGGACATCACTCAGTGGAAAGCCCACGTCCAGCTGAAGAACGGTTATGGCCCTGCGG	LYSQGARERMADQLPVYMVPSLLLPAVEIPT
	AATGCAGCGCCATCTCCACCACGTTCGATCTCGGGCAAAGTGACCACGACCCGGCAACCATCGGCGCCGGCAGAGGAATGGTGACCTG	TATGKRDRRRLREMWASKSLEELAELQPTKG
	GGTGGTTGAGCCGACCGAAAGCCGACATTTGTCGCCGTACGGGGCTGTGGGCGAGCTGTGGGTGGAAGGTCCTCTGGTTGGATTAGG	NHQAPTTDVERRILQLWAECLNISASKISIHD
	GTATCTCGGCCGTCCGGATCTCAGTGCTGCAAGCTTCGTCGACAACCCCCCATGGCTGCTGCGCGGGGGACCCCAGGGGTTCCCAGGTC	SFFALGGDSISAMQLSAKGRSIDLQMTVGDIF
	GACATGGCCGGCTTTACCGCACCGGTGACCTGGTGCGGTATAATCTGGACGGCACCCTGGTCTGTATTGGCCGCAAAGATGCCCAGGTC	KYKTIARLALSISPAVESAVLHAPEGQGASFAL
	AAGATCCGCGGACAGCGCGTGGAGCTGGCCGAGGTCGAGCATCATCTGCACCAGGCCTTACCTTCGGCCGCCGTGGACGTCTCTGTGG	SPIQQMFADMQQGVSNHFNQSFFVQVRRP
	CAGTCGAGGTCATCACCCTGCAGGGCAGTGCCAATCCACTGCTAGTCGCCTTTGTGGCGATGGGGGAGGCGGCCCTGGGCCCGCGGG	VTLPQIQAAVDALVAHHGMLRARFKCSRENI
	AGACTGTCCGAGCAAAGCTGGCTCTTTACAGCCAGGGGGCCAGGGAGCGCATGGCAGACCAGCTGCCCGTCTACATGGTGCCCAGTCT	WSQWILPPGTTGSYRVCQHEVAGLQAASAV
	GCTTCTTCCGGCGGTTGAAATTCCCACGACGGCCACGGGCAAAAGAGATCGACGGCGGCTGCGTGAGATGTGGGCCTCCAAGTCCCTG	INHSQESLDIQNGRLMAVDLINADEGQYLFL
	GAGGAACTGGCCGAGCTGCAGCCGACCAAGGGCAACCACCAAGCACCGACGACAGACGTCGAACGGCGGATTCTGCAACTGTGGGC	VVHHMVVDLVSWRIILADLEEHLTTASLSGFT
	CGAGTGCTTGAATATCAGCGCATCGAAGATCAGCATTCACGATAGCTTCTTCGCCCTTGGCGGGGACTCAATCTCCGCCATGCAGCTCTC	SMSFQTWCQLQADHHAESPFELEAVLPNGA
	GGCAAAGGGCCGCTCCATCGACCTCCAGATGACTGTAGGGGACATTTTCAAGTACAAGACAATCGCTCGACTGGCTCTCAGCATCTCCC	PPPLLPQLDYWGQVRSLNTFDNMIKDGVVL
	CAGCAGTGGAATCGGCCGTGTTACATGCCCCAGAGGGCCAGGGTGCCTCCTTTGCCCTGTCTCCTATCCAGCAGATGTTCGCTGACATGC	GRQDTETLLGPANAAFDTQMVELLHAAILHS
	AGCAGGGGGTTAGCAACCATTTCAACCAAAGCTTTTTCGTGCAGGTACGTCGACCGGTCACCCTGCCTCAGATCCAGGCCGCTGTGGAT	FANVFHDRAPPTVFSEGHGREPWRSAIDISR
	GCCCTGGTCGCCCATCATGGCATGTTGCGCGCGCGTTTCAAGTGCAGCAGGGAGAATATCTGGAGCCAGTGGATTCTGCCCCCGGGGA	TVGWFTTMFPVVATAKKGDSIASIVRHVKDR
	CCACAGGAAGCTATCGCGTTTGCCAGCATGAGGTTGCGGGTCTTCAGGCAGCCTCGGCGGTGATCAATCACAGCCAGGAGTCGCTAGA	RRQIPDNGRPYFASRFLTPAGKRAFQVNGPV
	CATCCAGAACGGTCGGCTCATGGCGGTCGATCTGATCAATGCAGACGAGGGCCAATACCTTTTCTTGGTGGTCCACCACATGGTCGTCGA	EVIFNYLGLYQQLERSDALFHMREMPDGVD
	CCTGGTGTCTTGGAGAATCATCCTGGCTGATCTGGAAGAGCACTTGACCACCGCATCCCTCTCCGGGTTCACCTCGATGTCCTTCCAGAC	DMADISGSLFRFALVDISASVTDGCLHVDFM
	CTGGTGCCAGTTGCAGGCTGACCACCACGCCGAGAGCCCCTTCGAGCTGGAGGCTGTCCTGCCAAACGGGGCTCCGCCTCCGCTGCTG	YNRHMQHQASIRAWIKECRQSLHAAAQELP
	CCACAGCTAGACTACTGGGGTCAGGTCCGCAGCTTAAACACATTCGATAACATGATCAAGGATGGCGTGGTTCTCGGCAGGCAGGACAC	LIQPSYTLCSFPLLRMTDPALAILQQRLTELDL
	AGAGACCTTGCTAGGGCCTGCGAATGCGGCATTTGACACACAAATGGTGGAGCTCCTCCATGCGGCCATCCTACATTCCTTTGCCAATGT	AYGQVEDIYPCSPLQDGILMSQIKNPDLYRTR
	CTTCCACGACCGGGCACCTCCGACGGTATTCAGCGAAGGCCATGGTCGGGAGCCCTGGAGGTCTGCAATCGATATTTCACGCACGGTCG	IRWMAQSAQGSTAVDTNRLKHAWQQVVD
	GGTGGTTTACCACGATGTTCCCTGTGGTCGCCACAGCCAAGAAGGGAGATAGTATTGCCAGCATTGTGCGCCATGTCAAGGACCGTCGA	RHPALRTIFVDSISGRGLKDQVVVRNLRADV
	CGCCAGATACCCGATAATGGCCGGCCATACTTTGCAAGCCGCTTCCTCACTCCGGCTGGCAAGCGGGCGTTCCAGGTCAATGGCCCTGTC	HIVQSADEASIEDMSPTKGKTSSVLTLSTTNS

	GAGGTGATTTTCAACTACCTCGGGCTGTACCAGCAACTCGAGCGGTCGGACGCCCTCTTCCACATGCGCGAGATGCCGGACGGGGTGG<br>ATGACATGGCTGATATCTCGGGGAGCCTGTTCCGATTTGCTCTGGTGGATATCTCGGCATCGGTGACGGATGGCTGTTTGCATGTTGACTT<br>CATGTACAATCGGCACATGCAACACCAGGCCTCCATCCGCGCGTGGATCAAGGAATGCAGGCAGTCCCTCCACGCCGCAGCGCAGGAG<br>CTTCCTCTGATACAGCCAAGTTATACCCTCTGCAGCTTCCCGCTGCTGCGCATGACAGACCCGGCATTGGCAATCCTGCAGCAGAGGCTG<br>ACGGAGCTGGACCTCGCATACGGGCAGGTCGAAGATATCTACCCCTGCTCGCCGCTCCAGGATGGAATCCTCATGAGCCAGATCAAGAA<br>CCCAGACCTCTACAGGACACGCATCCGGTGGATGGCACAGTCTGCACAGGGATCAACAGCAGTTGATACCAACCGGCTCAAGCACGCG<br>TGGCAGCAGGTGGTAGACCGCCATCCAGCCCTGCGCACCATCTTCGTCGACAGCATCTCGGGCAGGGGACTGAAGGATCAAGTGGTGG<br>TGAGGAATCTCCGGGCAGACGTCCACATCGTCCAGTCCGCGGACGAAGCAAGTATCGAGGACATGTCCCCAACAAAAGGCAAAACCAG<br>CTCAGTGCTCACTCTGTCGACGACGAACTCGGGCGTCCTGTGCGAGCTATCGATCAGCCATGCGCTCATCGACGCCTTTACCTTGGGAAT<br>CCTCAAGCAGGAGCTGTGTGCCGCATATACTGGCCTCCTTTCCTCTTCCCTGGCACCCCTGTATAGCGACTACATCCACTTCATCCAGTCCC<br>TGCCCGCTGGGTCAGCCGCGGCATACTGGCAGGAGCATCTCCAGGAGGTCAAGCCCTGTCTATTTCCATCCTTGGGCGGTCTAAATACG<br>GAAGGCCGGCGGTCGCAAGCTCACATCCCTATTACCTTCGAGCGAGAGCTACACCTGGCATTGCGCATCTTTTGCATGGAGCATGGACT<br>GACGGTGTCTAATATCTTCCATGTCGCTTGGGCCCTGGTCCTGCGGGCTTATACTGGTTTGGATACTGTCTGCTTTGGCTATCTCACATCAG<br>GCCGAGATATTCCTCTGCAAGGGGCTGACGGAACCGTTGGTCCATTTATCAATATGCTTGCCAGCCGCGTGGACCTGGGCAGTAAGGAC<br>TCGCTGATGACCCTGGTACAGAGGGACCAGGAGCAGTATCTCAACAGTTTGGAGTTTCAGCATTACCCGCTGGCCAAGATCTTCCACTT<br>GATTGATACGCCCGAGAAGGGGCTTTTCAACACCGCCATGTCAGTCCAAGCTAGTGACTCTGGTCCCAAGGACTGCCAATCGGCTATATC<br>GCTTGTAGATGAAGGAGGAGATGATCCCACCGAGGTAAGAAAGATAAGAAGACTTTTTCCTTTGTGTGACTGCGCTAATGCGAGACTCA<br>GTATGATATCATGATAAACATTGGCGTCGGAGACGAGGACACCGGATGCAACTTTACCTTTAACGAATCGGTGATATCTGACCGCTATGCA<br>AAGAGTGCCATCGACCTGTTCTTGCACGCCGTCTCACATATTGTTCAGCACGCGGACCAGACGGCCCAGGAAGCCAATTTCATCAGCAA<br>GCAAGACCTGCAATCGATCTGGCAGTGGAATGCCGCCGTAGCAACGCCTCTGGACTGGTGTGTTCATGAACTTATCATAGAGCAGGCAG<br>AGAAACAACCAGCGGCGGCTGCCATCTGCGCGTGGGATGGTAATCTCACGTATAAGCAACTCAACGATCTGTCCACGCAGCTGGCATGC<br>CACATCCGACAACTGGGTGTTGGGCCAGGGGTTAATGTCCCGTTGCTCTTTGAGAAGTCGCGGTGGATGTCGGTTGCCAGTCTGGCGGT<br>CATGAAGGCTGGTGGAACCATGGTGGGCTTGGATCCTGGTCAGCCTGCAGGGAGACTACGAAGTATCATCGATCAAGTCCAGCCTTGTC<br>TCATTTTGACTTCGGCCGGAAATAGCAACAGCGCGGCAGCTTTTGCATCCTGCCATGTTGTCCGGGTGGATGACGCCAGTTTGGCCCAG<br>CTGGCCACGCCGTGCAATGCTCTGTTACCCCCGGTTGACCCTGCCAGCAGCCTATACCTGGTATTCACCTCTGGGAGTACAGGCGTGCCA<br>AAGGGTGTGGCCATCAGCCATTCGAACCTCAGCACCGCGATTACTCACCAAAAGCGCATCTTGAAACTTTCTGTCGCGTCGAGGGTACTT<br>GAATTCGCTTCATATGCATTCGATGTCTCCTGGGGTACAATCCTTCACACCCTTGCGGCTGGTGGCTGCGTCTGCGTGCCTGAAGAGTCG<br>GAACGCCGCGGAGATATCAGTGCCGCAATGCGTCGGATGGAGGTAAACTACGCGCATCTTACTCCATCTGTTGCGCGTCTTCTGAACCCT<br>TCCAATGTGCCGCTCCTCCAAACCCTCGTCTTGAGTGGAGAAGCCGTATCGCGAGCGGATATGGAGCAGTGGAGCCGGCACGTCCATCT<br>GATCAACGCGTACGGCCCTGCTGAGGCCGCCGTATGGGTGATATTCGCACACCTCGACTCAGCATTGTCCATGCCCTCCATCGGAAAGGG<br>GGGTGGCTGCACCACGTGGATTGTTGACCCTAGTCGACCCGACCAATTAGCCCCAGTGGGCTGCACGGGTGAGCTCTGGCTGGAAGGT<br>CCACTAGTGGGACGTGGCTATCTTCACGACCCCGAACGAACAGCAGCCGCGTTCATCGAGAACCCGCGATGGTTGCTCCAAGGAGCAG<br>GAGGGGCCCATGGCCCTGGCCGACAGGGACGGCTTTATCGGACTGGAGACTTGGCCCGGTATACACCCGACGGTTCGATTGTTTATATT<br>GGGAGAAAGGATAACCAGATCAAGATCCACGGTCAAAGGGTGGAGTTGGAAGAGGTAGAGAAATATATTGAGCAAGCCATGCTAAACA<br>GCGCCGCAGCACCAGCAGTCCCTGTGGTAGCAGCGGTCGTCACCCCTCAAGGAAGCAAGAAGGCAATTCTGGCAGCTTATCTGGCTCT<br>GGGTGAGCCGGCCACGGGCTCTGTGGAAATTGTGCGCAAATCTCTCAGCAGGTACACTGGGATCATCAACCCGGCCTTGGAAGAGAGC<br>CTGCCGACCTATATGAGGCCGAGCATCTACATCCCCGTGGCGGAGATCCCCACGACTACCAATGGAAAGGCTGACCGCAGCAAACTCGC<br>AACCATGGCATCCAGTCGCACTCTGGCTGAATGGGCTGGCCTTCAGACCTCTGAGGGCAAATGGCGCCCTGTATCGTCTCCGGGGGAGC<br>TGGGGCTGCAGAGACTGTTTGCTGAAGTGCTGAATATGGACCAGAGCCTAGTAGGCATGGACGACAGCTTCTTCTCGCTCGGCGGTGAT<br>TCCATTACCGCGATGCAGCTGTCGGCCAAGTCCCAATCCAGCCTTCTCTATATCACGGTGGGCGATATATTCAAGCACAAGACCGTCGCAC<br>AACTGGCAAGCAATGCAAGGCAGACAGCGAGCACCTCCGTGCAGCTCCCGGAAGTCCCCAACAGCTTGTTCGAGCTGTCCCCGATCCA<br>GCAGCTATTCTTTGCCTCGCAGGACAAGGGCAAGAACCTGTTTAACCAGTCCTTCCTGGTCCGCGTTTCCCGCTCCCTGAATCCCAACGA	GVLCELSISHALIDAFTLGILKQELCAAYTGLLS<br>SSLAPLYSDYIHFIQSLPAGSAAAYWQEHLQE<br>VKPCLFPSLGGLNTEGRRSQAHIPITFERELHL<br>ALRIFCMEHGLTVSNIFHVAWALVLRAYTGLD<br>TVCFGYLTSGRDIPLQGADGTVGPFINMLAS<br>RVDLGSKDSLMTLVQRDQEQYLNSLEFQHYP<br>LAKIFHLIDTPEKGLFNTAMSVQASDSGPKDC<br>QSAISLVDEGGDDPTEYDIMINIGVGDEDTG<br>CNFTFNESVISDRYAKSAIDLFLHAVSHIVQHA<br>DQTAQEANFISKQDLQSIWQWNAAVATPLD<br>WCVHELIIEQAEKQPAAAAICAWDGNLTYKQ<br>LNDLSTQLACHIRQLGVGPGVNVPLLFEKSR<br>WMSVASLAVMKAGGTMVGLDPGQPAGRL<br>RSIIDQVQPCLILTSAGNSNSAAAFASCHVVR<br>VDDASLAQLATPCNALLPPVDPASSLYLVFTS<br>GSTGVPKGVAISHSNLSTAITHQKRILKLSVAS<br>RVLEFASYAFDVSWGTILHTLAAGGCVCVPEE<br>SERRGDISAAMRRMEVNYAHLTPSVARLLNP<br>SNVPLLQTLVLSGEAVSRADMEQWSRHVHLI<br>NAYGPAEAAVWVIFAHLDSALSMPSIGKGGG<br>CTTWIVDPSRPDQLAPVGCTGELWLEGPLV<br>GRGYLHDPERTAAAFIENPRWLLQGAGGAH<br>GPGRQGRLYRTGDLARYTPDGSIVYIGRKDN<br>QIKIHGQRVELEEVEKYIEQAMLNSAAAPAV<br>PVVAAVVTPQGSKKAILAAYLALGEPATGSVEI<br>VRKSLSRYTGIINPALEESLPTYMRPSIYIPVAEI<br>PTTTNGKADRSKLATMASSRTLAEWAGLQT<br>SEGKWRPVSSPGELGLQRLFAEVLNMDQSL<br>VGMDDSFFSLGGDSITAMQLSAKSQSSLLYIT<br>VGDIFKHKTVAQLASNARQTASTSVQLPEVP<br>NSLFELSPIQQLFFASQDKGKNLFNQSFLVRV<br>SRSLNPNELQKAIGVLAARHSMLRARFVQSA<br>DGVWQQKIVNDSAGCYTFRSHHITTLQDME<br>PLLHSSQELLDIVQGPILAVELIDSSHDGQYLF<br>MTAHHLVVDLVSWRILLGDLEEFIRSGTITGF<br>PPFSFQSWSQLQANYARDHLPPKIALPFKVS<br>PPRHEYWGLVPGHDANTLSDSGRGSFTMN<br>KRLTDILMGTANSAFDTQPVEILHAALLYAFA<br>QTFQDREAPPLFTEGHGREAWDSGIDLSRTV<br>GWFTTIFPVAASITQNHSLPEVVRRVKDTRR<br>QTPRNGWSYFTSRYLNTDGRQAFQIKGPVEI<br>IFNYMGLYQQLERPDSLFQQCDIAVTAPPAA

	GCTCCAGAAGGCGATTGGAGTTTTGGCAGCACGCCATTCCATGCTCCGGGCTCGGTTCGTGCAAAGCGCGGACGGCGTCTGGCAGCAG<br>AAGATTGTAAACGATTCAGCTGGCTGCTATACATTCCGCAGCCACCACATCACCACGCTGCAAGATATGGAGCCCCTCCTCCATAGCAGCC<br>AGGAGTTGCTGGATATTGTCCAGGGTCCCATCCTGGCCGTGGAACTGATTGATTCGAGCCACGATGGGCAGTATCTCTTCATGACAGCCC<br>ATCATCTCGTGGTGGACCTTGTCTCATGGAGAATTCTGCTGGGCGATCTGGAGGAGTTCATCCGCTCGGGCACAATCACCGGATTCCCTC<br>CATTCTCATTCCAATCATGGAGTCAACTGCAGGCGAACTATGCGCGAGACCATTTGCCTCCAAAGATCGCGCTGCCTTTTAAGGTCAGTCC<br>GCCCCGGCATGAGTACTGGGGACTGGTCCCTGGGCACGATGCAAACACCCTGAGCGATTCCGGTCGAGGCAGCTTCACGATGAACAAG<br>CGCCTGACCGACATTCTCATGGGCACGGCCAACTCGGCCTTTGATACGCAGCCGGTTGAGATCCTCCACGCAGCCCTGCTGTATGCGTTT<br>GCACAGACATTCCAGGACCGCGAAGCACCGCCTCTCTTCACCGAGGGGCATGGTCGAGAAGCCTGGGACTCGGGCATTGACCTGTCCA<br>GAACTGTTGGCTGGTTTACCACCATCTTCCCTGTGGCAGCTTCGATTACGCAAAACCACAGTCTTCCAGAGGTGGTGCGCCGTGTGAAG<br>GATACACGCCGGCAGACACCACGCAACGGGTGGTCCTATTTCACCTCGCGGTACCTGAATACTGATGGACGGCAGGCCTTCCAGATCAA<br>GGGCCCTGTGGAGATTATTTTTAATTACATGGGCTTATATCAGCAGCTGGAGCGGCCCGATTCCCTGTTCCAGCAGTGTGATATTGCGGTC<br>ACAGCGCCCCCTGCTGCTGCGGCGACTCTCTCGCGGTTTGCCCTGATCGACGTCGCAGCATCTGTTGTGCAGGGTCAGCTGAAGTTCGA<br>GTTCCTATACAACAGGGAGATGAAAGGACAAGACAGGATACTTGAATGGATCAGCAAGACAGAATCCTCACTCAAAGCGGCAGCGGAA<br>GAACTTCTGTTGCAGAGCCCAAGCTATACCATCTGCGACTTTCCCCTGCTCTCTCTCACCGACCAGGGTCTCGACGAGCTTCTCAATAGG<br>GTCCTCCCGGCTGTGGGTCTTTCCTACGGCCAGATCGAAGACATCTACCCCTGTGCGCCGATTCAGGAGGGCATCATCATGAGCCAGGCC<br>AAGAGCCCTGAGCTCTATTGGACTCGAGTGCGGTGGACGGTGCAGTCAACAGGCACGTCGCCAGTCGATCTCGATCGGTTGAGGCGGG<br>CCTGGCAGATGGTCGTGAACCGTCACTCGATTTTACGCACGATATTTATTGACGGTATCGGATCCGGCCAGGTGAAGGACCAGGTGGTCC<br>TCAAGGACTTTCCGGTGGACGTCGAGGTCCTCCATGCCGAGCTGCATCAGCTCGCAGAGCCAGTCACTGGGCGTCAATGGCACACCGAT<br>CTGTCGAGCAGAAGAGACACCCCGCAGCATTCCCTGGTGCTGACGCAAACAGCGTCTGGAGTCGTGTTCTGCGACTTGGAGCTCAACC<br>ATGCGATGGTCGATGCGTATTCTCTAGCCCTTTTGAGGCAGGAGATCTGTGCGGCATATACTGGCTCACTGCCTGCCACACCCGCGCCGG<br>CCTACCAAGCGTATATTGAGCACCTGCAGGGGCTGTCCCTTGTTGAAGGACAACGCTTCTGGCAGACATATCTTGACAACGCTCAGCCCT<br>GTCACTTTCCGGCCCTCGGCCAGCCGGATGTCACAGACGGCTCGAACGCCCGCCGGGCGCTATCCATCTCGTTGGACGCGGCCACTCAT<br>CAAGCCCTGCGTGTCTTCTGCCAGCAGCACGCAGTTACACCCTCGAACGTGTTCTACCTGGCTTGGGGGCTTCTTCTTCGCGCCTACACT<br>GGCTTAGACACAGTGTGTTTCGGCTATCTTACGTCCGGGCGCGATGTCCCTGTCCCAGGGGTCGATAGGATTGTTGGTCCGCTCATTAAC<br>ATGCTTGTCTGTGTTCTGGAATTCAGGACTGGGGCATCGGTGCGATCGGCCATGCAGAAGGTCCAGGAAGACTATCTCGCCGCCCTGCA<br>GTATCAATCTACGCCTCTCAGCAAGATCCTGCAGCTTTCTGGGACGTCCGGTCGGGGCCTATTCAATACCGGCATATCGGTCCAGGGCGG<br>TGCTACTTCCGGCGAACCGGACGAGCATGATATCATAGTGACGGATCAGACAGGGCTGGATTCGCCAGAGGTATGTGTTTTGTTCTTCTT<br>GTCGTGGTCACGATTCCCCTTTGCTCTCCATAGCTGACCCGGTCCCACAGTACGACATTGCCGTTGCTATCTCCCACGATGAGGAGGAGA<br>CGGAGATTGCATTCGACTACATGGCCGTCACCTTGTCCGACCAAGGGGGTCAAAGCCTTGCAGGTCTTTTTGTTCAGGTTGTCGCTGAT<br>GTCATACGTGCTCCGGATCAAACGGTCCAGGCTGTCAATGTGATCAGCAAGGAAGACCTCCAGAGTCTGTGGACCTGGAATCGAACAGT<br>CCCGGAAACCGTACAGGCGTGTGTTCATGATATGATTGGACAAAAAGCCTGTGATAGCCCTGATTCCCCGGCAATCCATGCATGGGATGG<br>CGCGCTTACATATCAAGAGTTGGACCTTCTGTCCACCCGACTGGCCCGGTATCTTATAGATCTAGGGGTCCGGCAGAATACAGCCATCCCT<br>CTATGTTTCGAGAAGTCCATGTGGATGCCGGTAGCTGCACTGGCAGTGATGAAGACAGGCGCTGCATGCGTGGCCATGGATATGACCCA<br>GCCAGAGAAGCGCCTGCAGGCGATTGTGGACCTGGTGCAGCCGGATCTTCTCGTGACATCCGTCGCAAATCGCAAGACCGTCCAGCAA<br>CTGGCAGACACGAAGGCCGTGTTGGCGATTGACCAGGCATTCTTTTCCCAAATCGCACCTCCCACGTCGTCCTGCCTGCCGACAGTCTCC<br>CCATCAAGTCCTCTCTATACCGTCTTTACATCTGGCAGTACCGGCACACCCAAGGGAGCAGTCATCAGCCATGCCAACTTTGCCAGTGCCA<br>TTGTGCATCAAACGGACCTGCTCGCACTGGACCCCGATTCGCGCGTCTTTGACTTTGTCTCGTATGCCTTTGATGTTTCCTGGTCGAACCT<br>ACTCCACACGCTGGCCGCTGGGGCATGTTTGTGCATCCCCTCCGAAGCCATGCGCCGGGACAACCCTGTCGAGGCCATGCAGGCGATG<br>AAAGTGACCCATGCCCAATTTACCCCGTCGATGGCCCGTACAGTGGACCCGGATCAATGCAAGACGCTCAAGGCCTTGATCCTGGGCGG<br>CGAGGCCATGTCCCAGCACGACATCGCTGTCTGGGCACCTTGGGTTGACCTTCGTGTTGCGTATGGTCCCGCCGAGTGTACGGTTGCCG<br>CCGTCATGGACACAGTCCCTGAGCAGTCCGGACATCGGGACTTCGGCAAGATAGGGCGGGGGCTTGGTTCGAACACCTGGATTGTCAG<br>CGTATCGGATGGAGAGCGCCTGGCCCCGGTGGGCACTGTGGGAGAGCTGTGGCTGGAAGGGCCCCTGGTGGGCCTGGGCTATCTTGA	AATLSRFALIDVAASVVQGQLKFEFLYNREMK<br>GQDRILEWISKTESSLKAAAEELLLQSPSYTIC<br>DFPLLSLTDQGLDELLNRVLPAVGLSYGQIEDI<br>YPCAPIQEGIIMSQAKSPELYWTRVRWTVQS<br>TGTSPVDLDRLRRAWQMVVNRHSILRTIFID<br>GIGSGQVKDQVVLKDFPVDVEVLHAELHQL<br>AEPVTGRQWHTDLSSRRDTPQHSLVLTQTAS<br>GVVFCDLELNHAMVDAYSLALLRQEICAAYT<br>GSLPATPAPAYQAYIEHLQGLSLVEGQRFWQT<br>YLDNAQPCHFPALGQPDVTDGSNARRALSIS<br>LDAATHQALRVFCQQHAVTPSNVFYLAWGL<br>LLRAYTGLDTVCFGYLTSGRDVPVPGVDRIVG<br>PLINMLVCVLEFRTGASVRSAMQKVQEDYLA<br>ALQYQSTPLSKILQLSGTSGRGLFNTGISVQG<br>GATSGEPDEHDIIVTDQTGLDSPEYDIAVAISH<br>DEEETEIAFDYMAVTLSDQGGQSLAGLFVQV<br>VADVIRAPDQTVQAVNVISKEDLQSLWTWN<br>RTVPETVQACVHDMIGQKACDSPDSPAIHA<br>WDGALTYQELDLLSTRLARYLIDLGVRQNTAI<br>PLCFEKSMWMPVAALAVMKTGAACVAMD<br>MTQPEKRLQAIVDLVQPDLLVTSVANRKTVQ<br>QLADTKAVLAIDQAFFSQIAPPTSSCLPTVSPS<br>SPLYTVFTSGSTGTPKGAVISHANFASAIVHQT<br>DLLALDPDSRVFDFVSYAFDVSWSNLLHTLA<br>AGACLCIPSEAMRRDNPVEAMQAMKVTHA<br>QFTPSMARTVDPDQCKTLKALILGGEAMSQ<br>HDIAVWAPWVDLRVAYGPAECTVAAVMDT<br>VPEQSGHRDFGKIGRGLGSNTWIVSVSDGE<br>RLAPVGTVGELWLEGPLVGLGYLDQDEKTAA<br>SFIDNPAWLLRGGPGVPGRQGRLYRTGDLVR<br>YCFDGSILFLGRKDNQVKVRGQRVELQEVEH<br>HLQAHLSGTTGVVADVVKPQGSSNAMLVAY<br>LAVGETIHSPLDSVHAALRPLTQGLTDSLSARI<br>PQYMVPSMYLPVAEIPVTTTGKIDRKQLHEL<br>GSSLTMEQLAQIQPPQEGEQQAPQTDLEKLL<br>QQLWAELSAKLRSAGFRIAVPDIFKLKTISRLA<br>PSAASVQGRMKTTWETREDEPFELAPVQQ<br>MFVNVVRRKCNHFNQSFFLRITRQVRAEDV<br>RRALDLIVTQHPMLRARFASDQGGHWTQHI<br>KPYTPGCYRYCEHVESSLAEASPLLDASQVAL<br>DLESGPVFSGDLLQIRDSGDQYLYLVAHHLAV<br>DLVSWRIILADMEDHLTAKASSSFTPMPFQA

	TCAGGACGAAAAGACTGCCGCCAGTTTTATAGACAACCCTGCATGGCTCCTCCGAGGAGGTCCTGGTGTTCCTGGTCGTCAAGGCCGGT<br>TGTACCGAACTGGGGATCTTGTCCGCTACTGTTTTGATGGCAGTATTCTCTTCCTAGGACGCAAGGATAACCAGGTCAAAGTCCGCGGGC<br>AGCGTGTTGAATTGCAAGAAGTGGAGCATCATCTCCAGGCTCATCTGTCGGGAACAACCGGAGTCGTGGCCGACGTTGTCAAGCCCCA<br>GGGCAGCAGCAATGCCATGCTCGTCGCCTATCTCGCGGTTGGAGAGACTATCCATTCCCCTCTAGACAGCGTCCATGCAGCATTGCGGCC<br>CCTCACCCAAGGCCTCACGGACTCCCTCTCTGCCCGAATCCCGCAGTATATGGTCCCTAGCATGTACCTCCCCGTTGCAGAGATTCCAGTC<br>ACTACGACGGGAAAGATCGATCGAAAGCAGCTGCATGAATTAGGATCATCGCTTACAATGGAACAGTTAGCTCAAATCCAACCCCCCCAG<br>GAAGGCGAGCAGCAGGCTCCCCAGACAGACCTCGAGAAGCTCCTCCAGCAGCTCTGGGCAGAGGTCCTCAATATCAGTCAAGAACTCA<br>TTGGGATGCACGATAGTTTCTTTTCCCTCGGGGCGACTCAATCACAGCGATGCAGCTATCGGCCAAGCTGCGCTCAGCAGGCTTCCGTAT<br>CGCCGTTCCTGATATCTTCAAGCTGAAGACAATCTCCCGACTGGCTCCAAGTGCAGCATCTGTGCAGGGGCGGATGAAAACCACCTGGG<br>AGACCCGAGAAGACGAGCCATTCGAACTTGCTCCCGTTCAGCAGATGTTCGTCAACGTGGTGCGCCGCAAGTGCAATCATTTTAACCAG<br>AGTTTCTTCCTGCGCATCACCCGTCAGGTGCGGGCTGAGGATGTGCGGCGAGCCCTGGACTTGATTGTGACTCAACATCCTATGCTGAG<br>GGCACGGTTTGCCTCGGATCAGGGCGGACATTGGACGCAGCATATCAAGCCCTACACCCCCGGTTGCTACCGGTACTGCGAACACGTCG<br>AGTCCTCTCTGGCCGAGGCCTCGCCGCTGTTGGATGCCAGCCAAGTCGCCCTTGATCTGGAATCTGGACCGGTCTTCTCTGGGGATCTGC<br>TCCAGATTCGCGACAGCGGTGATCAATATCTGTACCTGGTGGCGCATCACTTGGCGGTCGATCTGGTATCCTGGAGAATCATTCTCGCGG<br>ACATGGAGGACCACCTGACAGCAAAAGCCTCTTCGTCTTTCACGCCGATGCCCTTCCAGGCATGGTGTCAACTTCAGGCCGACCATGCTC<br>GAGACTGCCTCACTCCCGAAACGGCGTTGCCCGTTGAGATTCCTCCTCCGCAGCAGGGTTACTGGGGAATTGATCCTGAACGCAACACC<br>TTTGGCAACGCCATCCACCGCAGCTTCACAGTGAGCAAGAACGTCACAGATATCCTCTTGGGGCCTGCCAATAAGTCATTTGATACCCAG<br>CCGGTCGAGATTCTACAGGGTGCTTTGATATACTCCTTCACTCAAGTGTTTCGGGATAGGGCGCCTCCTACAGTGTTCAGCGAAGGGCAT<br>GGGCGCGAGCCATGGGATGCCGCGATCGACCTATCAAGAACAGTTGGCTGGTTCACTACTATGCTTCCGACCGTAACCTCGATGGGACC<br>GGATGATACTCTACCTGATATCATCCGCCATACGAAAGATAACCGACGTCTAGTGCCAGGAAACGGATGGCCATACTTTGCATCCCGCTAC<br>TTGAACCCTGCTGGAAAGAAGGCATTTGGGGCCTACGGTCTGCCCGAGATCACATTCAACTACTTGGGACTCTACCAGCAGTTTGAAAA<br>AGACAGTTCCCTGCTTCAGCCAACCGCCTTGCCCTCAGGCAGCATGTCGGATGTTGACGAGCGGATGCAGCGCTTTGCCCTGATTGAGG<br>TTTCTGCATCGGTGTCCCAGGGCTGTCTGGAGTTTAGCTTCTTGTTCAGTCGTCATACAAAGCACTTGCGTAACCTTGAGGACTGGGTGG<br>CCCACTGTCAGCATGCTCTACGAACGGCTTCGGAAGTCTTGATGCAGCGCCAGCCAAGCTATACACGATGTGATTTCCCACTTATGCGCCT<br>GACAGAGCCAGCCTTGCAGACTCTCGTTGGATATAGTCTTCCACAGCTCGGAGTCTCGTATGGTCAAGTTGAGGATATCTACCCTTGCTC<br>GCCGATTCAGCACGGTATCTTGCTGAGCCAAGCGAAGAACCCTGGCGTGTATTGGACGCGCATTCGATGGAAGGCCCGGTCCTCCGAGT<br>CTGCATTGCCTCCCAATCCTGAACGACTAGCCCGTGCCTGGATGAGGGTCGTTGCACGACATCCCGTCCTTCGGACCCGGTTCATCGATG<br>GCCTTTCTCCCAACAGCGTGCAAGATCAGCTTGTCTTGAAAGCCAGCGAGCCCGAGATACATGTGATATCTGGTCCGGAGGCGACAGAT<br>CCCATCGCGGCTCTTGACTCTTACTGGGAGCAAAGACAGCGGAAAGGACACAAGCTTCACTCTTTCGTCCTCAGCCAGGCACCCTCCGG<br>CGAGGTCTTCTGTGACCTCGAAATGAACCATGCAATCACGGATGCCACTTCGACTGCTCTACTCAAGCGAGACCTTCAAGCCGCATACAC<br>TGAAACCCTCCCGACTATGCCGGGTCCTTCGTACAGCGATTACATCAGGCACATTCAAGCAATACCCGCGGAGGTGGGGATAGACTACTG<br>GCGACGGTATCTCGACGGCGTTCATCCCTGCATTTTCCCAACACTGGCAGCGAATGGCCAGGACGTGGGGGAGAATCCAAGAGGCTCC<br>ATACATCATATGCTCGATCAAGACACGAATACCAGTCTTCAAGTTTTTCTCAAGACGAACGAGCTGACCGCCTCCAACGTCTTTTACCTCG<br>CATGGGCCCTCATTCTCCGGTGCTTTGTTGGGTCGGAGACAGTGTGCTTTGGCTATCTAGTATCCGGTCGTGATGTCCCTATTGACAATGC<br>AGACAGGATAATCGGGCCGTTTATTAACATGCTGGTCAGCCGAGTTAGGCTAGGAGAGGGGGTCACTCTGATGGATATCATGAAGCAAA<br>GCCAGGCGGATTATCTGGACAGTCTCAAGCACCAGCATCGCTCTGTGGCGCAGGCCGCTCACTCTTCGGACGGGCCAGCCGAGCCTCTA<br>TTTAATAGCGTCATCTCGGTGCAAGGTATGGATCTGAAGAAGAGCAGCAAGAGTGAAGAACGTGGTCTCTGTCTTGAGGAGCAAGACG<br>GTAACGATCCAACAGAGGTACGTATTCCCATCCCTTACACATGCATTTGTGGACAGCTTTCCCCTAACCTGGTGATTTCCAGTATGATATCA<br>TGATGAATATTGGTCTGGGGGACCAGGAAACAGCAATGACGTTCAGCTATTACAAGTCCCTGTTATCGGAGCAACAGGCAAGCGGCATA<br>GTGGAGTCCCTGCTTCAAGCGGTCCGGGAAATCATCCACACTCCCTTCAAGAAGCCCTCCCAGGTGAATCTGTCAACGGGCCATGACCA<br>GCAGACTATCTGGAGCTGGAACGCCTCCGTCCCGCCCACAATCAACGTTCCAGTGCACGGTCTGATTGCCAACAAGGTGAAGGAGCAG<br>CCCGATGCCGACGCAATCTGCGCATGGGATGGCGAGTTGTCGTACGGGCAGCTAGATAGCTTATCAACATGCCTGGCCCATCATTTGGTG	WCQLQADHARDCLTPETALPVEIPPPQQGY<br>WGIDPERNTFGNAIHRSFTVSKNVTDILLGPA<br>NKSFDTQPVEILQGALIYSFTQVFRDRAPPTV<br>FSEGHGREPWDAAIDLSRTVGWFTTMLPTV<br>TSMGPDDTLPDIIRHTKDNRRLVPGNGWPY<br>FASRYLNPAGKKAFGAYGLPEITFNYLGLYQQ<br>FEKDSSLLQPTALPSGSMSDVDERMQRFALI<br>EVSASVSQGCLEFSFLFSRHTKHLRNLEDWV<br>AHCQHALRTASEVLMQRQPSYTRCDFPLMR<br>LTEPALQTLVGYSLPQLGVSYGQVEDIYPCSPI<br>QHGILLSQAKNPGVYWTRIRWKARSSESALP<br>PNPERLARAWMRVVARHPVLRTRFIDGLSP<br>NSVQDQLVLKASEPEIHVISGPEATDPIAALD<br>SYWEQRQRKGHKLHSFVLSQAPSGEVFCDL<br>EMNHAITDATSTALLKRDLQAAYTETLPTMP<br>GPSYSDYIRHIQAIPAEVGIDYWRRYLDGVHP<br>CIFPTLAANGQDVGENPRGSIHHMLDQDTN<br>TSLQVFLKTNELTASNVFYLAWALILRCFVGSE<br>TVCFGYLVSGRDVPIDNADRIIGPFINMLVSR<br>VRLGEGVTLMDIMKQSQADYLDSLKHQHRS<br>VAQAAHSSDGPAEPLFNSVISVQGMDLKKSS<br>KSEERGLCLEEQDGNDPTEYDIMMNIGLGD<br>QETAMTFSYYKSLLSEQQASGIVESLLQAVREI<br>IHTPFKKPSQVNLSTGHDQQTIWSWNASVP<br>PTINVPVHGLIANKVKEQPDADAICAWDGEL<br>SYGQLDSLSTCLAHHLVASGVCSDTVVPLCFE<br>KSMWMPVAMLAVMKAGGASVSMDASLPE<br>ERLRTIAKQTEPAVILCSEATSEKAGRLGAPRVI<br>TVGQRLLSGLTIPSRGTTLPKVDPSDRLYVTFT<br>SGSTGIPKGVMITHANMSSALVQQQEALSF<br>GPHVRVFDFVSYAWDVAWSNLLRTLVAGGC<br>LCVPSDSQRREGIEKAMTQLRINCVTLTPSVA<br>RLLNPAAVPHLNTLALIGEPLSQADIMRWAP<br>HTNEIINTYGPSECPACVTVNRIPLDLLYEPNL<br>GAASACNTWIVDPDNTDQLVPVGGIGELWL<br>EGPLIGLGYLGLPERTAESFVENPRWLRSGYA<br>GQLGRGGRLYRTGDLVRYTDDGALVYIGRKD<br>SQVKIRGQRVELGEIEYQIREGIARISSATGDP<br>TVVAGVITPRGSGSKMLVVYLGLGQMATGP<br>VDRIREALAGYTSGLDVYLSENLPHYMIPNAY<br>IPVSEIPMTVTGKTDRGRLARTGASYTLTELA<br>EMQPSRGRQRPPTTAMERRLQQLWAAVLG

	GCTTCTGGAGTCTGTTCGGACACGGTGGTTCCTCTCTGCTTTGAAAAATCCATGTGGATGCCGGTGGCCATGCTCGCTGTGATGAAAGCC<br>GGTGGTGCTTCAGTCTCCATGGACGCGAGCCTGCCAGAAGAACGATTGCGCACAATCGCAAAGCAAACGGAGCCCGCGGTGATTCTCT<br>GCTCGGAAGCAACCAGCGAGAAGGCGGGTCGACTTGGTGCCCCCCGAGTGATTACTGTCGGCCAAAGGTTGCTCTCAGGGCTGACGA<br>TCCCCAGTCGAGGCACGACGCTCCCCAAGGTCGATCCATCTGACCGATTATACGTGACTTTCACATCCGGCAGCACGGGGATTCCCAAGG<br>GAGTCATGATCACGCATGCAAACATGAGCAGCGCCCTGGTCCAGCAGCAGGAGGCCCTCTCATTCGGGCCTCACGTCCGCGTCTTTGAT<br>TTTGTGTCCTACGCGTGGGACGTGGCTTGGTCCAACCTGCTCCGTACTTTGGTTGCTGGTGGCTGTCTCTGTGTCCCATCTGACTCCCAGC<br>GCCGGGAAGGGATTGAAAAGGCCATGACACAATTAAGAATCAACTGTGTTACTCTCACACCCAGTGTCGCTCGTCTGCTCAACCCTGCT<br>GCGGTGCCGCACCTGAATACCCTGGCTCTAATTGGGGAGCCCCTGTCGCAGGCCGATATCATGCGGTGGGCTCCTCACACCAACGAGAT<br>CATCAACACCTACGGGCCATCCGAGTGTCCTGCATGCGTCACGGTGAATCGCATCCCGCTGGACCTCTTGTATGAGCCCAATCTCGGGGC<br>GGCTTCTGCCTGCAACACTTGGATTGTGGATCCCGATAACACCGATCAGCTGGTTCCAGTTGGTGGTATCGGAGAGCTCTGGCTGGAAG<br>GGCCCCTCATTGGGCTTGGATACCTGGGCCTCCCCGAACGAACGGCCGAGAGCTTTGTCGAGAATCCCCGCTGGCTGCGCTCCGGGTAT<br>GCTGGACAGCTGGGGCGTGGCGGCCGGCTGTATCGCACCGGCGACCTGGTCCGCTACACCGATGATGGCGCGCTTGTTTATATTGGACG<br>CAAGGACTCGCAGGTCAAGATCCGCGGCCAGCGCGTGGAGCTGGGAGAAATCGAGTACCAGATCCGCGAGGGCATCGCGCGCATCTC<br>GTCCGCCACCGGCGATCCCACCGTCGTGGCTGGGGTTATTACTCCGCGCGGAAGCGGCAGCAAGATGCTGGTCGTCTATCTTGGCCTAG<br>GGCAGATGGCCACAGGCCCTGTTGACCGCATTCGAGAGGCTCTGGCGGGCTATACAAGCGGGCTGGATGTGTATCTCTCGGAGAACCT<br>CCCCCACTACATGATCCCCAATGCATACATCCCCGTCTCTGAAATTCCAATGACAGTGACCGGCAAGACGGACCGCGGACGCCTGGCCCG<br>TACCGGGGCATCATACACCCTGACCGAACTGGCCGAGATGCAACCCTCGCGCGGCAGGCAGCGGCCCCCTACCACCGCTATGGAGCGG<br>CGGCTCCAACAGCTTTGGGCGGCAGTCCTGGGGCTCGATGTCAGTGCCATCGCTGCCGATGACAGTTTCTTCCGCATTGGCGGGGATTC<br>GATTGCGGCGATCCGCCTGGGCCAACGTGCGAGCGAGGATGGTCTGGCCGTGGCCGTGGCCGACATCTTCCGCAAGCCCCGTCTCTGC<br>GATCTGGCTCTCCTCGCTCGTGAGGACCAGGTCACCTACCTTGATCCTTCTCCCTTCTCCCTTCTCCCTGCCGGTTCTACCTCGGACTACGT<br>GACAGCTGGTCTCAGTCCGCTCCTGGACTGCCCACAGCACCACATCGCCGACGTCTATCCCACCACGGACCTCCAGAGCATCTACGCATC<br>CTTTGCAGTGAATGCACATCGGGGGGAGATCGAGTACATCTACATGGACCTTCCTCAGGGGGTGGACATCGCACGCGTCCGCCGCAGCT<br>GTCTGGATATGTGGCACCATCTCGACATCCTTCGCACCGTTTTCATTCTTGATCAAGGTCGTCTATTGCAAGTCGTGCTCAACAACGTGGA<br>GCCCGAGATTACCATGCAACACACCGAAGGGGATCTGGCAGCAGCATGCGAAGAAGCCTACAGCTGTGATCTACATGAGTCGCTGAGC<br>CTGGGCCGGTCGTTCACGCGGTTCTTCATCACCTCCACCGCAGACGGTCGAGTGCGATTCACCATCCGGCTCTCTCACGCTCAGTACGAC<br>GGCTACAGTCTGCCTGTCATCTTCTCCGTCTTTGCGGCCTTCTATGAGGGCCAGACACCACCCCCAGCCCCGAAGTTTTCGGGCTATATCC<br>GCCACATGCAAAAGCAGCAAGAGGCCGCGTATCCCTACTGGCGCACGCTCTTGCAAGGCTCGTCCATCACCCGGACCAGACACCTCTCC<br>ATAGCCGATGGCCACTGTCTGCCAAACCAGCCCCGTGGCCGGCTGGTTCAATCCAAGACCGCTGTGCCTGCCCCGTCAGATCAACCGGG<br>ATTCACGTCGGCAACGGTCTTTACCACCCTCTGTGCTCGCATGCTGGCACAGATGACAGGCGTCAGCGACGTGGTCTTTGGGACCATTGT<br>CTCCGGCCGTTCCTCCTTACCAATCGCCCTCCAAAACGTCGCCGGGCCATGTGTCAACACCATCCCCGTCCGCGTGCAGATCGAACCCGA<br>CCAGCCACTAGAGGAGCAGCTGGCCAGCGTCCACGACCAACACATCCAGGGCATGTCTTTTGAGACGAGCCAGTTCAGCGACATCGCC<br>GCCCACAGCACCACTTGGCCCGAGGACTCGCGCGCTCCCGACCTGGTTGTTCAGTTCCAGAACCTCGACAACCTGGAGCATGACAAGG<br>GTACGGAAATCCAGGGGATAGGCAGTACACTTGCACCGTGGGAGCAGCGGAACCGGTCCGTGGACAGCGATTTCCTCTTTATTCTGGC<br>GAAGCCGGTGGAAGAAGCCTGGGAGATATCGGTGTCGGCGAGCTCTAAGGTATACACCCAGGATACTCTGGATGCCATGCTCAAGGCCT<br>TGTGTTTGCATGTTGAGAATGTTTAA	LDVSAIAADDSFFRIGGDSIAAIRLGQRASED<br>GLAVAVADIFRKPRLCDLALLAREDQVTYLDP<br>SPFSLLPAGSTSDYVTAGLSPLLDCPQHHIAD<br>VYPTTDLQSIYASFAVNAHRGEIEYIYMDLPQ<br>GVDIARVRRSCLDMWHHLDILRTVFILDQGR<br>LLQVVLNNVEPEITMQHTEGDLAAACEEAYS<br>CDLHESLSLGRSFTRFFITSTADGRVRFTIRLSH<br>AQYDGYSLPVIFSVFAAFYEGQTPPPAPKFSG<br>YIRHMQKQQEAAYPYWRTLLQGSSITRTRHL<br>SIADGHCLPNQPRGRLVQSKTAVPAPSDQPG<br>FTSATVFTTLCARMLAQMTGVSDVVFGTIVS<br>GRSSLPIALQNVAGPCVNTIPVRVQIEPDQPL<br>EEQLASVHDQHIQGMSFETSQFSDIAAHSTT<br>WPEDSRAPDLVVQFQNLDNLEHDKGTEIQG<br>IGSTLAPWEQRNRSVDSDFLFILAKPVEEAW<br>EISVSASSKVYTQDTLDAMLKALCLHVENV
ungB’’	ATGTACCCAGCCAAGTGGGTGTTCCTGATCGGGGTGGCGATATTCGAGATCGGATCTCTGGTCTGCGGAGTGTCTCCCAGCTCGGGTGC<br>CCTGATTGCGGGCCGCAGCATCTCAGGACTGGGCGCTGGCAGCATCAACGCCGGCGCTGTCGTGATCGTGACCAACACCATTCCTGTGC<br>GAAAGCGGCCGATCTACCTGGGCTGTCTGGGGTGTGTCCATGGGCTAGTCAGCGTGTCGGGGCCATTGTGAGCTCATCTCCGTGCAACC<br>ATAATCCACATAGATCGGGGGCTAATGCCGTGTGACGAACAGGATTGGGGGTTTGCTGACGGACCATGCCTCGTGGCGATGGTGTTTCT<br>ATCTCAATCTGCCCATCGGAGCAATCACCATCCTGGGCATCATTTTCTTTCTGTCGGCTAACCAACCCCCGTCGGGACATCTCAGCTGGAG<br>GGAAAAACTTCAGTCCATGGATCTTCTGGGCTCGGCGTTCTTTATTCCTGGGATCGTCACGCTTCTGCTAGCCCTGGAATGGGGAGGGTC<br>ACAGTATGCCTGGGACAATTGGAGGATCATCCTTCTCTTCATCCTGTCCGCCATCTTGCTGATCGTCTTTGCCGGCGTTCAGATTCGCGCC	MYPAKWVFLIGVAIFEIGSLVCGVSPSSGALIA<br>GRSISGLGAGSINAGAVVIVTNTIPVRKRPIYL<br>GCLGIGGLLTDHASWRWCFYLNLPIGAITILGI<br>IFFLSANQPPSGHLSWREKLQSMDLLGSAFFI<br>PGIVTLLLALEWGGSQYAWDNWRIILLFILSAI<br>LLIVFAGVQIRAPQGKATLPPRLVSNRNILGLI<br>GYIVGNSGGLFVFVYYLPIWLQAIKGFSASKS

	CCTCAGGGTAAAGCGACACTCCCCCCTCGCCTGGTTTCAAATCGGAATATCCTGGGTCTTATCGGCTATATTGTTGGTAACAGTGGGGGG<br>TTGTTTGTCTTTGTGTACTATGTATGTTCTCGACCTGGATCCCCCCCCCCCCCCCCTTTTCCCTAGCTGGAATCTCCGCAACATGGCTTAACG<br>ACTTCGTCACCTTGACATAGCTGCCAATTTGGCTCCAAGCCATCAAGGGGTTCTCGGCCTCCAAGTCAGGTCTTTCAATCTTACCCACCCA<br>GTTAGGGGTGGTGATTGCATCTCTAGCTGGCGGGGTCTTAGTCACTTTCGTCGGATACTACACGCCCTTTCTGATTATATCCTCCCTAATGG<br>CCGTCGCTGGCGCCGGCATCCTGAGCTCCCTGCATCCGTCCTCCAGCCTCGCAAGCATCCTGGGGTACCAAGTGGTGCTGAGTCTGGGC<br>ATCGGGCTTGGATCGCAGAACGCCATGGTCGTGCCGTCCGTGGTTTGCGCCCCGGACGATGTAGTCACGGCCATTGCGATGCTCTGCTT<br>CCTGCAGATGCTGTCCAGCTCGGTCGCTCTGTCCATTGGACAGACAGTTTTCCACAACCGGCTGGTGGCCAATTTGCATCGCAGCGCCCC<br>GTCCGTGGACCCCTCCCTCGTTGAAGAAGGGGCGACGCTGCTCCGCGATCGCGTTCCATCGGAGCTTTTGCCGTCTGTTCTTGGGGCGT<br>ATAGCAAGGCTGTCTCGCAGACCTTTTACGTCGGGGTGGCCATGTGTGCGCTGTCGCTTCTTGGGTCGGCCTCCATGCAGTGGAAGCGA<br>GTCCCTGGCAAGAAAGATGCGGCAGAGGAGGAGAAGGAGGAAGAGTCGCGCGACAGTGATACCCCTGCACCACCTGCGACAGAAGA<br>GAAGACGTGTAAGCAATAGAAGGGCGTGGGTGTATACCTTGCATCCCCCATGTGTGTTCTGA	GLSILPTQLGVVIASLAGGVLVTFVGYYTPFLII<br>SSLMAVAGAGILSSLHPSSSLASILGYQVVLSL<br>GIGLGSQNAMVVPSVVCAPDDVVTAIAMLC<br>FLQMLSSSVALSIGQTVFHNRLVANLHRSAPS<br>VDPSLVEEGATLLRDRVPSELLPSVLGAYSKAV<br>SQTFYVGVAMCALSLLGSASMQWKRVPGK<br>KDAAEEEKEEESRDSDTPAPPATEEKTCKQ
ungC’’	ATGACAGTGACAGTACCGCAGGATCTCCGGCTGGGAGAGCCATATGGTGATCGTGCAAACAGTCTGTTGGTCCAAATCCCAACATGGGC<br>GGACATGGTCGATTTTGGGCAGGGAAAGGCGCAGATGAACCACGGCTATCCACGGTCCATTGTGCACCCTGATATCCGTCTGGTAAGGC<br>CCTTGGCTTTCTTTGATCTCGTTCAGCTGGGGCCACCCGGATTGGCCCTGATACTGACGATGATGATGTGTTTGTCCTTCAAGCTCAGTTC<br>CGCAATTCTGGCCAAGGTCCGCGACGACAAAGAATGCACCGATGCTTCCCTTCTATTGTTCTCTGGTGCCCGGACGGCGCTTTCCTGCAA<br>ACAGTATATCCTCCATCTCGCGCAGGCCACGGATCGGCCAACCCTCGCCGATTCGATCCGGGTGTACGTGGTGGACTTCAGTGGGTTCTT<br>GTGTCGTGACCATCACGCTGCTCACTCGTCTGGATCAACCCTTTATGCCGTCCTCTACCCCAGTGCGGCTTGTCGGGATGCTCATGCGTTT<br>TGGCAACGCGCGGGGCCAGGGATATCATCAAGGTTGGCACAGCGATGCCTGCGGTCCAGTTCTCGACCTCGGATCCACCCATTCACTGC<br>TCCTACCACCGCCACCGCCCCTCCCGACACCCATTCTGTGTACGAGAAACTCCGTTGTAGAATTGCTGGCCATGTGAGACACTGGTCGAG<br>TGATCCTCGGATCACCAAGCAGGTCGCCCCCTCCGATGTCTTCTTATATGCTTCCGGCATGGCAGCCATCTACCACGTGCATCAGTCCATTC<br>TGGCCTGGCGAACCGGGGAGTCCGTGAATGCCGGCCTCCTGTATGGGCCGACCCTCAGTATCCTGCAAGCGGAAGGTCAGGGGTTAAG<br>GTCCTACAACCTAGGAACGGAGGCCGACTTGAATCATCTTGCTGCCCAGCTGGAGCTGGGATCCGGGGAAGCTCGCGCGGTGCAAGCA<br>ATCTGGTGTGAATGTCCCAGTAACCTCACACTGCAGACGGTCAATTTGCAGCGGATACGTCGGCTGGCGGACCAACATGGCATTCCTGTA<br>GTAGTGGACGACAGCATTGCCAGCTTCGCCAACTTGGATCTGTTGGGAGTGGCGGACATTATCGTCTCCTCGCTGTCCAAATACTTCAGC<br>GGATATGCCGACGTAATGGCAGGAAGGCAAGTCAAGTCAAGTCAAGTCAAAGACCCAGGCGCCAACCTACAGACACGGTGACTGATAC<br>TCTCCTCTGCCGGCATACAGCGTCATTCTGAATCCGAATTCTCCACACTACGCCGCCCTGCACAAGCAGATATCCGCCACATACGAGAACA<br>ACCTCTTCGTCGAAGATGCGATCCGACTAGAATCCAACAGCCGGAACTTCCTCCCTCGCATGGATCGAGTCAACAAGACAACGCAACAC<br>CTGGTCACCCAGCTTTTGCCTCTCGTCTCGGATCCCGCGAGCCCATTGACCCGCATTTTCCACCCGTCCGTGTGCGCATCCCGTCCGAACT<br>ATGAGCGTCAGATGCGTGCTCCCTCGTCTGAGATGCCTCGCCCGGGGTATGGAGGCGTCTTCACGATGGAGTTCGCCGACATCGCAAGC<br>GCGTCCGTCTTCTTTGACCACTTACACGTCTGCAAGGGCCTCTCTTTTGGCGCGGATGTCTGCATCGCGTCGCCGTATATGCAGATGACG<br>GGGCAGGCCGGGAAGAAGCAGGCGTTGGTCAATGGCACGAATGATACTATCATCCGGTTTGCCGTGGGGTTGGAGGAGCCTGATGAG<br>GTCCTGCACCGCATCAACACCGCGCTAGACGCTGCGACGCTGGTGTATAGGGCTAAGGGCCATGTGGCCCCGTAG	MTVTVPQDLRLGEPYGDRANSLLVQIPTWA<br>DMVDFGQGKAQMNHGYPRSIVHPDIRLVR<br>PLAFFDLVQLGPPGLALILTMMMCLSFKLSSA<br>ILAKVRDDKECTDASLLLFSGARTALSCKQYIL<br>HLAQATDRPTLADSIRVYVVDFSGFLCRDHH<br>AAHSSGSTLYAVLYPSAACRDAHAFWQRAGP<br>GISSRLAQRCLRSSSRPRIHPFTAPTTATAPPD<br>THSVYEKLRCRIAGHVRHWSSDPRITKQ<br>VAPSDVFLYASGMAAIYHVHQSILAWRTGES<br>VNAGLLYGPTLSILQAEGQGLRSYNLGTEADL<br>NHLAAQLELGSGEARAVQAIWCECPSNLTLQ<br>TVNLQRIRRLADQHGIPVVVDDSIASFANLDL<br>LGVADIIVSSLSKYFSGYADISATYENNLFVED<br>AIRLESNSRNFLPRMDRVNKTTQHLVTQLLPL<br>VSDPASPLTRIFHPSVCASRPNYERQMRAPSS<br>EMPRPGYGGVFTMEFADIASASVFFDHLHV<br>CKGLSFGADVCIASPYM<br>QMTGQAGKKQALVNGTNDTIIRFAVGLEEP<br>DEVLHRINTALDAATLVYRAKGHVAP
ungD’’	ATGGCCATGCTGGTCGAGGACAATGACAAGTACCCCGGAGTGCAATGGACTACGCCCGTGAATGAGTTGATCCGCGAGGACTTCGTTCT<br>GGACAACCACTGGACGACGGCAAACGTCACCATTGAGGATATCCTGTCCCATCGGACCGGAATGCCGGGCCATAACTTCTCGCTTGGAG<br>CTGTGTATGCTGACAAGCAGGCCACTGTTCAAGATGTGGTGAGGAGCTTGCGCTTCCTCCCGTCTACCGCACCCCCACGCACGACCTACC<br>AGTACAATAACGCCATGTATATCGTGGCGTCGCATCTTATCCAAACAGTCATGGGCGATGACCTCGGCTCTATCTTTCAAAAGCACATCTGG<br>GACCCTCTCGGGATGAGCAGCACGTATTTCCGCCTGGATGATGCACTTGCCAGTCAAAAGCCCTTGGCAAAGGGCTATGCCTTCGCCGA<br>GGGCAAGTATGAAGCGGTGGAGTGGAAGAACAGGCCGGAAATCTCCGGCGCCGGTGCCATTATCAGCACTGTGGAAGACTACGCGAA<br>ATGGATCTACGCACTACTGAATCAGAGCGGGTTGCCTCTTTCCTCGGAGGGCTACGGAACACTCTGGACCGCGAGAGCACTCATTCCAA<br>ATTCCGAGCCGTTTTTGGCCCCGATGGCGTATGCCCTTGGATGGGACCGTTACATCTACCAGGGAGTGGAGATCATCACTCACGATGGGG<br>GGATAGAGGGATTCGGAGCCGAGATTGTGATGATCCCCGCGCTTAAATTTGGGGTCATCACCATGGCTAACTCGACCTATTCGTCCAACT<br>ATGGGGGAACATGCCTGGCGTATGAGCTTATCGATTCGAAGCTGGGCATCGCTGCGGGGGACAGATTTGACTGGAAACAAAAGTGTGT	MAMLVEDNDKYPGVQWTTPVNELIREDFVL<br>DNHWTTANVTIEDILSHRTGMPGHNFSLGA<br>VYADKQATVQDVVRSLRFLPSTAPPRTTYQY<br>NNAMYIVASHLIQTVMGDDLGSIFQKHIWD<br>PLGMSSTYFRLDDALASQKPLAKGYAFAEGK<br>YEAVEWKNRPEISGAGAIISTVEDYAKWIYAL<br>LNQSGLPLSSEGYGTLWTARALIPNSEPFLAP<br>MAYALGWDRYIYQGVEIITHDGGIEGFGAEIV<br>MIPALKFGVITMANSTYSSNYGGTCLAYELID<br>SKLGIAAGDRFDWKQKYVDIVDQMDAYNA

	CTTGTCATTCCTCTTGCTCTCCAACAAACTGACTAAATCGGGAGCAGATATGTCGATATCGTCGACCAAATGGACGCTTACAATGCAGACG<br>CGGTCCAAATCTTCTACCCCGATCTCCCGTCTCCTCCTCTACCGGGGCCGACTCTCGCTATCGAAGCATACTCCGGCACCTACTGGCATGAT<br>GCCTACGGTCGGCTCGATCTCTCCATAGATGGCACCGGCACAAAGCTCCACGCGGACCGAACTAATTGTACCAATACCTGCTCTCTTACAT<br>TCGAGAACGTCACGGGAAACTACTTTATTATTAGGCTGCTTGTAGTTGGAGCCGAGACCGTCCTTCCAGCTGAGTTTTCGGTTGACCCGG<br>ACGGGAAGCCAAGGTCTGTCGGTATAGGGTGGGAGCCAACGATGGGGATAGAGAAGAAGATTTGGATGCGCAAGGTCGATGGTGATG<br>ATACCGTTGGGCTGCATCGCGCCCTGCCGTACAAGCCATCCAGAACTCCCCAGCTGCCGGAGTTTTTGACTGGCCACCTTTTTGTCTAG	DAVQIFYPDLPSPPLPGPTLAIEAYSGTYWHD<br>AYGRLDLSIDGTGTKLHADRTNCTNTCSLTFE<br>NVTGNYFIIRLLVVGAETVLPAEFSVDPDGKP<br>RSVGIGWEPTMGIEKKIWMRKVDGDDTVG<br>LHRALPYKPSRTPQLPEFLTGHLFV

	UngA A1	UngA A2	UngA A3	UngA A4	UngA A5	UngA A6	UngA A7	UngA' A1	UngA' A2	UngA' A3	UngA' A4	UngA' A5	UngA' A6	UngA' A7	UngA'' A1	UngA'' A2	UngA'' A3	UngA'' A4	UngA'' A5	UngA'' A6	UngA'' A7
UngA A1		50.495%	47.146%	49.754%	54.455%	52.580%	53.606%	69.479%	47.794%	48.000%	50.000%	53.234%	51.870%	52.882%	79.259%	49.754%	44.444%	51.351%	52.723%	53.846%	53.580%
UngA A2	50.495%		52.228%	53.056%	47.420%	47.922%	49.880%	48.267%	58.231%	52.120%	53.695%	45.658%	45.771%	44.750%	50.000%	77.037%	52.840%	55.392%	46.552%	47.277%	49.140%
UngA A3	47.146%	52.228%		49.140%	47.277%	46.437%	46.154%	50.990%	50.739%	55.473%	49.261%	48.139%	45.771%	45.750%	48.395%	52.956%	74.938%	50.245%	45.926%	45.926%	47.912%
UngA A4	49.754%	53.056%	49.140%		49.010%	50.735%	47.255%	49.507%	49.633%	50.123%	64.532%	49.877%	50.743%	49.005%	49.877%	55.012%	49.140%	81.373%	48.157%	49.507%	47.677%
UngA A5	54.455%	47.420%	47.277%	49.010%		48.522%	48.441%	52.228%	46.305%	47.015%	48.020%	56.188%	48.507%	49.626%	52.970%	48.522%	43.704%	50.370%	75.802%	48.883%	50.493%
UngA A6	52.580%	47.922%	46.437%	50.735%	48.522%		48.931%	49.140%	46.455%	47.525%	51.843%	46.798%	61.139%	49.380%	50.614%	46.569%	46.192%	52.696%	49.877%	73.449%	49.389%
UngA A7	53.606%	49.880%	46.154%	47.255%	48.441%	48.931%		52.174%	48.086%	46.359%	48.798%	48.910%	48.786%	65.281%	51.202%	48.558%	46.747%	50.600%	50.602%	48.193%	77.831%
UngA' A1	69.479%	48.267%	50.990%	49.507%	52.228%	49.140%	52.174%		46.552%	50.126%	51.741%	54.135%	53.133%	50.126%	70.896%	48.515%	45.906%	51.980%	52.239%	50.746%	53.598%
UngA' A2	47.794%	58.231%	50.739%	49.633%	46.305%	46.455%	48.086%	46.552%		50.000%	49.140%	48.395%	44.665%	45.025%	48.030%	57.740%	51.478%	50.611%	45.679%	46.914%	47.420%
UngA' A3	48.000%	52.120%	55.473%	50.123%	47.015%	47.525%	46.359%	50.126%	50.000%		49.000%	48.485%	46.970%	45.431%	47.870%	55.250%	55.779%	51.122%	47.250%	46.883%	48.130%
UngA' A4	50.000%	53.695%	49.261%	64.532%	48.020%	51.843%	48.798%	51.741%	49.140%	49.000%		47.132%	52.500%	49.246%	50.000%	54.074%	47.772%	66.832%	49.257%	50.746%	49.136%
UngA' A5	53.234%	45.658%	48.139%	49.877%	56.188%	46.798%	48.910%	54.135%	48.395%	48.485%	47.132%		49.507%	49.136%	52.120%	46.020%	46.650%	51.117%	57.214%	49.000%	50.873%
UngA' A6	51.870%	45.771%	45.771%	50.743%	48.507%	61.139%	48.786%	53.133%	44.665%	46.970%	52.500%	49.507%		49.877%	50.627%	47.500%	45.636%	53.367%	48.371%	62.720%	50.503%
UngA' A7	52.882%	44.750%	45.750%	49.005%	49.626%	49.380%	65.281%	50.126%	45.025%	45.431%	49.246%	49.136%	49.877%		51.378%	47.000%	45.729%	51.500%	52.513%	50.882%	68.514%
UngA'' A1	79.259%	50.000%	48.395%	49.877%	52.970%	50.614%	51.202%	70.896%	48.030%	47.870%	50.000%	52.120%	50.627%	51.378%		48.889%	45.297%	51.111%	53.483%	52.854%	51.980%
UngA'' A2	49.754%	77.037%	52.956%	55.012%	48.522%	46.569%	48.558%	48.515%	57.740%	55.250%	54.074%	46.020%	47.500%	47.000%	48.889%		53.086%	57.389%	47.395%	47.382%	50.124%
UngA'' A3	44.444%	52.840%	74.938%	49.140%	43.704%	46.192%	46.747%	45.906%	51.478%	55.779%	47.772%	46.650%	45.636%	45.729%	45.297%	53.086%		49.507%	44.776%	45.522%	47.891%
UngA'' A4	51.351%	55.392%	50.245%	81.373%	50.370%	52.696%	50.600%	51.980%	50.611%	51.122%	66.832%	51.117%	53.367%	51.500%	51.111%	57.389%	49.507%		51.117%	52.120%	50.743%
UngA'' A5	52.723%	46.552%	45.926%	48.157%	75.802%	49.877%	50.602%	52.239%	45.679%	47.250%	49.257%	57.214%	48.371%	52.513%	53.483%	47.395%	44.776%	51.117%		51.000%	52.239%
UngA'' A6	53.846%	47.277%	45.926%	49.507%	48.883%	73.449%	48.193%	50.746%	46.914%	46.883%	50.746%	49.000%	62.720%	50.882%	52.854%	47.382%	45.522%	52.120%	51.000%		48.878%
UngA'' A7	53.580%	49.140%	47.912%	47.677%	50.493%	49.389%	77.831%	53.598%	47.420%	48.130%	49.136%	50.873%	50.503%	68.514%	51.980%	50.124%	47.891%	50.743%	52.239%	48.878%	

Figure S1: A domain sequence alignment comparing UngA, UngA', and UngA''.

	UngA C1	UngA C2	UngA C3	UngA C4	UngA C5	UngA C6	UngA C7	UngA' C1	UngA' C2	UngA' C3	UngA' C4	UngA' C5	UngA' C6	UngA' C7	UngA'' C1	UngA'' C2	UngA'' C3	UngA'' C4	UngA'' C5	UngA'' C6	UngA'' C7
UngA C1		25.606%	49.091%	24.390%	28.014%	24.000%	25.172%	58.824%	20.557%	47.601%	25.347%	25.725%	22.662%	24.747%	74.074%	25.685%	49.638%	25.000%	26.897%	25.510%	23.875%
UngA C2	25.606%		24.232%	41.924%	41.812%	45.033%	19.388%	23.958%	49.826%	22.759%	43.151%	39.493%	43.369%	19.289%	23.183%	70.205%	25.597%	41.638%	46.048%	45.734%	22.680%
UngA C3	49.091%	24.232%		21.993%	25.263%	22.772%	25.256%	49.455%	20.962%	60.219%	22.680%	25.271%	23.132%	25.248%	48.727%	27.703%	71.739%	24.315%	25.939%	24.832%	25.000%
UngA C4	24.390%	41.924%	21.993%		44.170%	40.864%	19.932%	23.958%	45.139%	19.723%	54.167%	44.565%	42.652%	20.305%	24.042%	42.466%	25.086%	67.596%	43.643%	41.837%	20.000%
UngA C5	28.014%	41.812%	25.263%	44.170%		43.434%	19.723%	25.442%	43.945%	24.735%	46.831%	51.852%	43.750%	17.617%	26.950%	44.251%	27.368%	46.127%	70.139%	45.329%	21.107%
UngA C6	24.000%	45.033%	22.772%	40.864%	43.434%		20.266%	26.000%	46.488%	22.591%	43.854%	39.649%	50.347%	17.647%	20.667%	47.020%	24.422%	39.867%	44.333%	71.854%	20.598%
UngA C7	25.172%	19.388%	25.256%	19.932%	19.723%	20.266%		21.453%	20.690%	22.759%	20.678%	18.705%	19.573%	56.701%	24.138%	18.644%	24.232%	19.932%	22.337%	21.356%	70.070%
UngA' C1	58.824%	23.958%	49.455%	23.958%	25.442%	26.000%	21.453%		23.077%	49.815%	25.436%	22.022%	24.731%	21.212%	60.662%	25.086%	46.739%	23.875%	25.862%	25.939%	22.300%
UngA' C2	20.557%	49.826%	20.962%	45.139%	43.945%	46.488%	20.690%	23.077%		20.833%	45.675%	42.125%	44.928%	18.229%	20.209%	55.363%	24.742%	44.483%	43.836%	46.528%	21.799%
UngA' C3	47.601%	22.759%	60.219%	19.723%	24.735%	22.591%	22.759%	49.815%	20.833%		22.569%	24.727%	21.223%	23.116%	50.554%	23.891%	57.818%	20.690%	24.742%	24.407%	23.183%
UngA' C4	25.347%	43.151%	22.680%	54.167%	46.831%	43.854%	20.678%	25.436%	45.675%	22.569%		45.652%	43.165%	22.727%	22.917%	44.863%	24.055%	56.491%	45.890%	44.898%	20.408%
UngA' C5	25.725%	39.493%	25.271%	44.565%	51.852%	39.649%	18.705%	22.022%	42.125%	24.727%	45.652%		40.264%	16.461%	23.551%	41.516%	27.798%	48.913%	59.124%	41.727%	19.424%
UngA' C6	22.662%	43.369%	23.132%	42.652%	43.750%	50.347%	19.573%	24.731%	44.928%	21.223%	43.165%	40.264%		18.584%	20.504%	49.104%	23.843%	42.652%	44.765%	53.381%	19.929%
UngA' C7	24.747%	19.289%	25.248%	20.305%	17.617%	17.647%	56.701%	21.212%	18.229%	23.116%	22.727%	16.461%	18.584%		24.242%	17.347%	24.257%	21.212%	16.923%	23.350%	60.622%
UngA'' C1	74.074%	23.183%	48.727%	24.042%	26.950%	20.667%	24.138%	60.662%	20.209%	50.554%	22.917%	23.551%	20.504%	24.242%		23.288%	49.638%	24.306%	25.172%	23.129%	25.260%
UngA'' C2	25.685%	70.205%	27.703%	42.466%	44.251%	47.020%	18.644%	25.086%	55.363%	23.891%	44.863%	41.516%	49.104%	17.347%	23.288%		26.689%	44.027%	48.288%	47.959%	21.429%
UngA'' C3	49.638%	25.597%	71.739%	25.086%	27.368%	24.422%	24.232%	46.739%	24.742%	57.818%	24.055%	27.798%	23.843%	24.257%	49.638%	26.689%		26.370%	27.986%	25.839%	23.288%
UngA'' C4	25.000%	41.638%	24.315%	67.596%	46.127%	39.867%	19.932%	23.875%	44.483%	20.690%	56.491%	48.913%	42.652%	21.212%	24.306%	44.027%	26.370%		45.890%	41.356%	21.017%
UngA'' C5	26.897%	46.048%	25.939%	43.643%	70.139%	44.333%	22.337%	25.862%	43.836%	24.742%	45.890%	59.124%	44.765%	16.923%	25.172%	48.288%	27.986%	45.890%		45.051%	24.399%
UngA'' C6	25.510%	45.734%	24.832%	41.837%	45.329%	71.854%	21.356%	25.939%	46.528%	24.407%	44.898%	41.727%	53.381%	23.350%	23.129%	47.959%	25.839%	41.356%	45.051%		22.449%
UngA'' C7	23.875%	22.680%	25.000%	20.000%	21.107%	20.598%	70.070%	22.300%	21.799%	23.183%	20.408%	19.424%	19.929%	60.622%	25.260%	21.429%	23.288%	21.017%	24.399%	22.449%	

Figure S2: C domain sequence alignment comparing UngA, UngA', and UngA''.

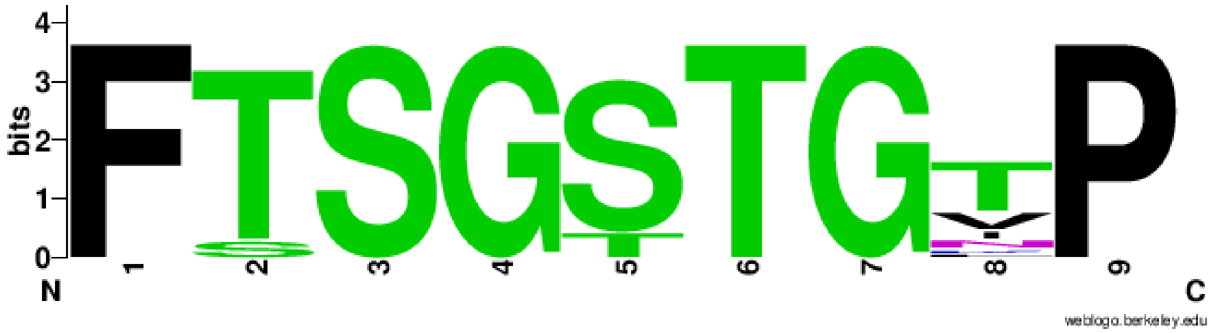

Figure S3: A domain AMP binding motif identified in extracted A domains.

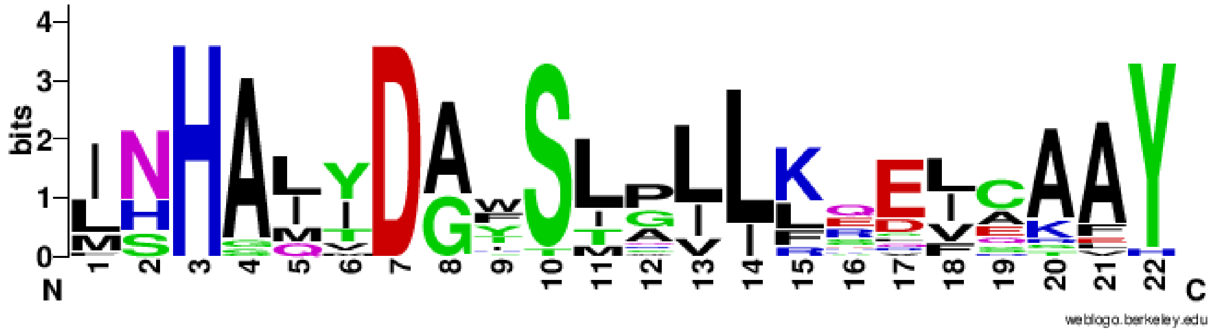

Figure S4: C domain active site identified in extracted C domains.

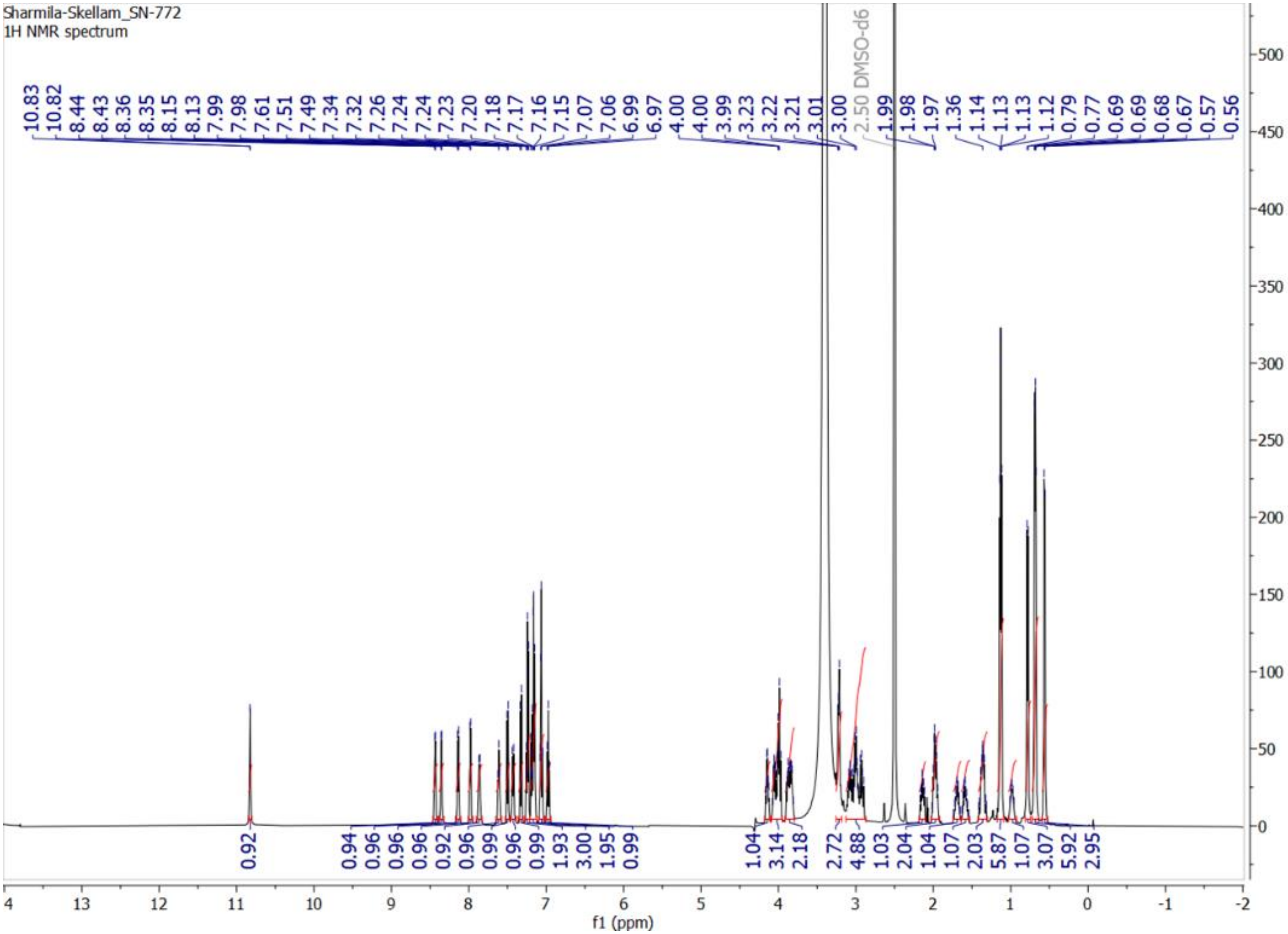

Figure S5: ^{1}H NMR spectrum of **1** (500 MHz, DMSO-d_6).

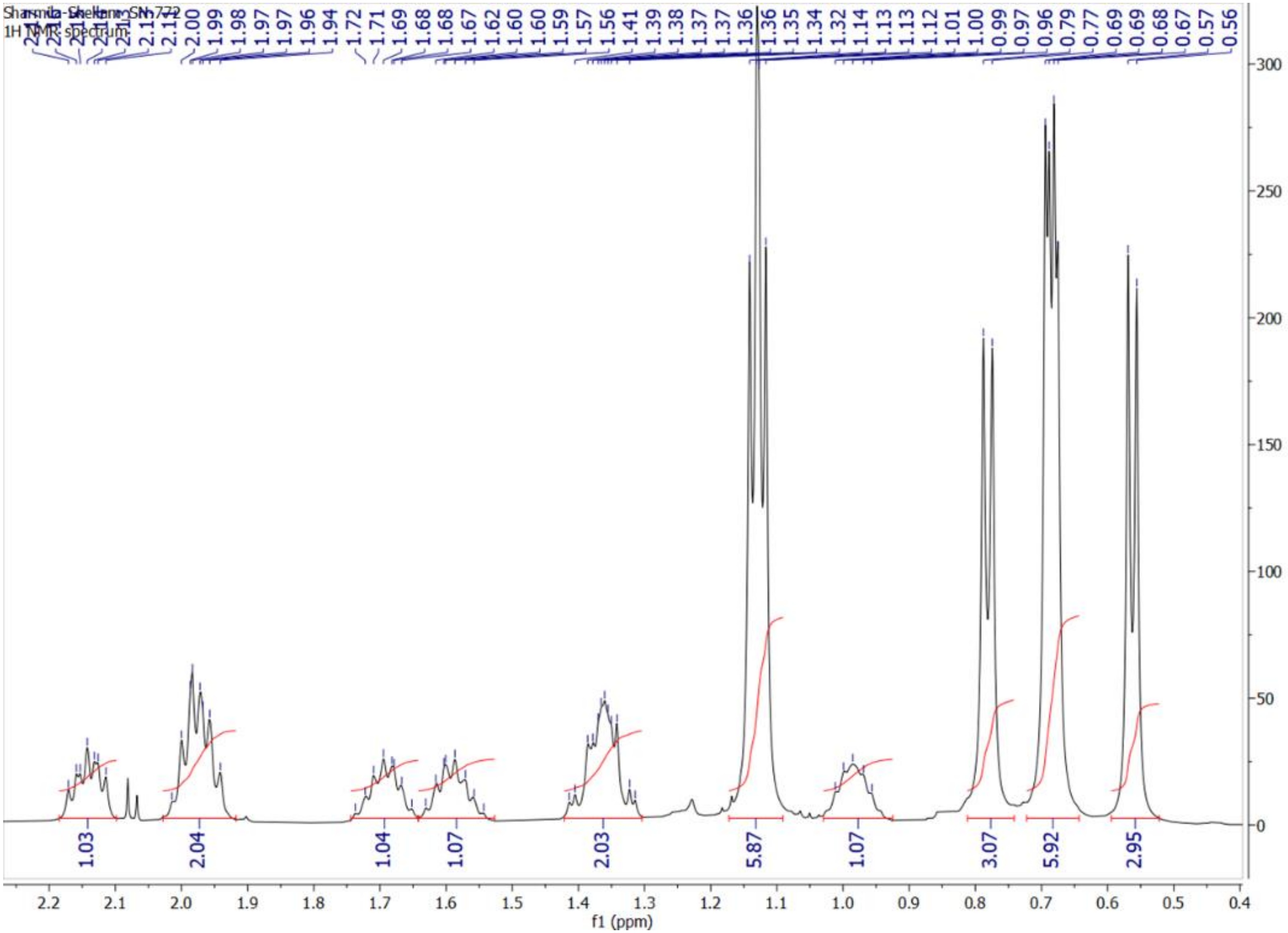

Figure S6: Expansion of the ^{1}H NMR spectrum of **1** (500 MHz, DMSO-d_6).

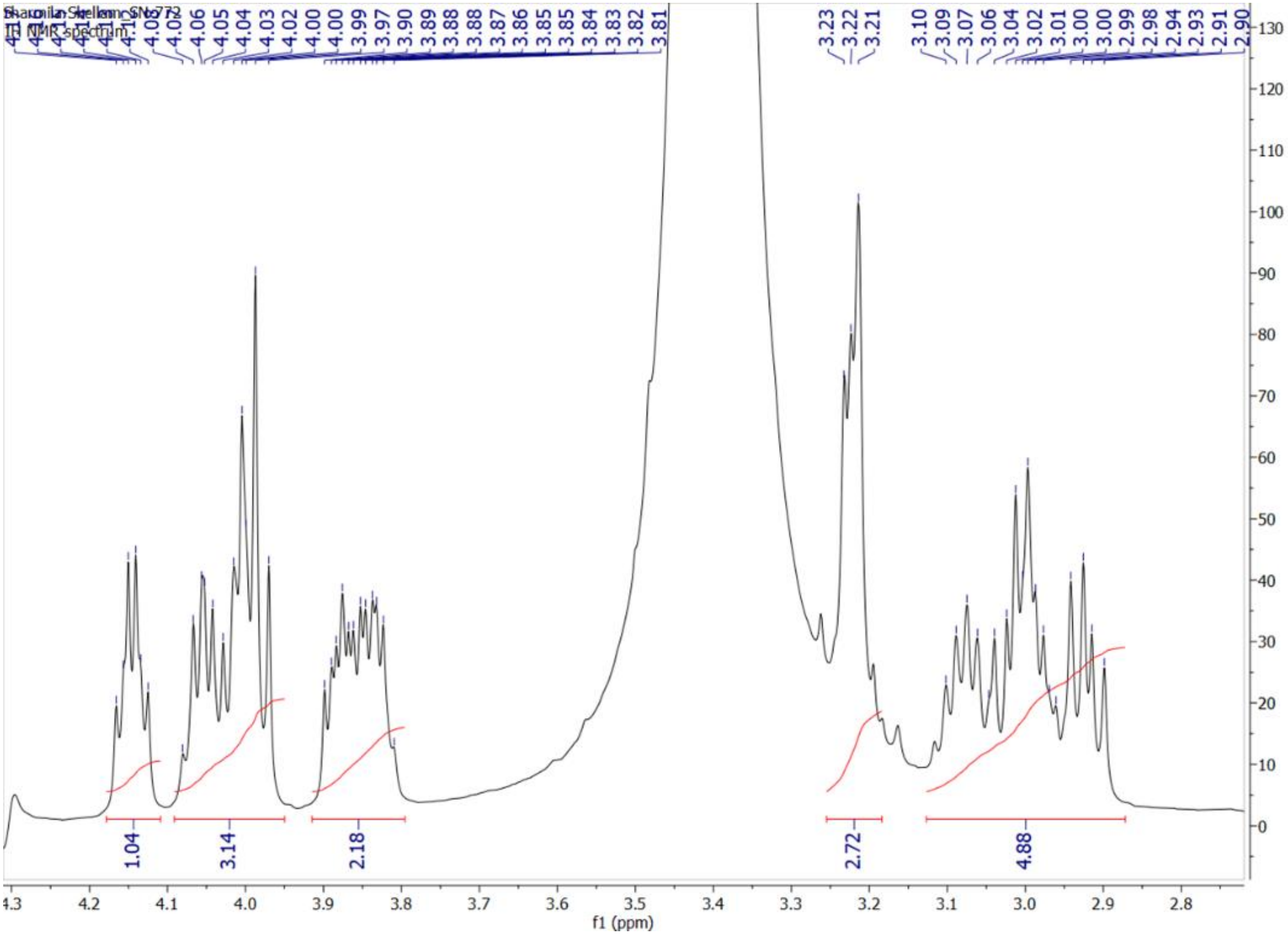

Figure S7: Expansion of the ^{1}H NMR spectrum of **1** (500 MHz, DMSO-d_6).

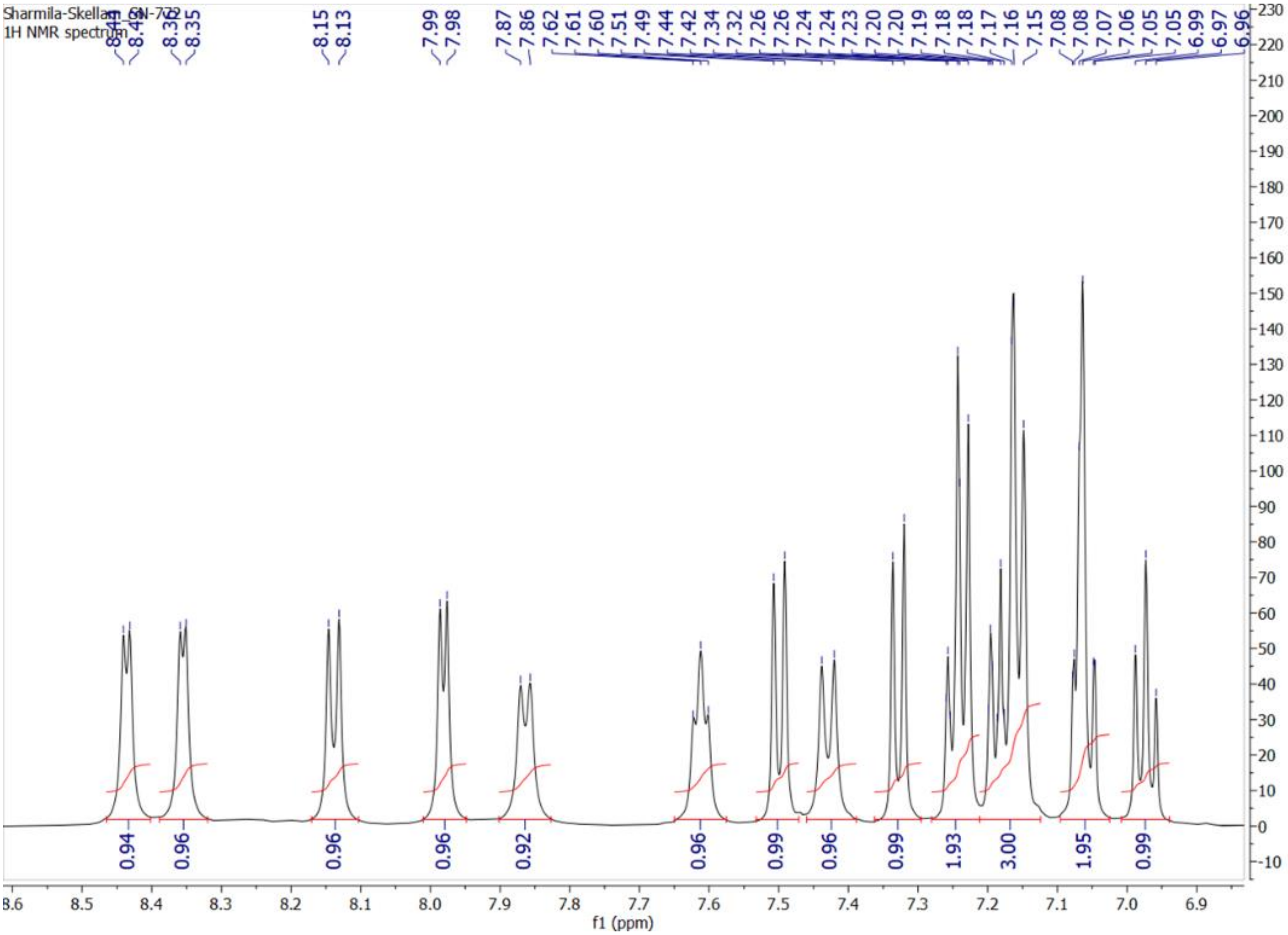

Figure S8: Expansion of the ^{1}H NMR spectrum of **1** (500 MHz, DMSO-d_6).

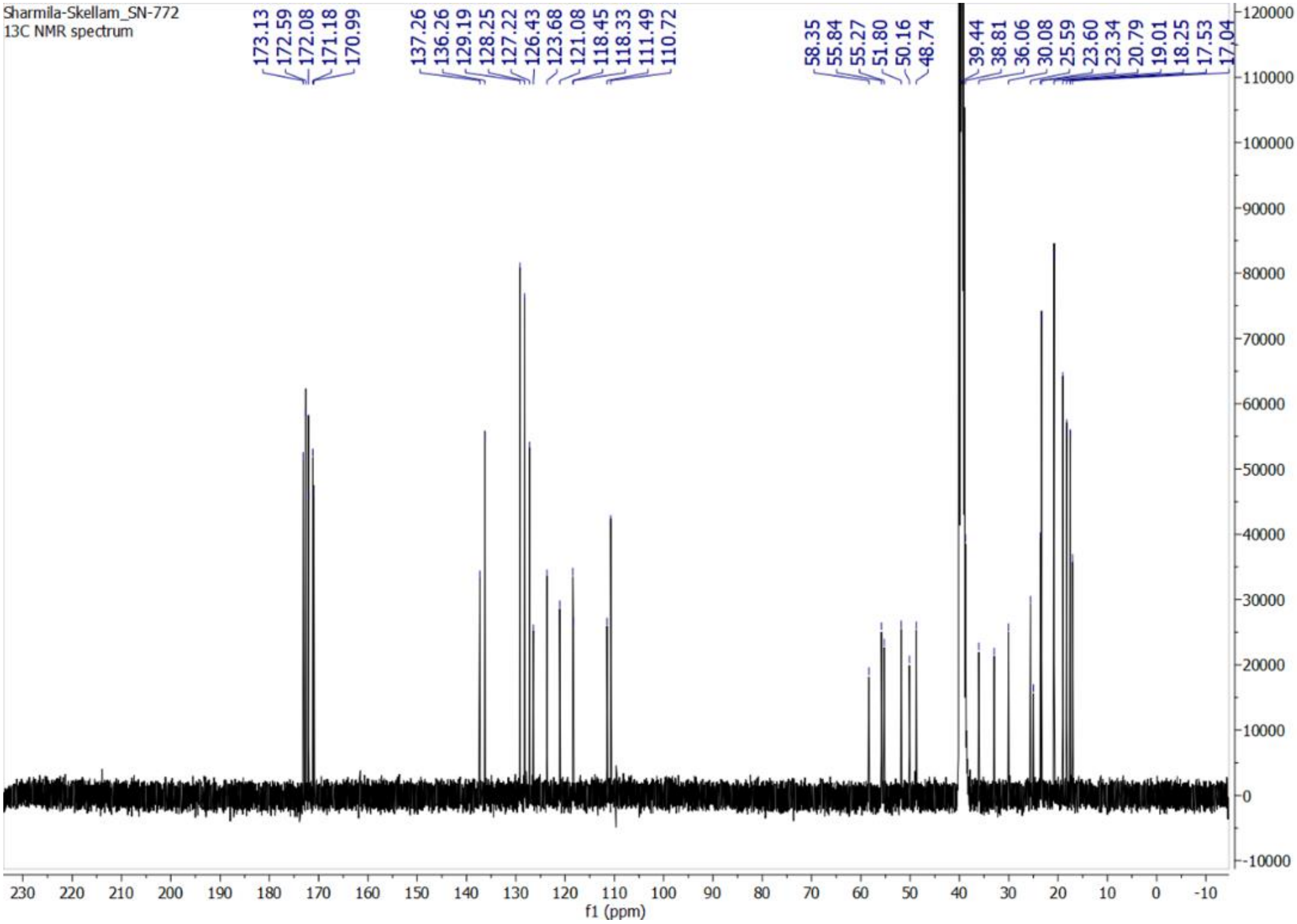

Figure S9: ^{13}C NMR spectrum of **1** (125 MHz, DMSO-d_6).

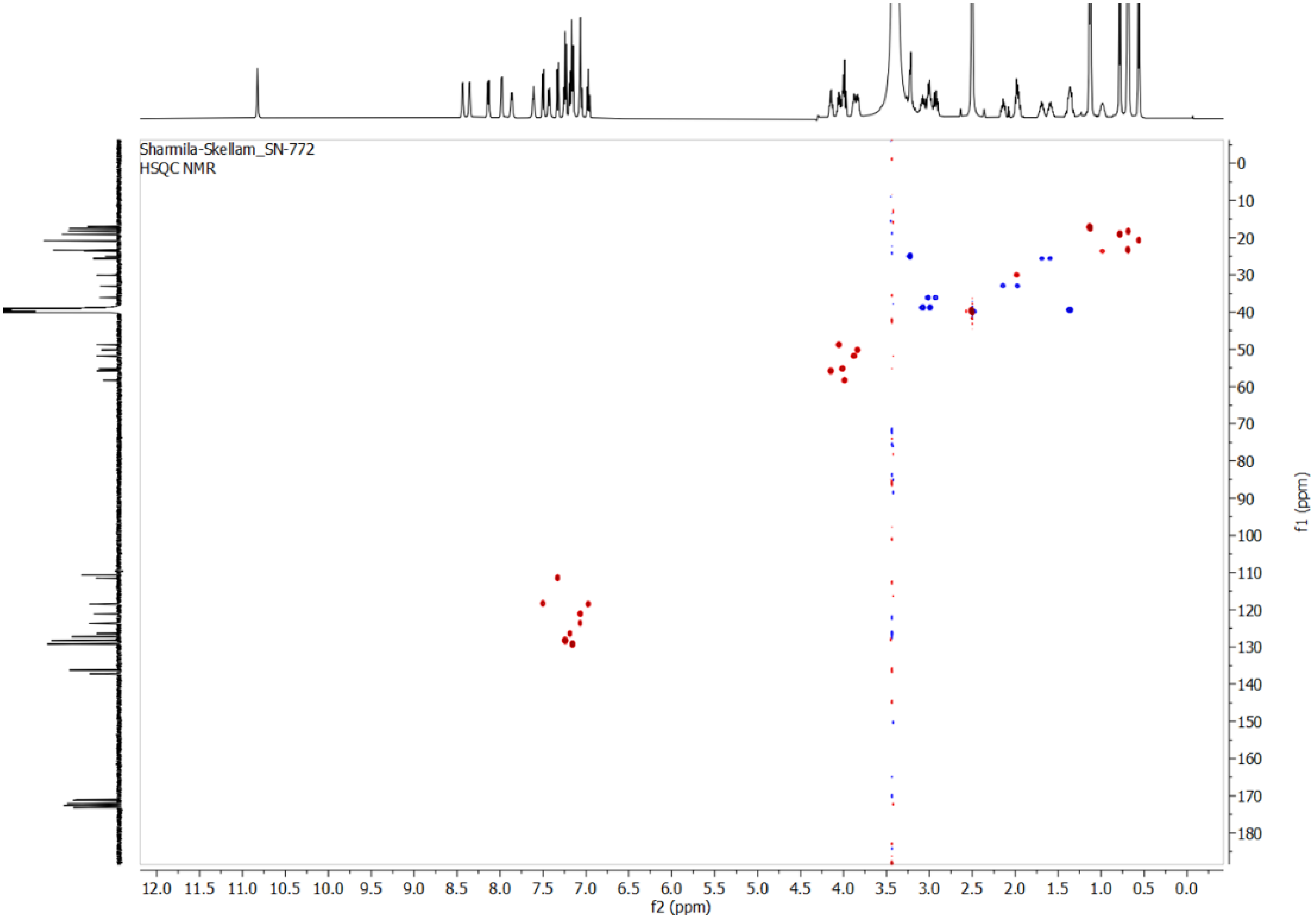

Figure S10: *g*HSQC spectrum of **1** (^{1}H:500 MHz; ^{13}C:125 MHz, DMSO-d_6).

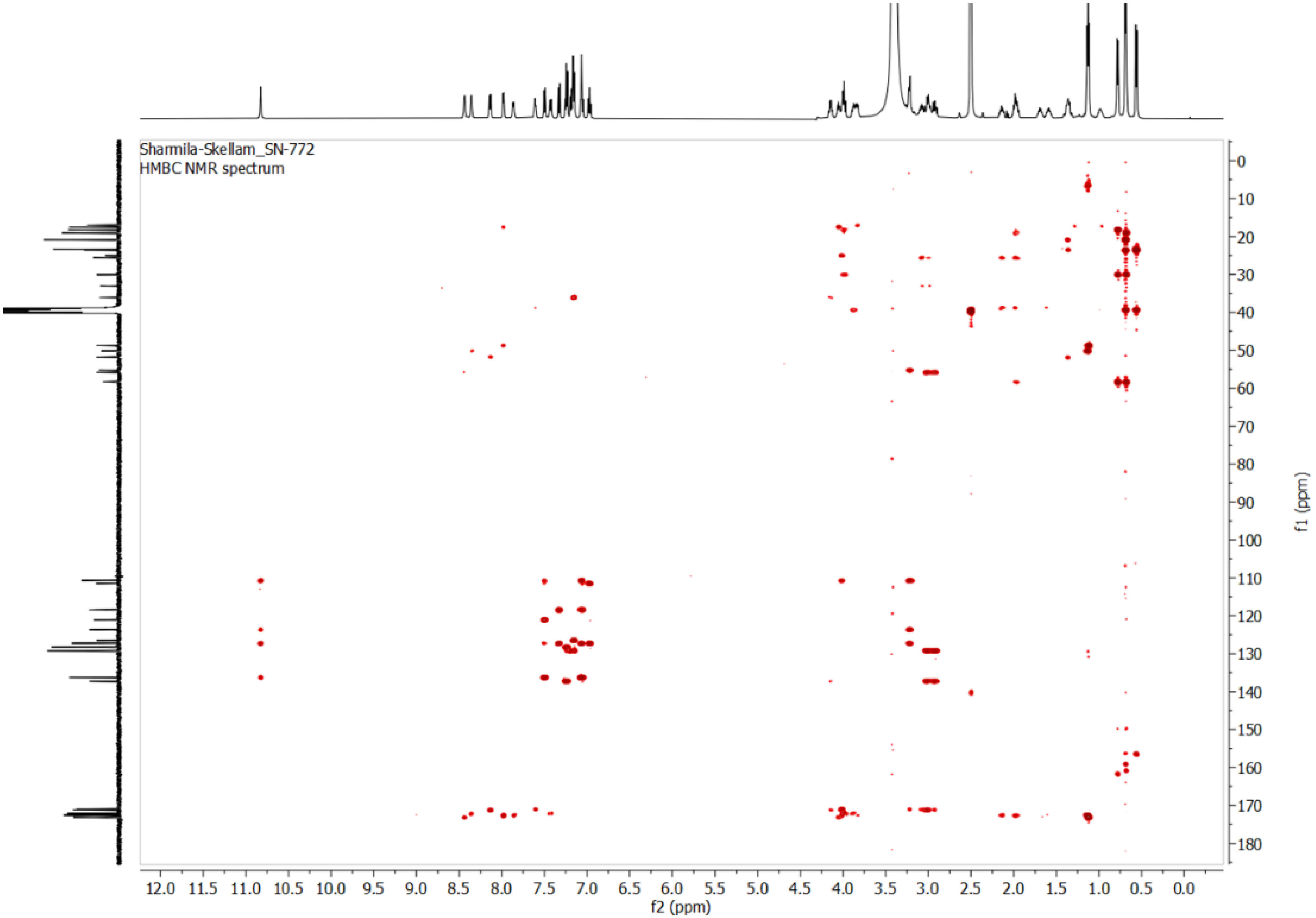

Figure S11: *g*HMBC spectrum of **1** (^{1}H:500 MHz; ^{13}C:125 MHz, DMSO-d_6).

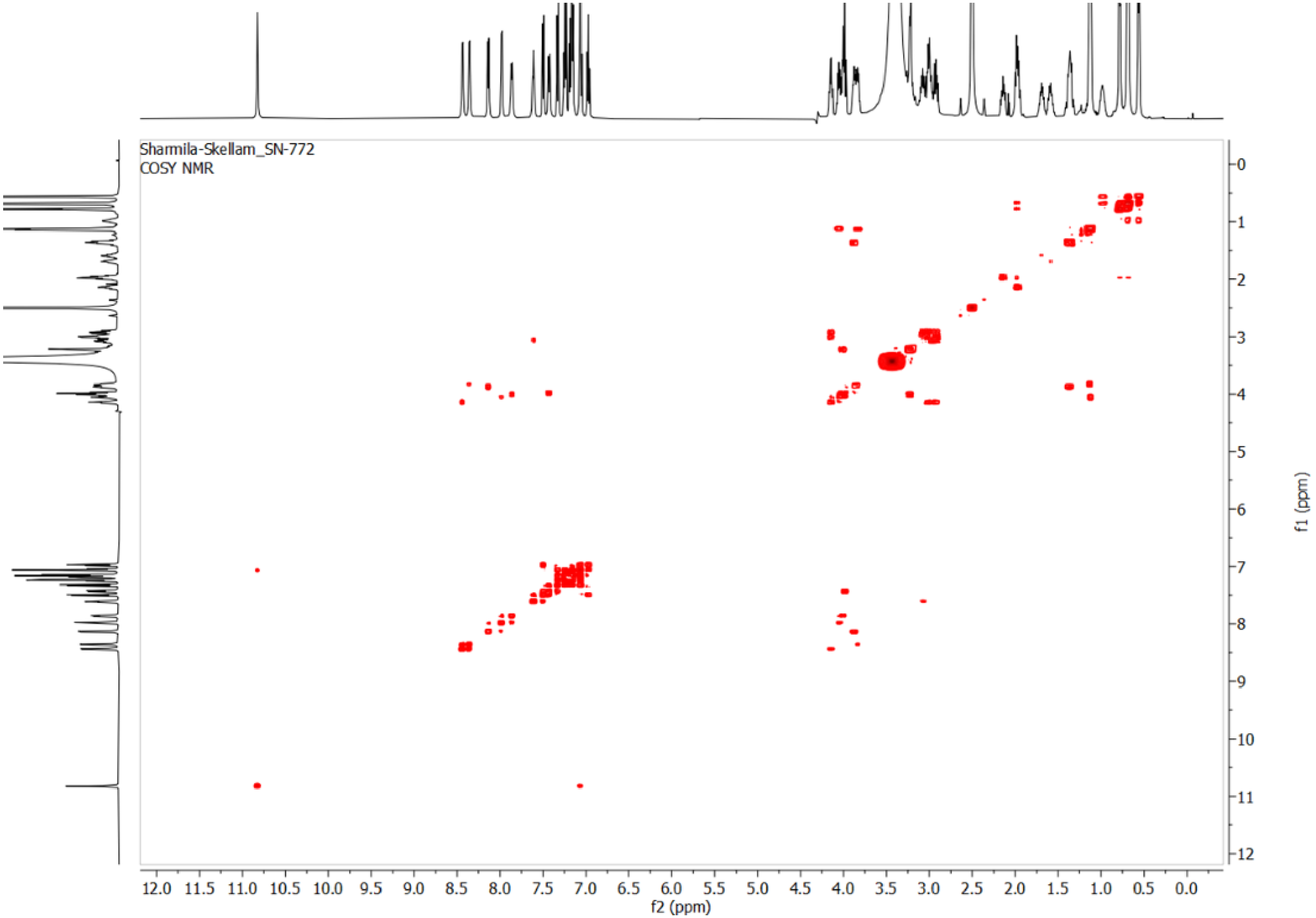

Figure S12: *g*COSY spectrum of **1** (500 MHz, DMSO-d_6).

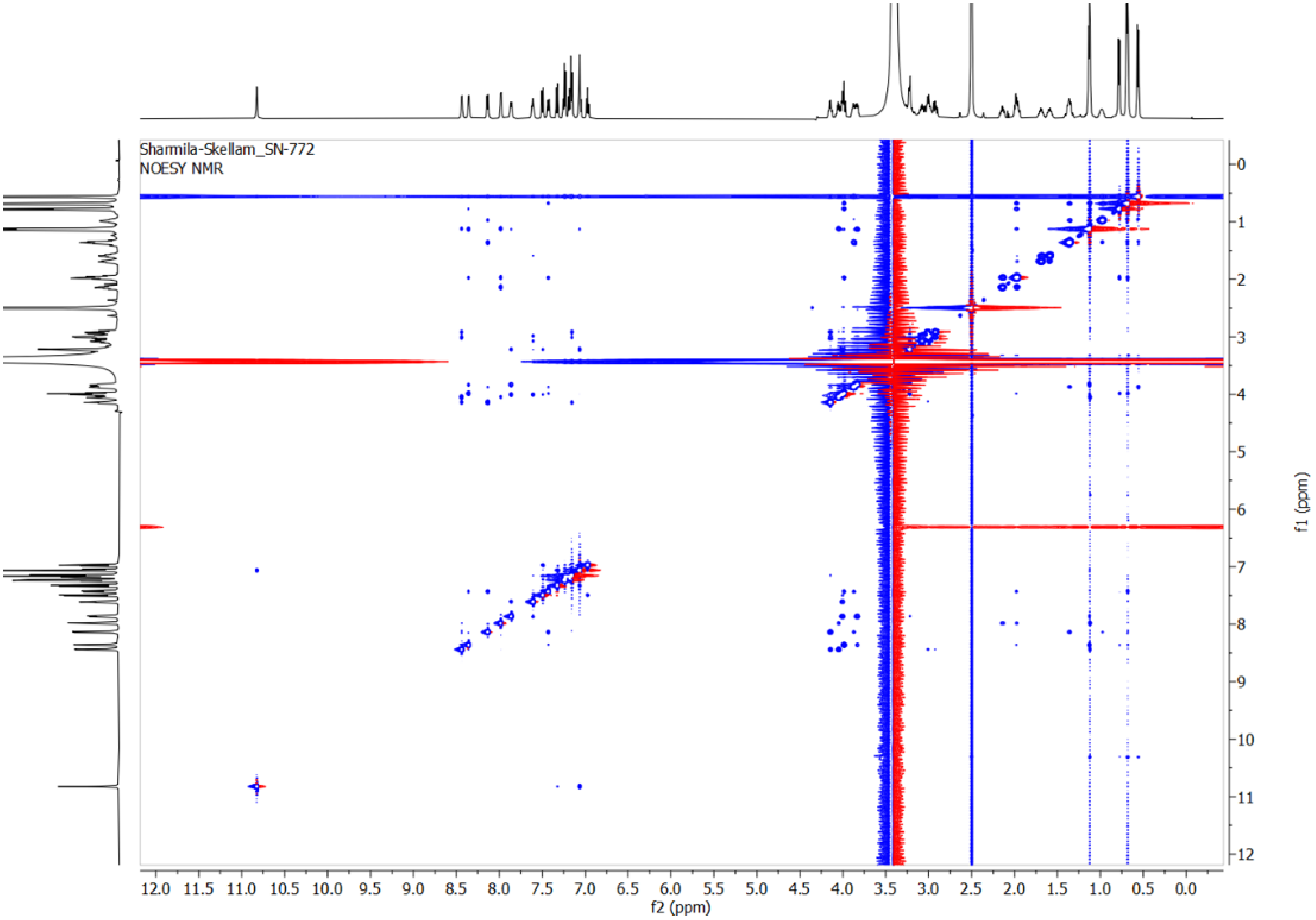

Figure S13: 2D-NOESY spectrum of **1** (500 MHz, DMSO-*d*$_6$).

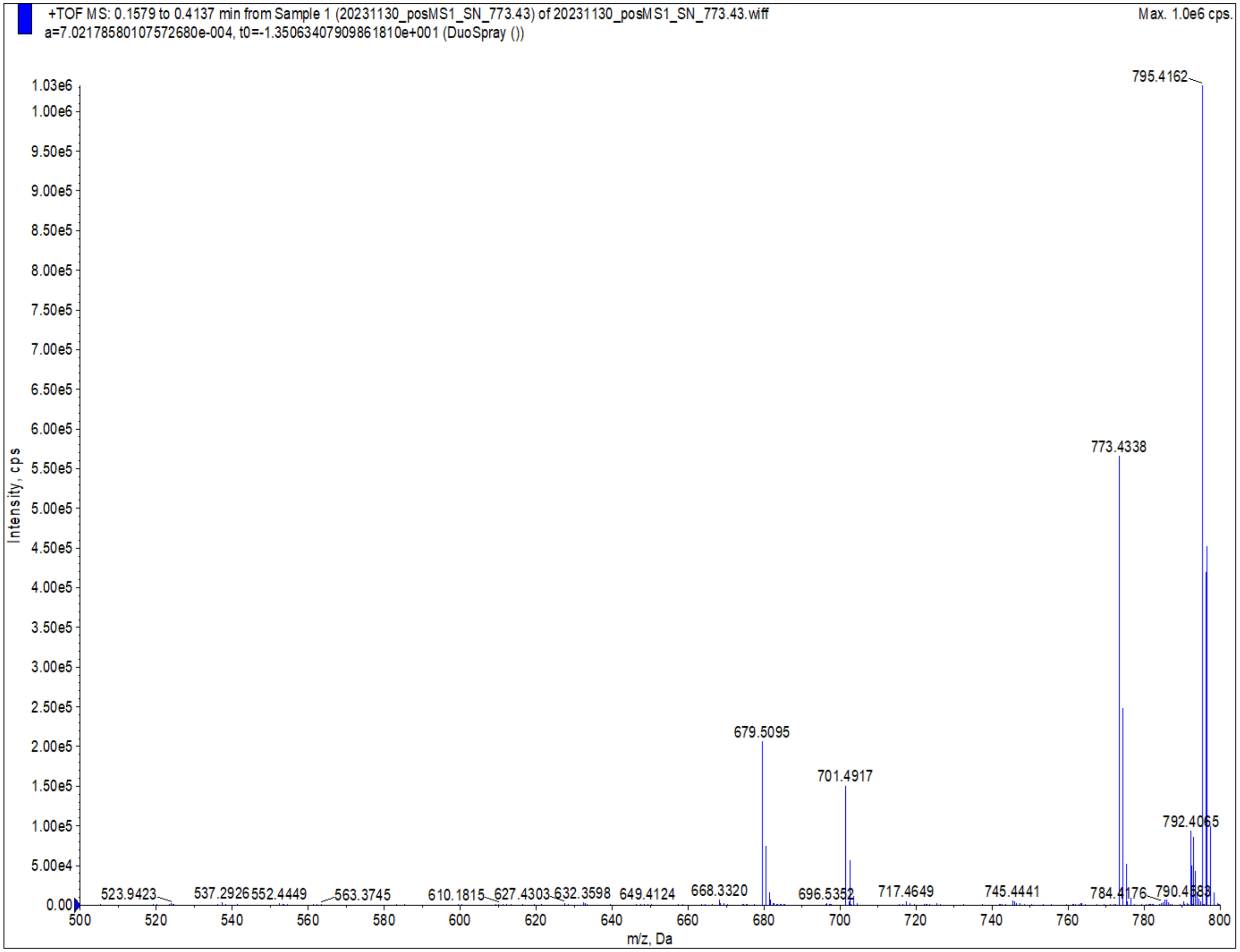

Figure S14: HRESIMS spectrum of **1** in positive ionization mode.

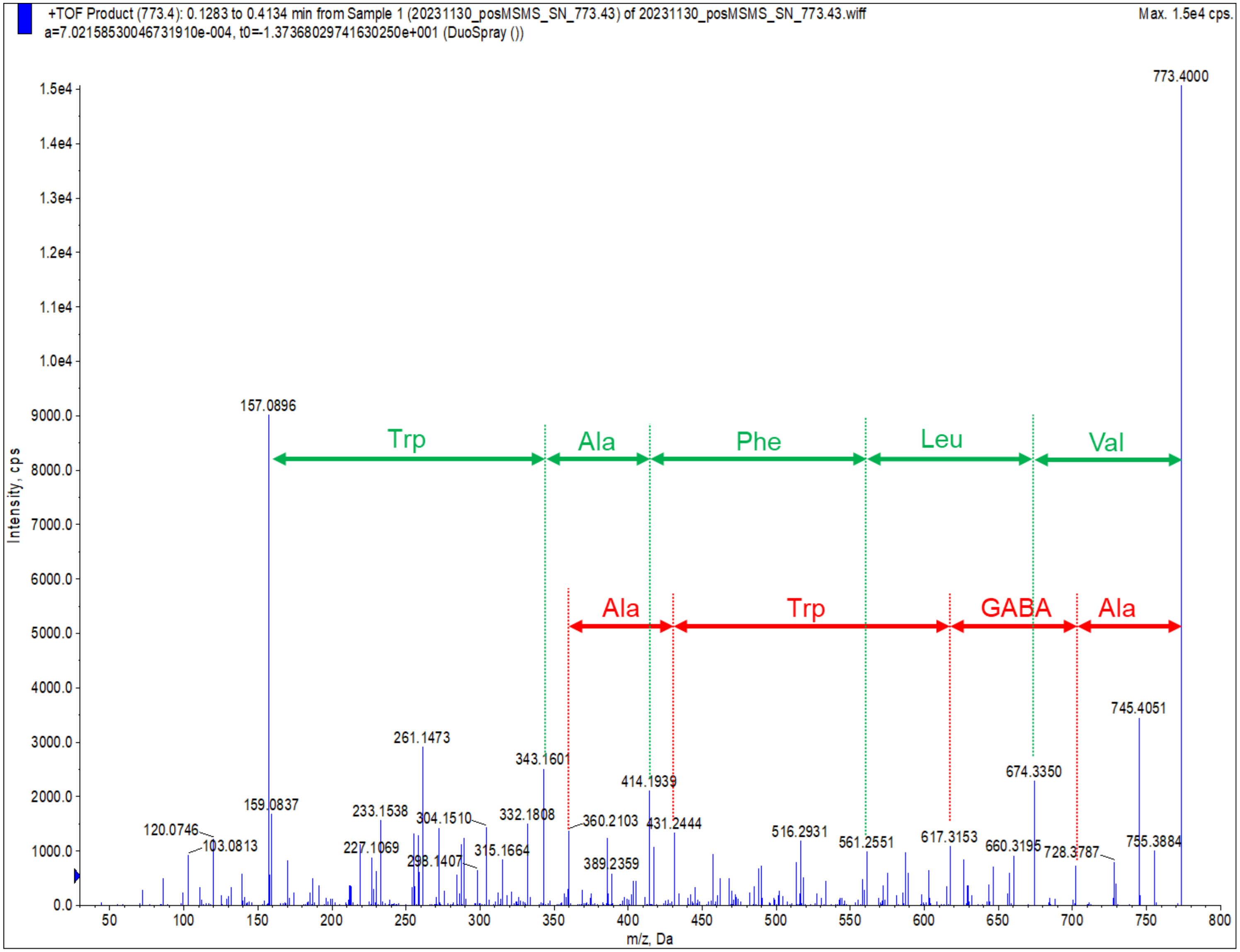

Figure S15: HRESIMS/MS spectra of **1** and fragmentation patterns in positive ionization mode.

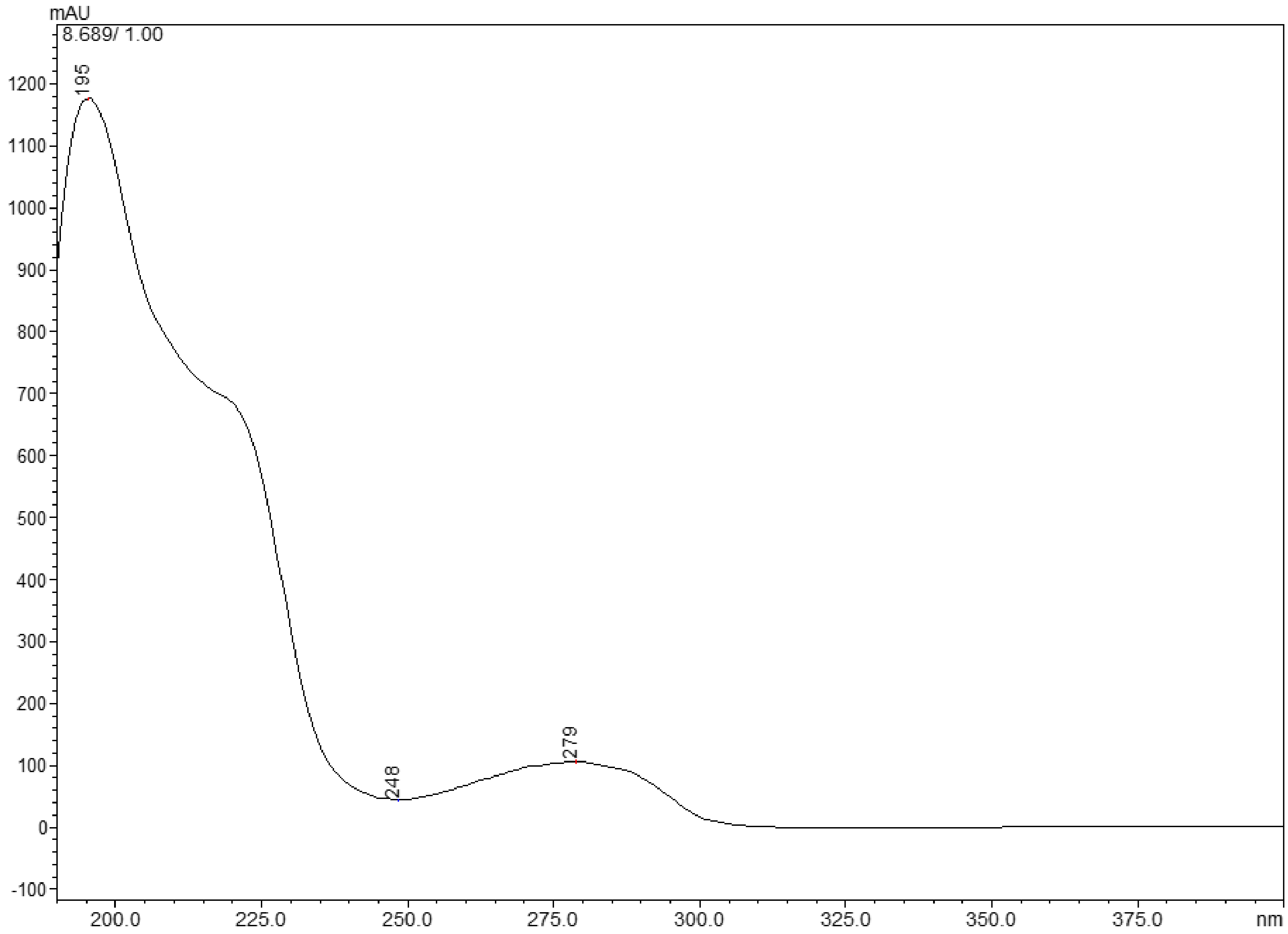

Figure S16: UV spectrum (photodiode array, H_2O:MeCN) of **1**.

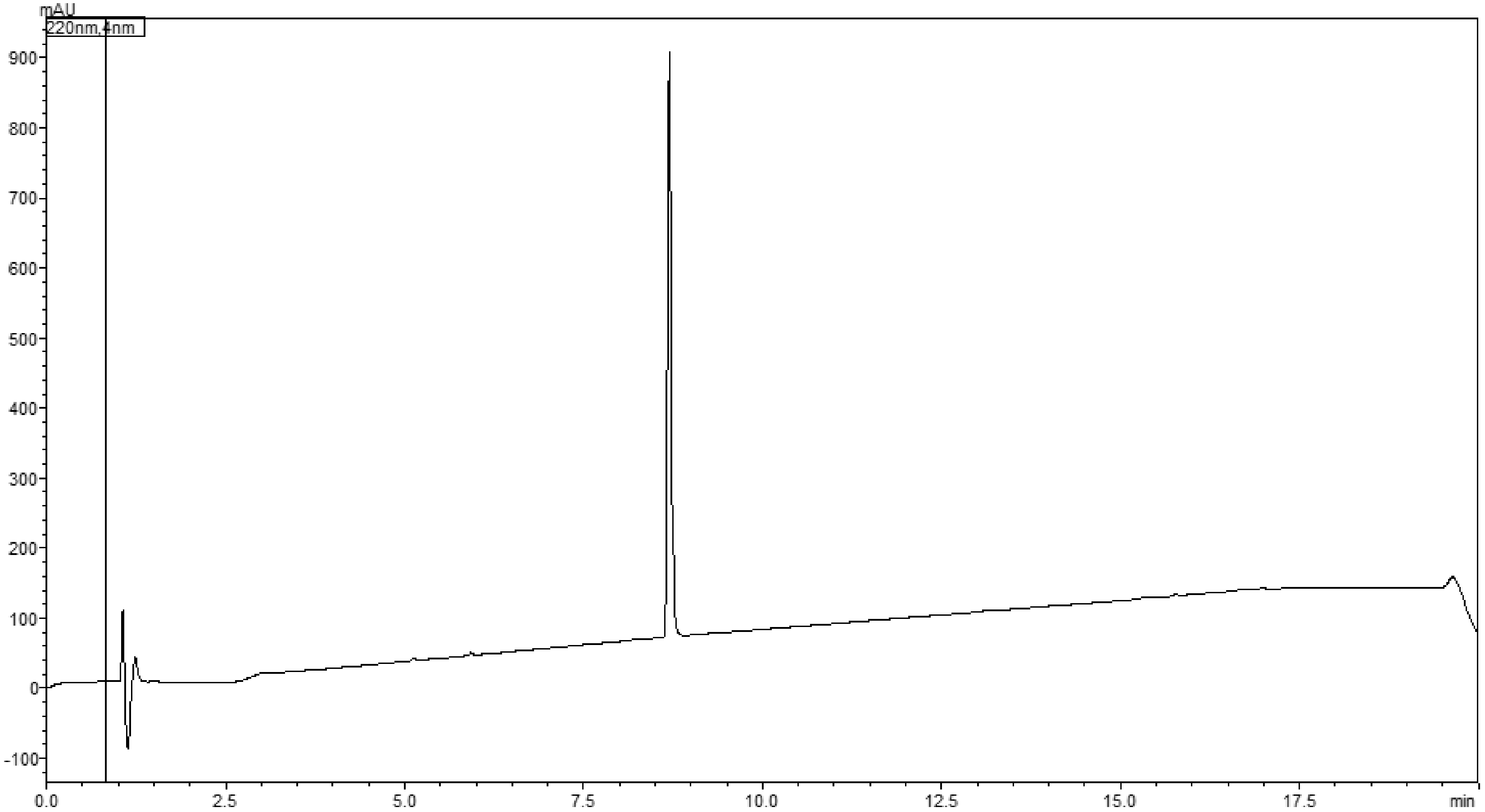

Figure S17: HPLC-PDA chromatogram of unguisin J (**1**).

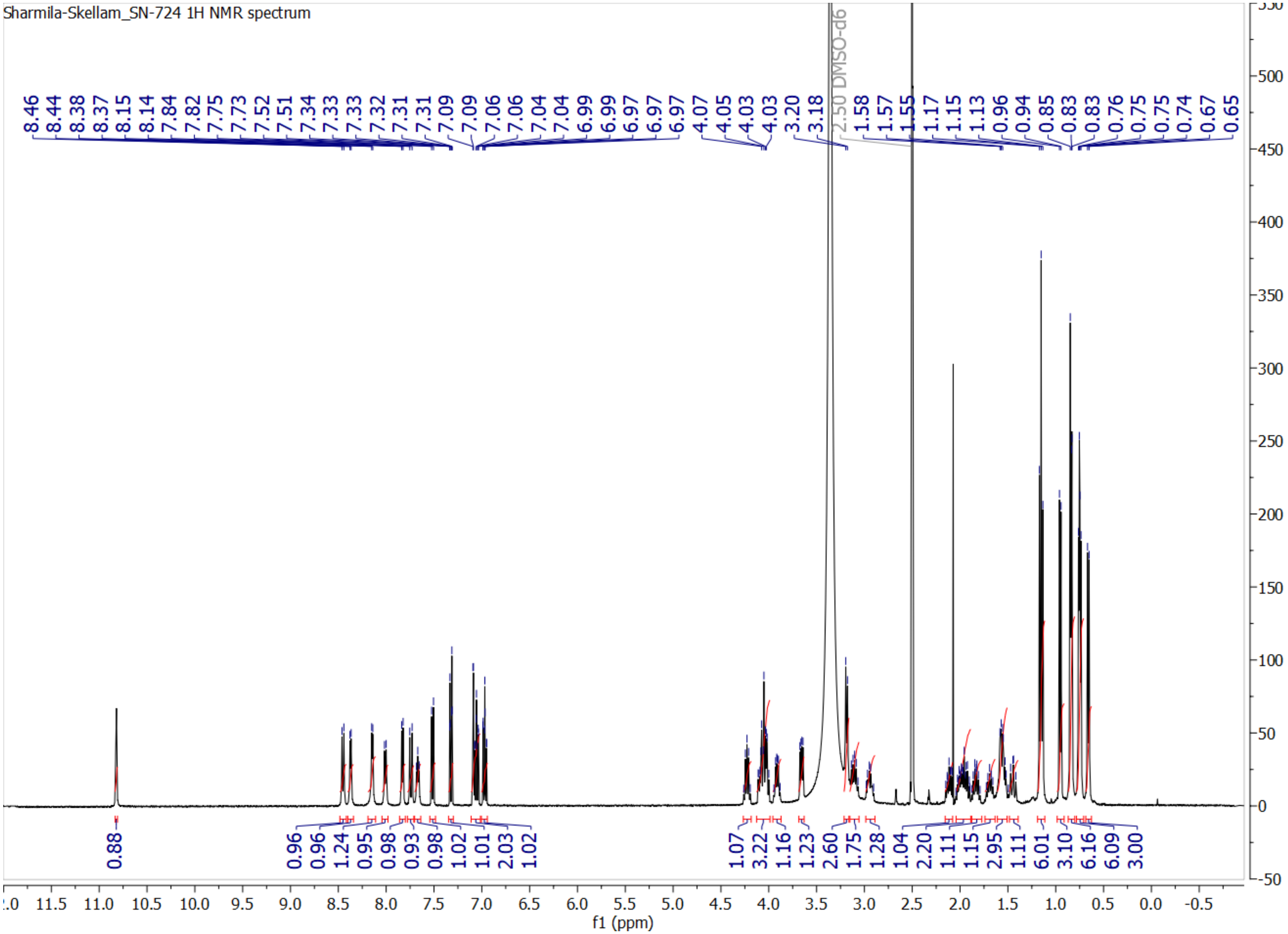

Figure S18: ^{1}H NMR spectrum of **2** (500 MHz, DMSO-d_6).

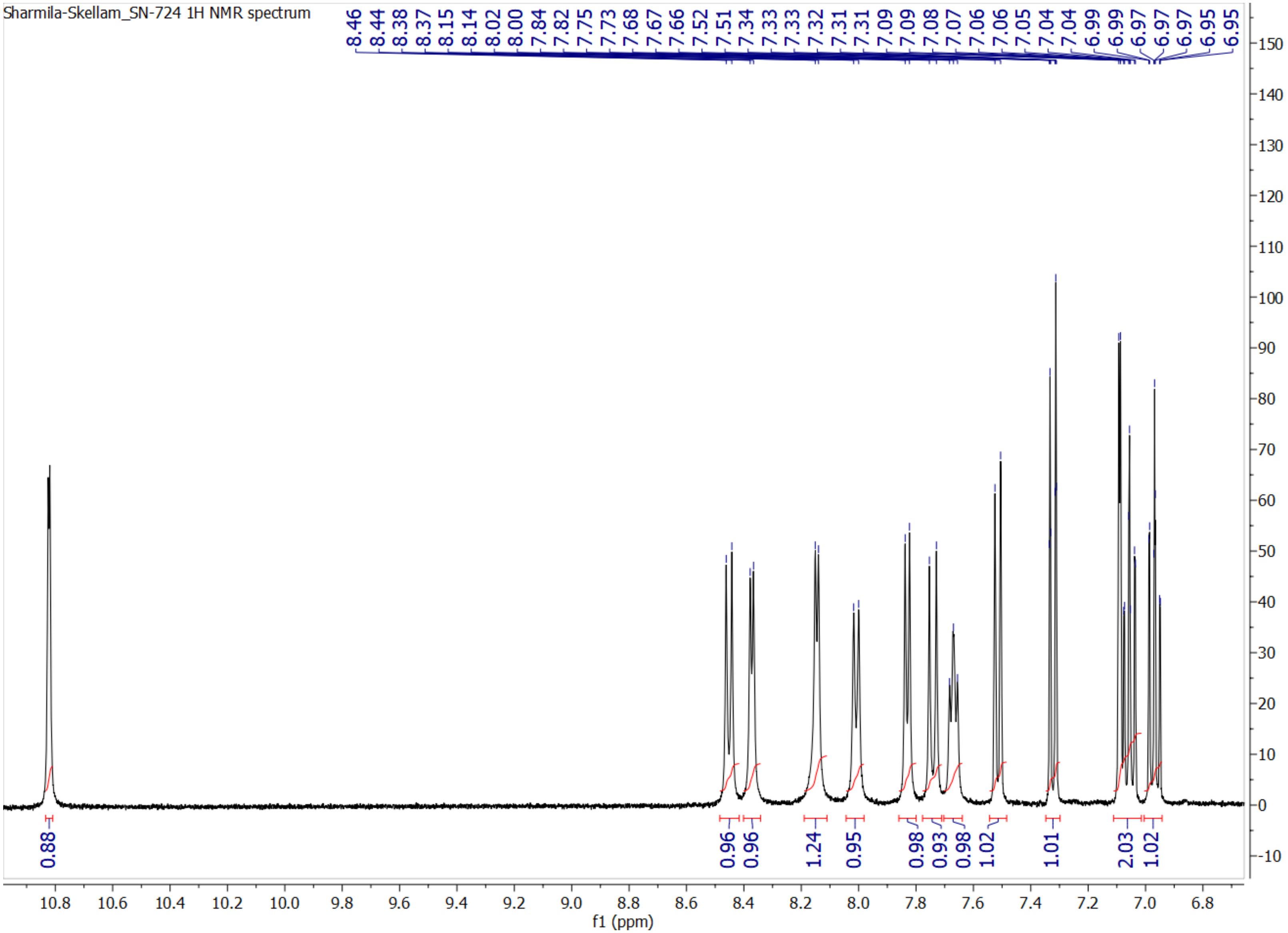

Figure S19: Expansion of the ^{1}H NMR spectrum of **2** (500 MHz, DMSO-d_6).

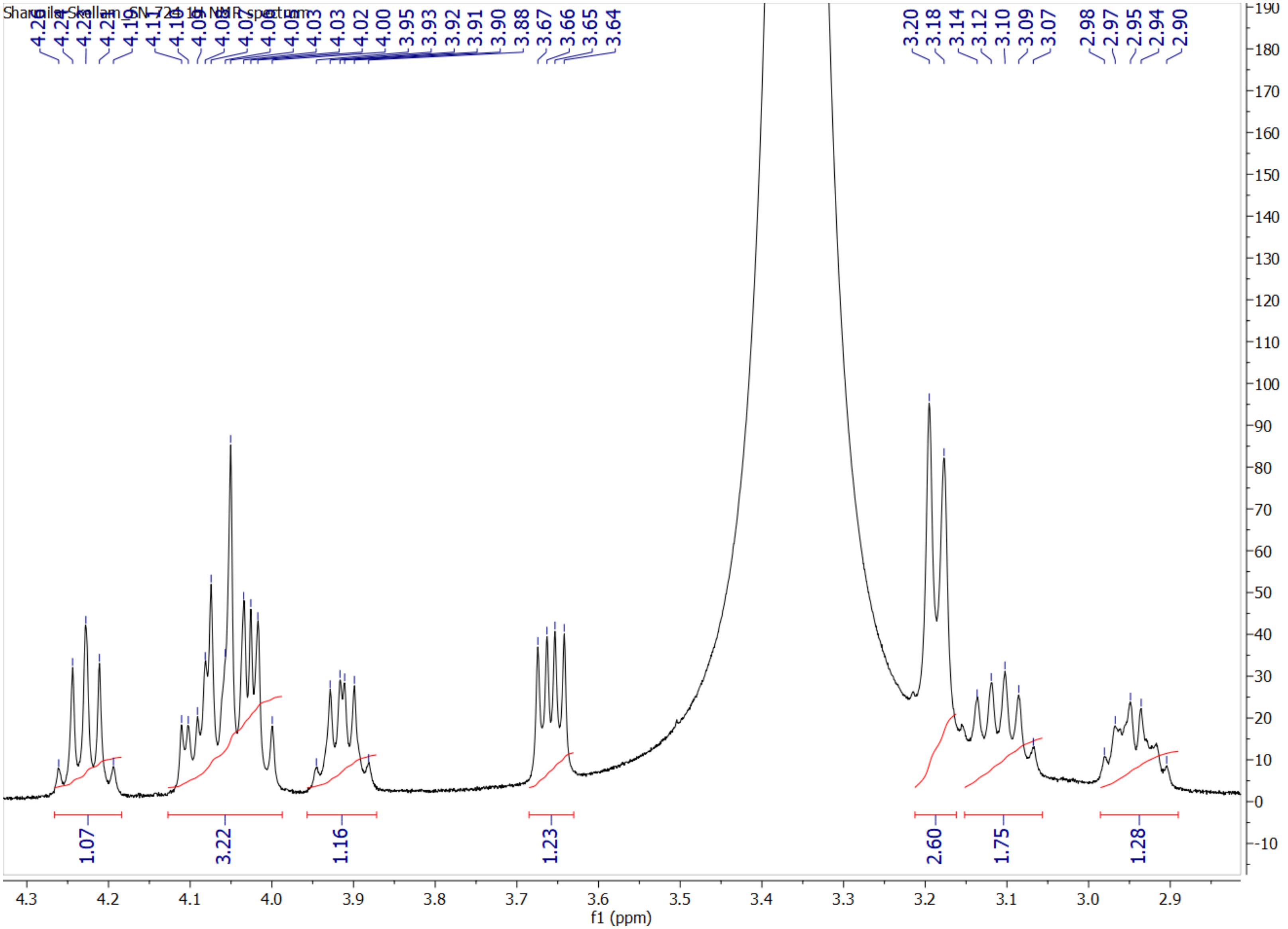

Figure S20: Expansion of the ^{1}H NMR spectrum of **2** (500 MHz, DMSO-d_6).

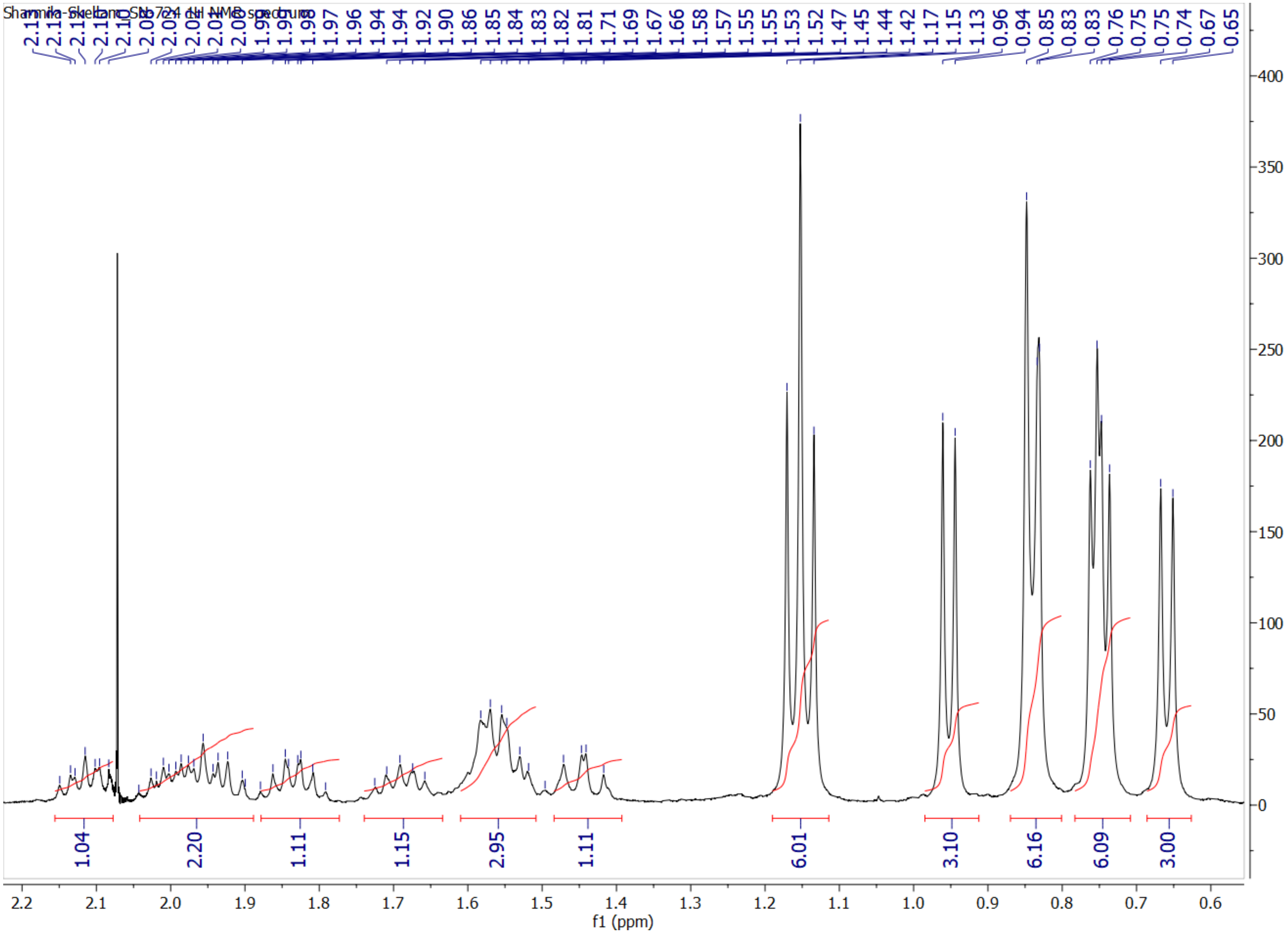

Figure S21: Expansion of the ^{1}H NMR spectrum of **2** (500 MHz, DMSO-d_6).

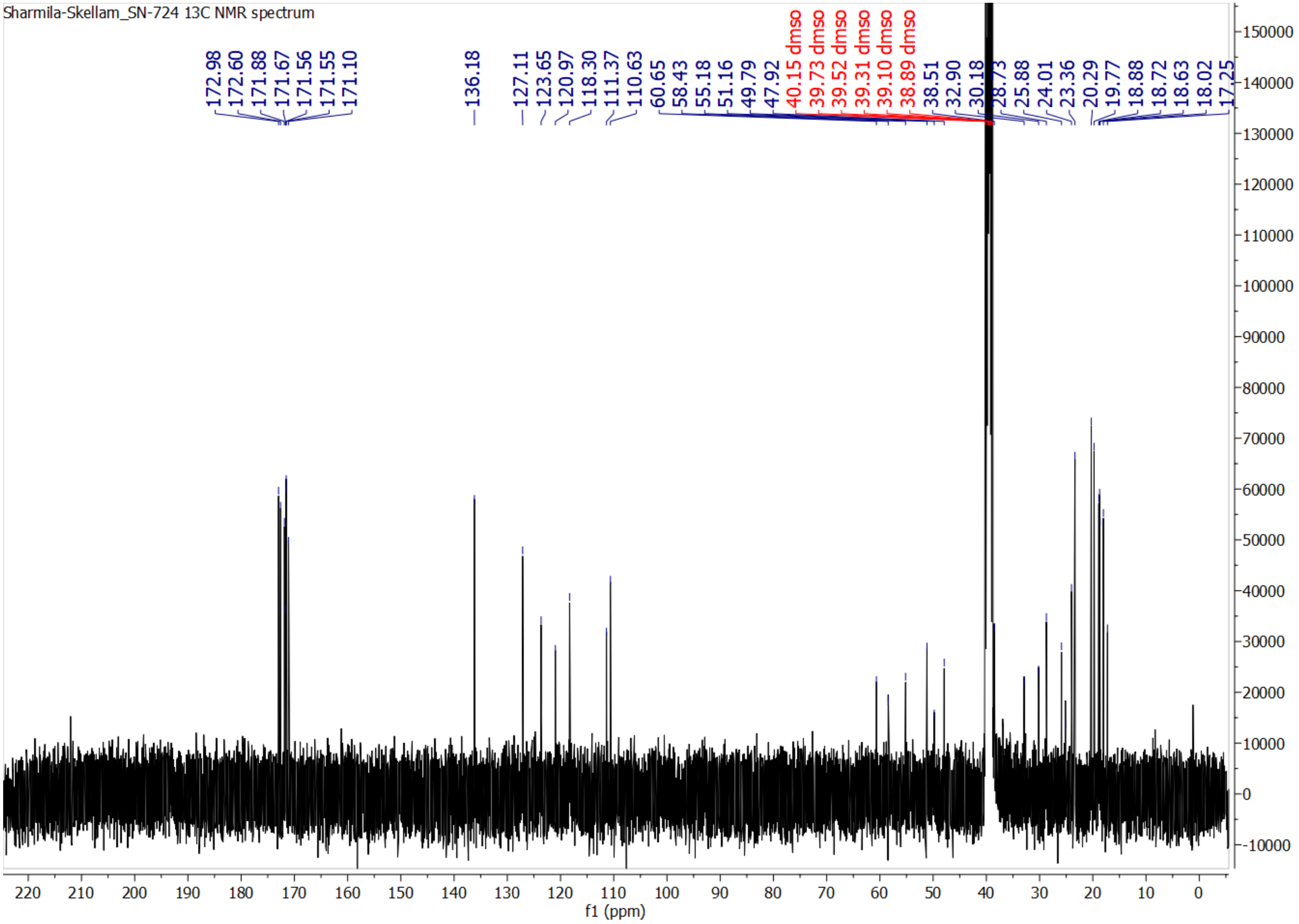

Figure S22: ^{13}C NMR spectrum of **2** (125 MHz, DMSO-d_6).

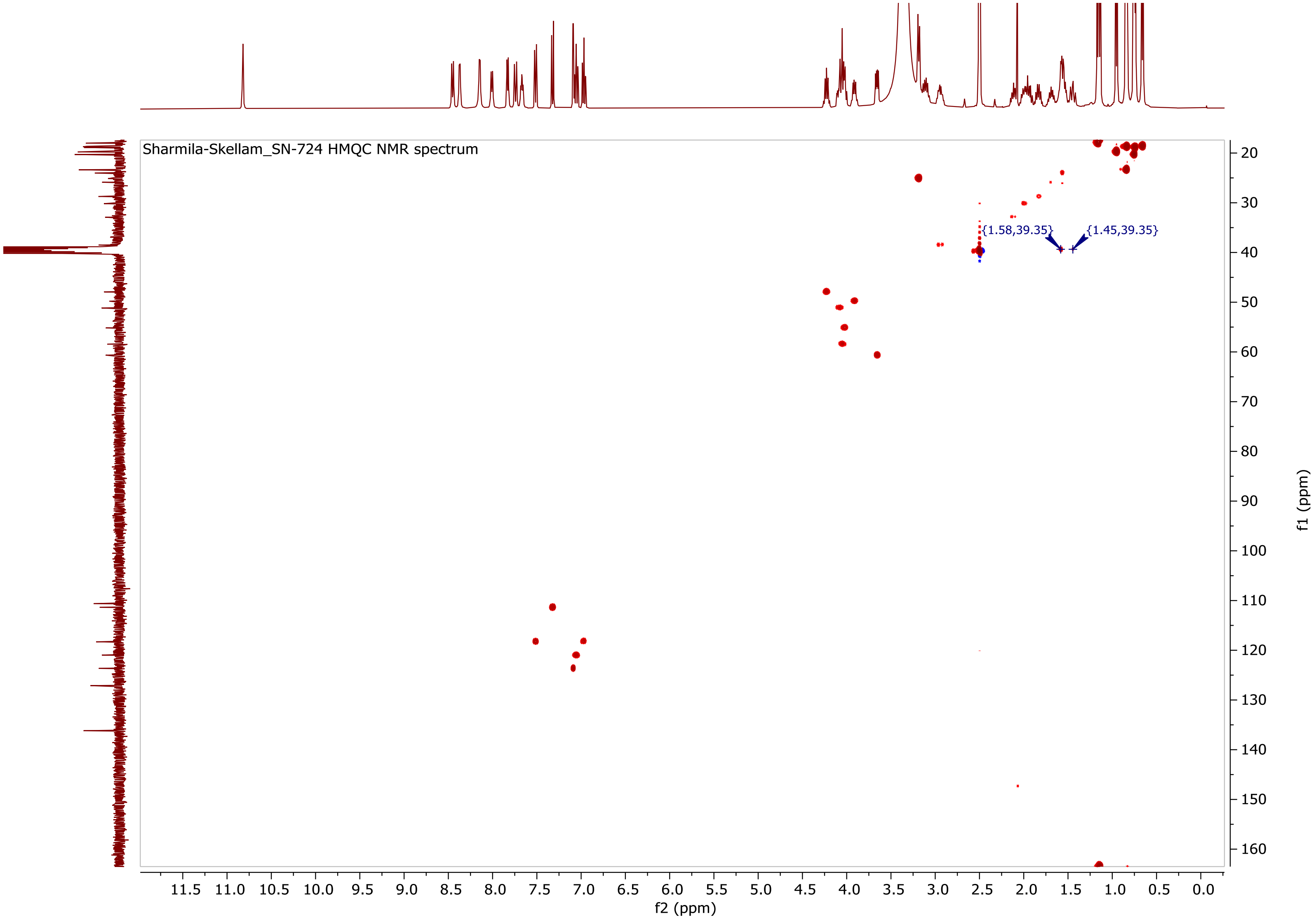

Figure S23: *g*HSQC NMR spectrum of **2** (^{1}H:500 MHz; ^{13}C:125 MHz, DMSO-*d*$_6$).

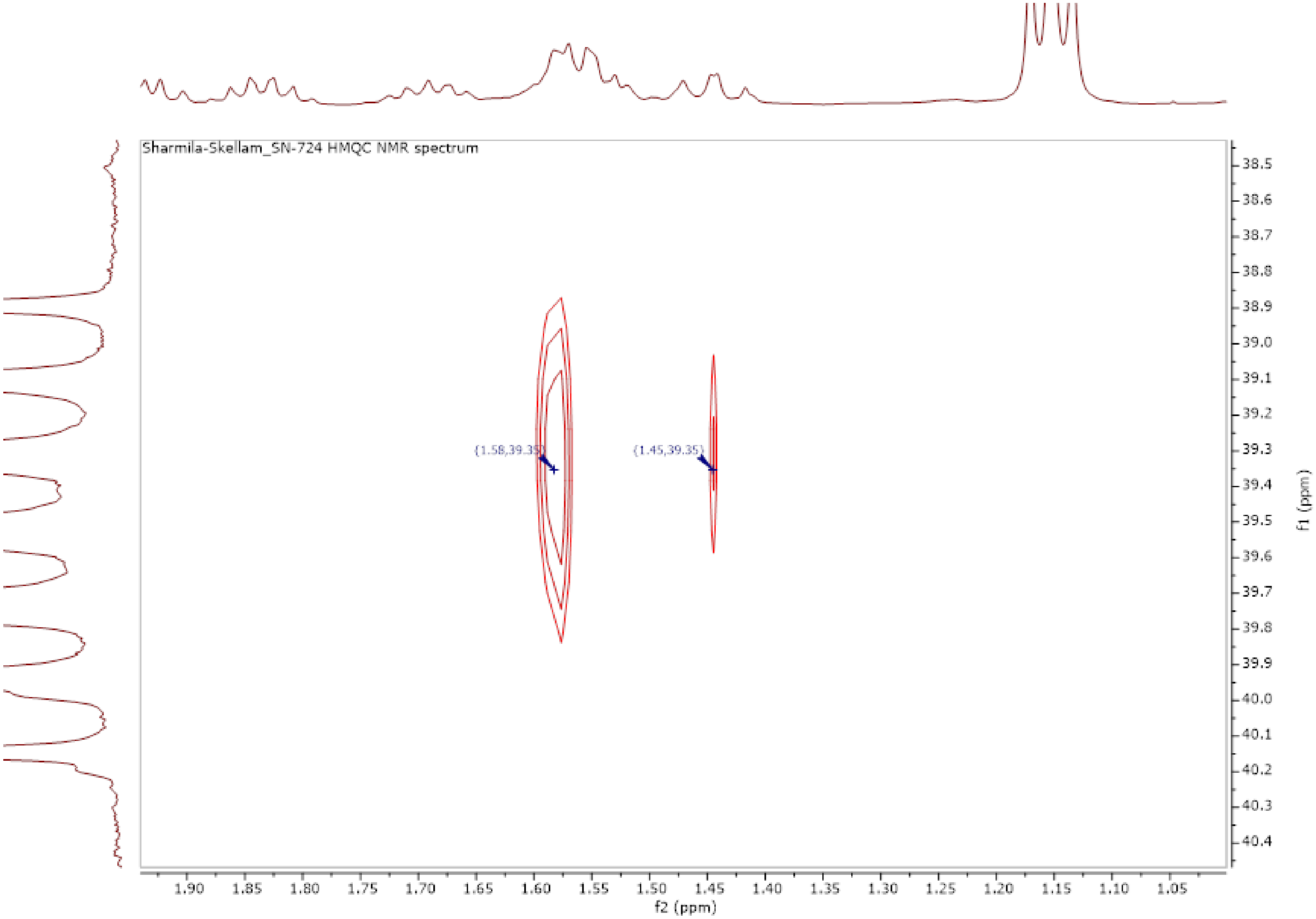

Figure S24: Expansion of the *g*HSQC NMR spectrum of **2** (^{1}H:500 MHz; ^{13}C:125 MHz, DMSO-d_6).

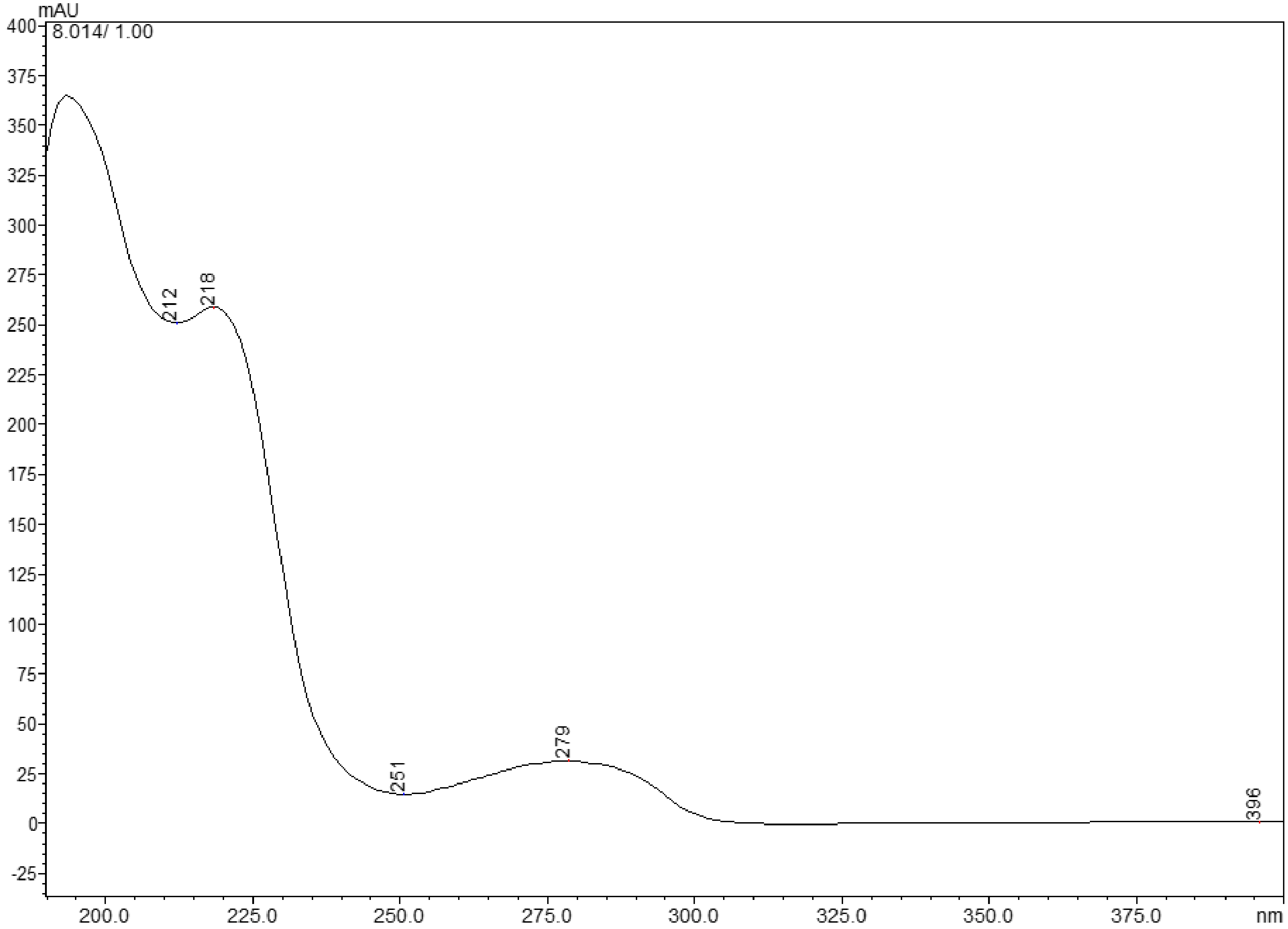

Figure S25: UV spectrum (photodiode array, H_2O:MeCN) of **2**.

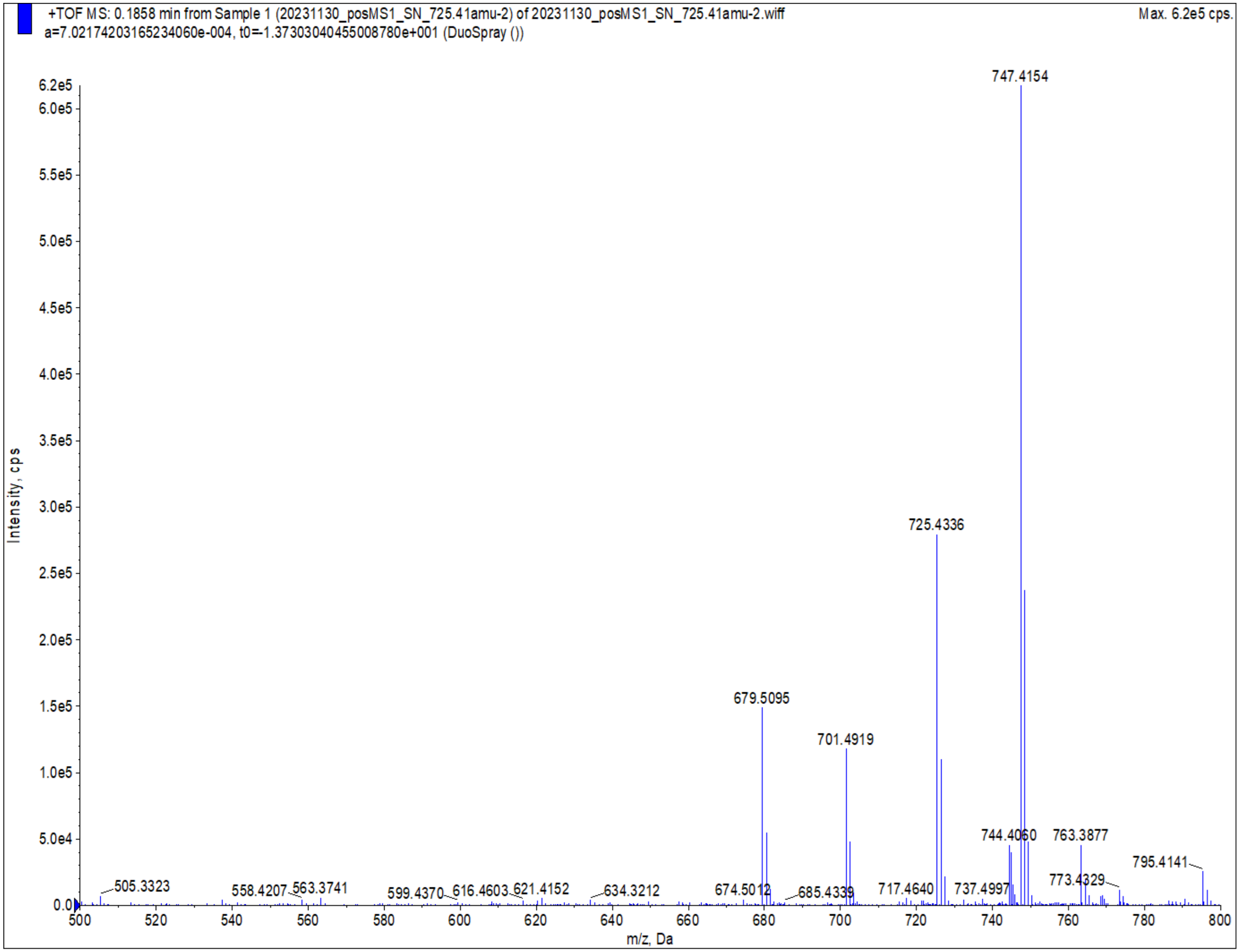

Figure S26: HRESIMS spectrum of **2** in positive ionization mode.

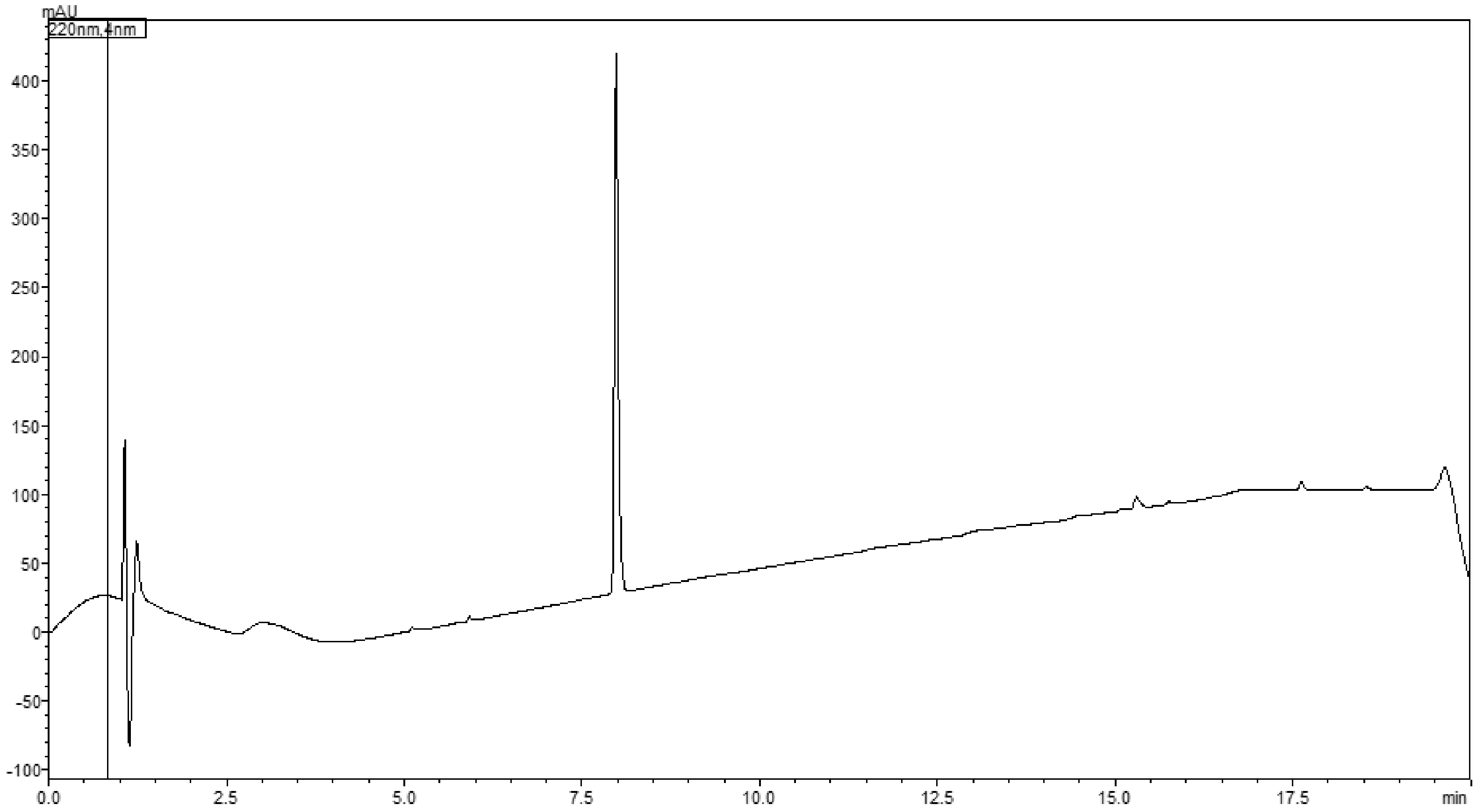

Figure S27: HPLC-PDA chromatogram of unguisin B (**2**).

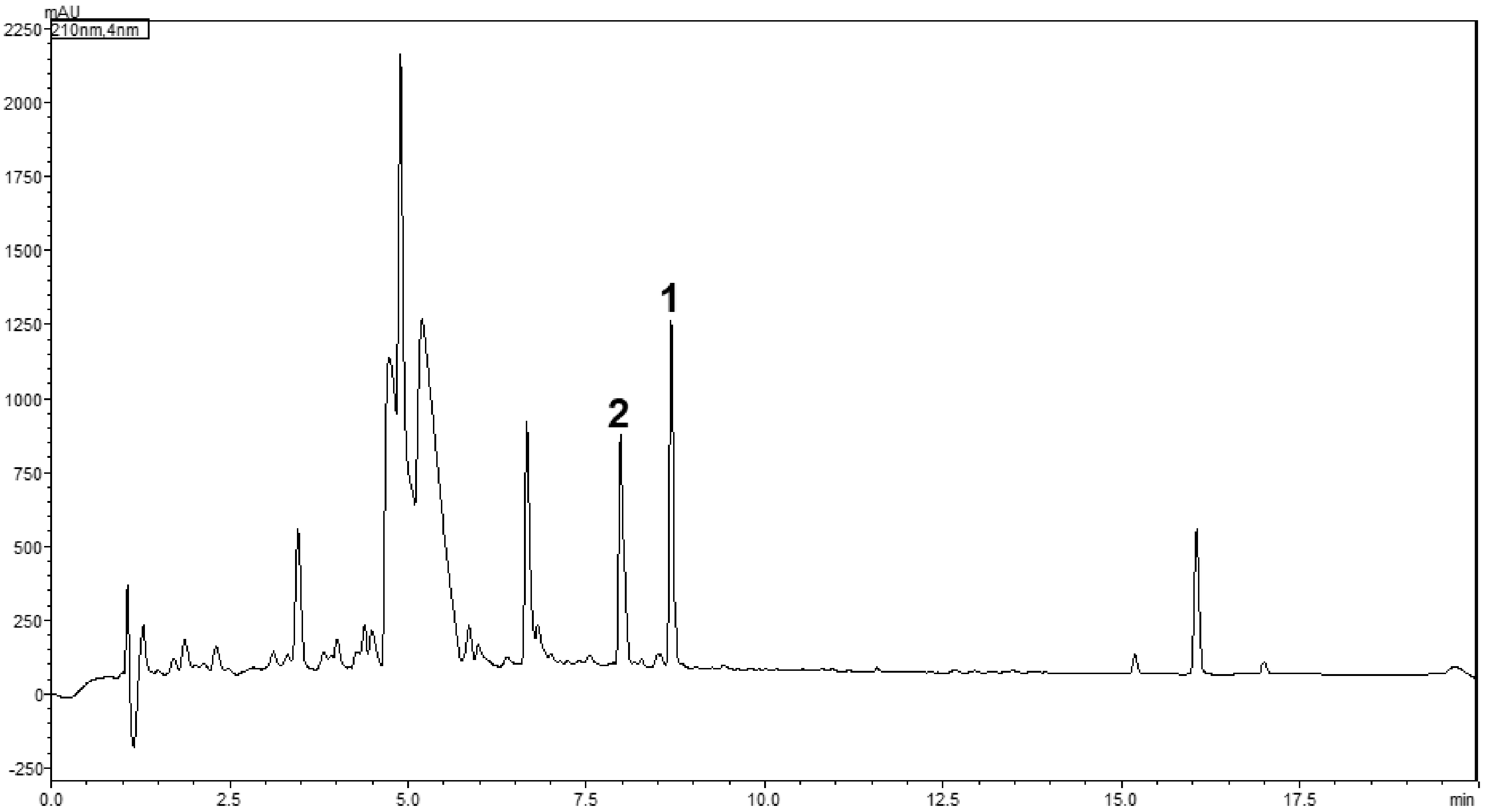

Figure S28: HPLC-PDA chromatogram of soluble-organic extract from rice culture of *A. heteromorphus*. Peak labels represent Unguisin J (**1**) and Unguisin B (**2**).

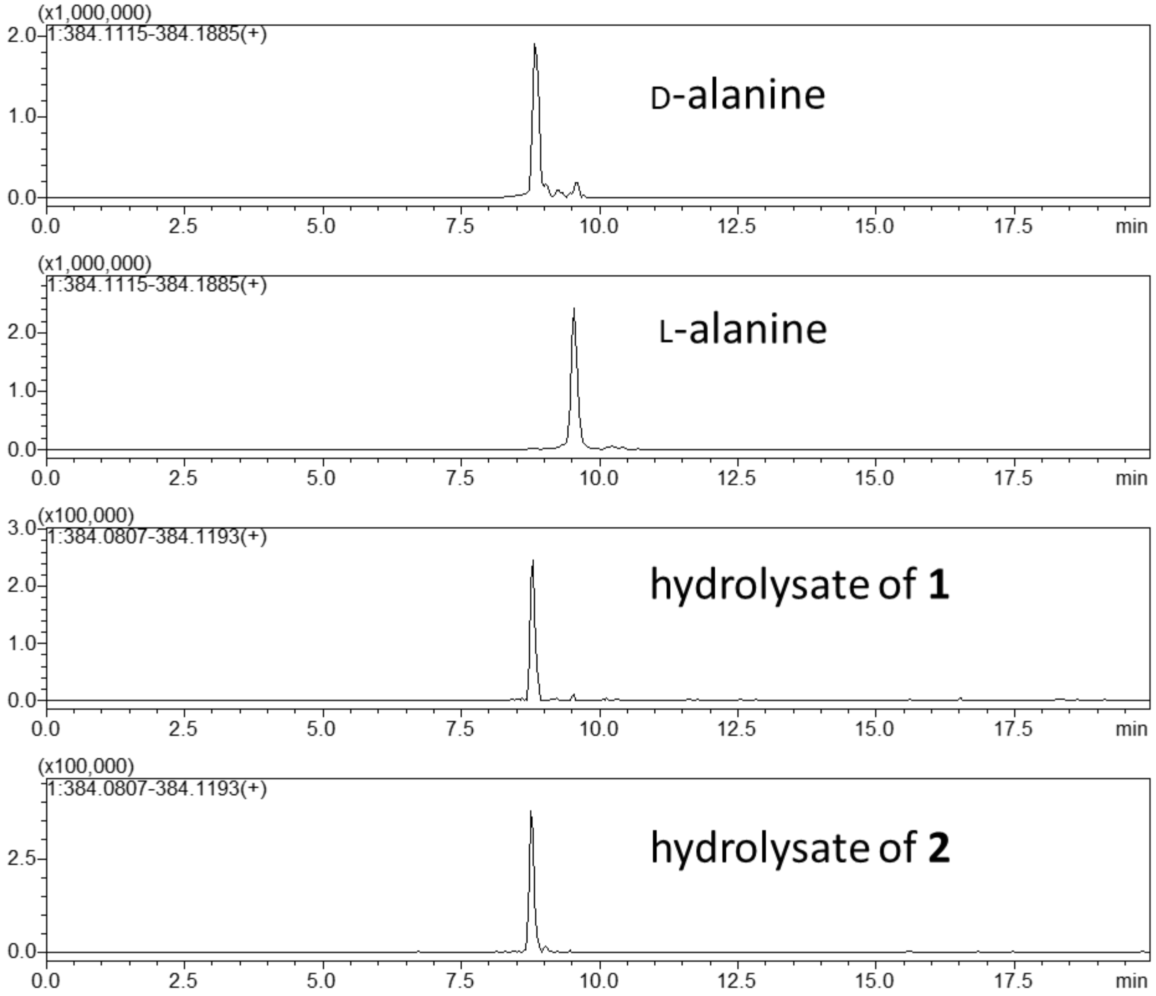

Figure S29: LC-MS analysis of N^{α}-(5-fluoro-2,4-dinitrophenyl)-D-leucinamide derivatives of the hydrolysates of **1** and **2** as well as the alanine amino acid standards.

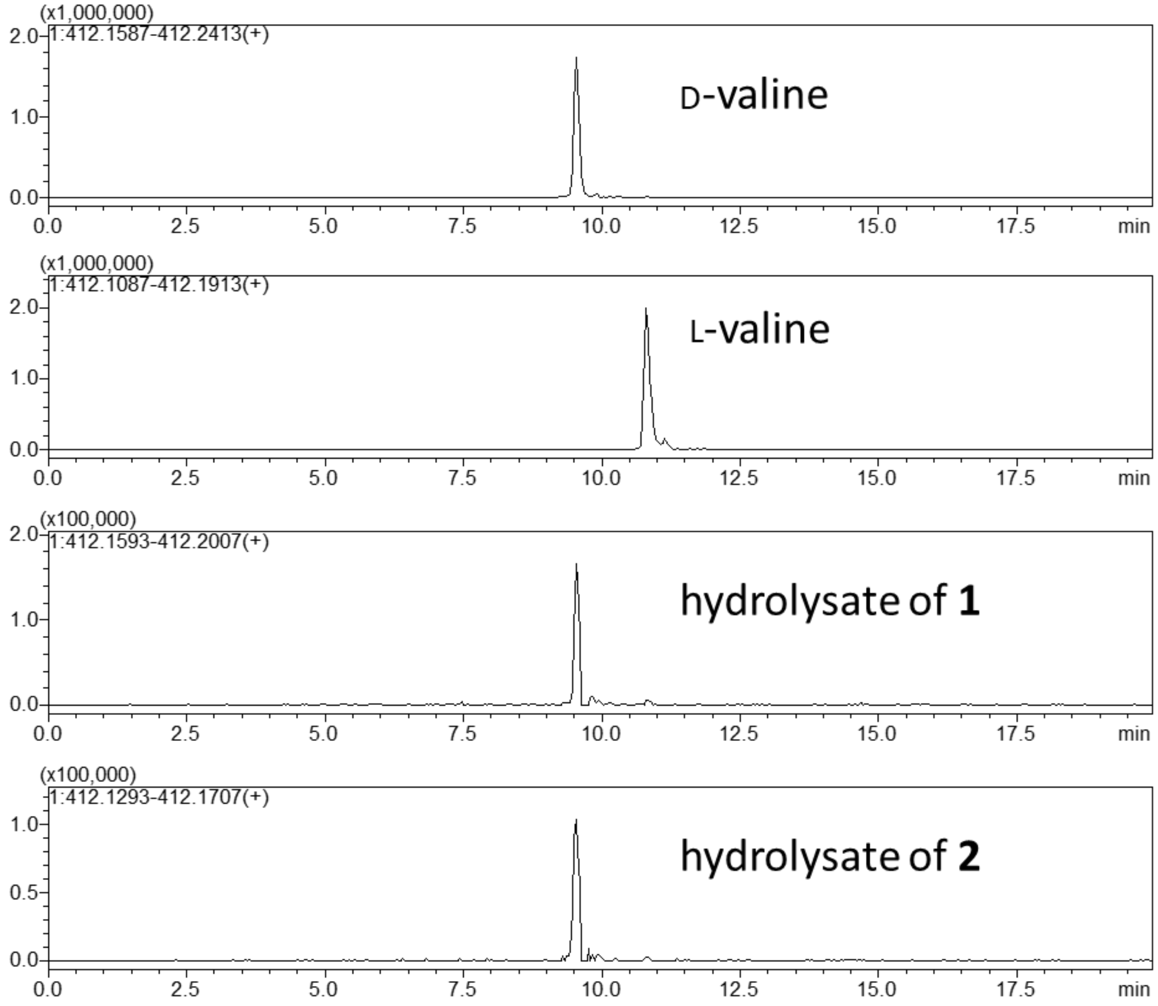

Figure S30: LC-MS analysis of N^{α}-(5-fluoro-2,4-dinitrophenyl)-D-leucinamide derivatives of the hydrolysates of **1** and **2** as well as the valine amino acid standards.

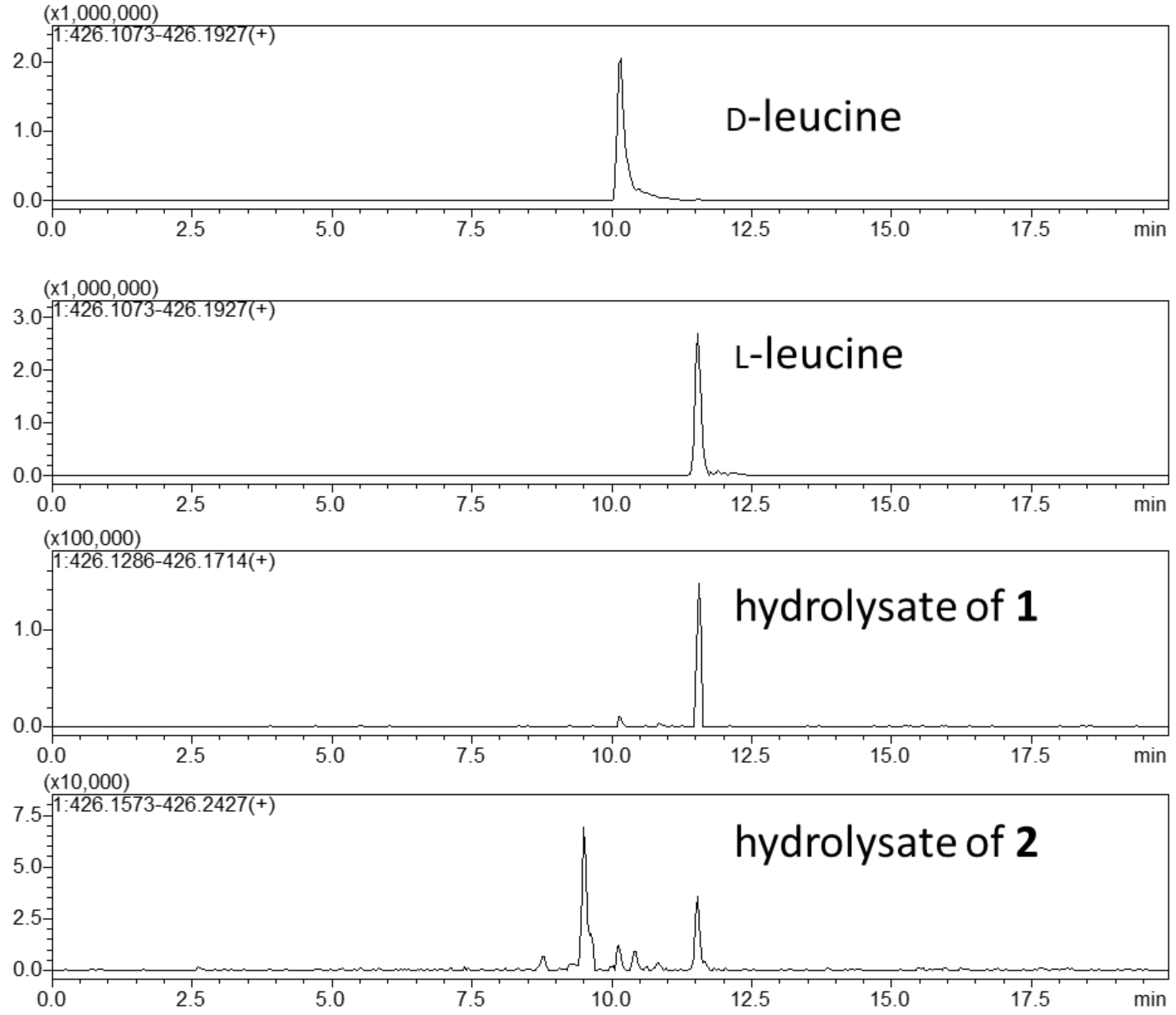

Figure S31: LC-MS analysis of N^{α}-(5-fluoro-2,4-dinitrophenyl)-D-leucinamide derivatives of the hydrolysates of **1** and **2** as well as the leucine amino acid standards.

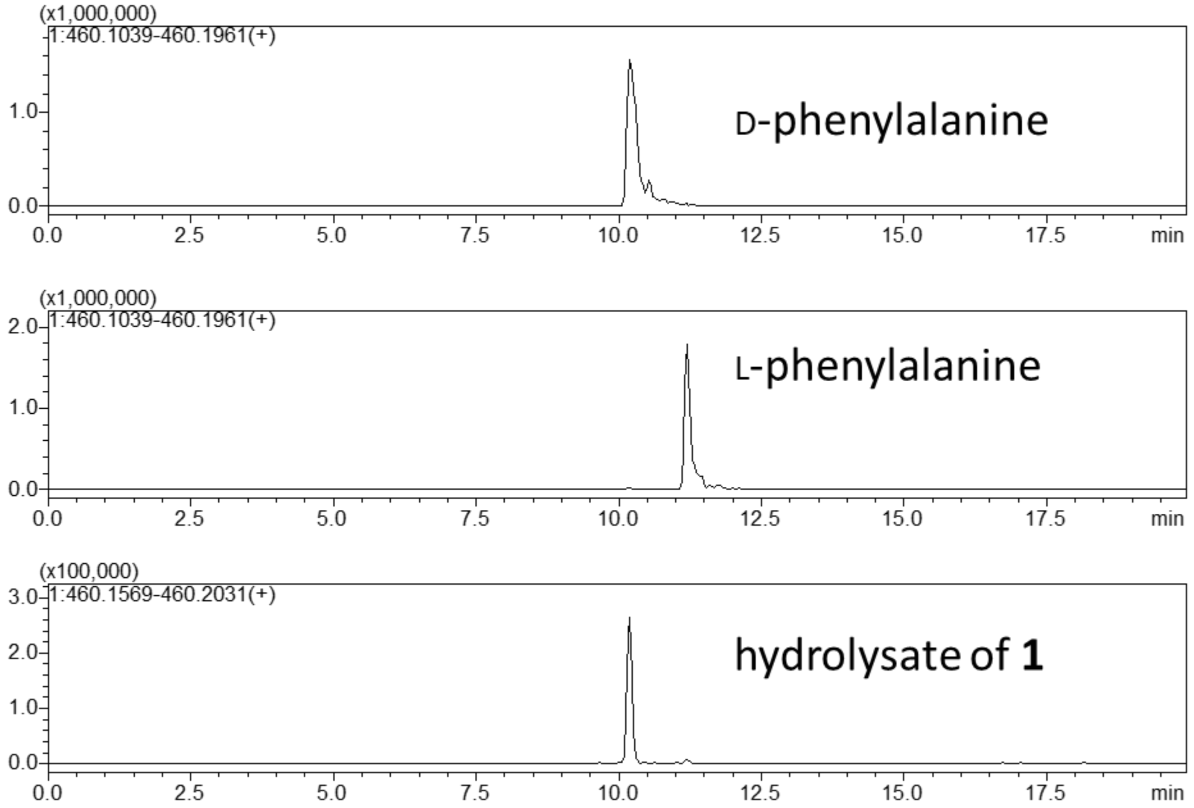

Figure S32: LC-MS analysis of N^{α}-(5-fluoro-2,4-dinitrophenyl)-D-leucinamide derivative of the hydrolysate of **1** as well as the phenylalanine amino acid standards.

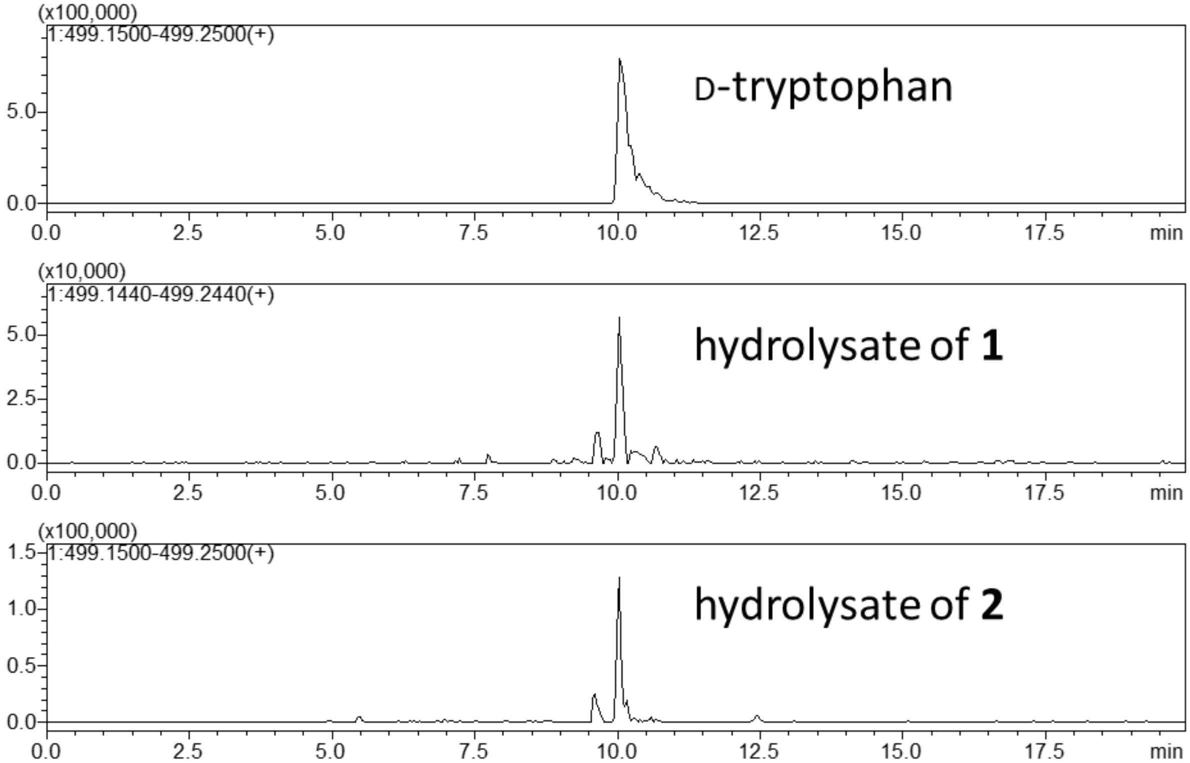

Figure S33: LC-MS analysis of N^{α}-(5-fluoro-2,4-dinitrophenyl)-D-leucinamide derivatives of the hydrolysates of **1** and **2** as well as the D-tryptophan standard.

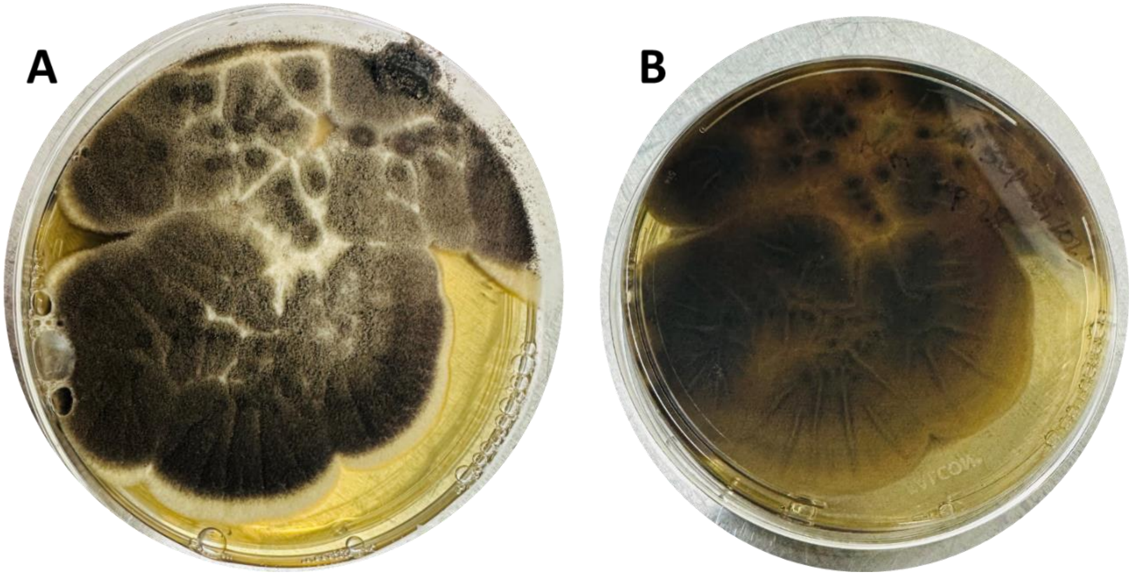

Figure S34: Colonies of *Aspergillus heteromorphous* CBS 117.55 with 7 days of growth at 25 °C. Colonies on DPY agar plate verse (**A**) and obverse (**B**).

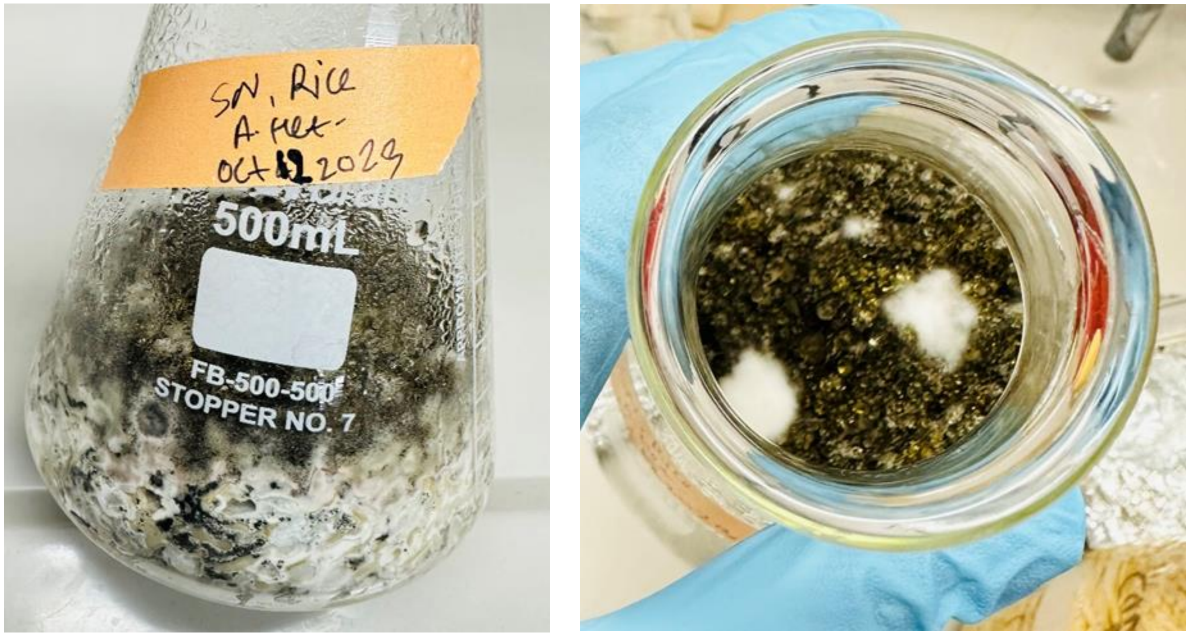

Figure S35: Colonies of *Aspergillus heteromorphous* CBS 117.55 with 21 days of growth at 25 °C on solid rice in Erlenmeyer flasks.

Table S2: ^{1}H and ^{13}C NMR data for **2** (^{1}H: 500MHz, ^{13}C:125MHz; DMSO-d_6).

			**2**		**Unguisin B (Malmstron, 1999)**
**residue/position**		δ_C	δ_H **(mult., *J* in Hz)**	δ_C	δ_H **(mult., *J* in Hz)**
Alanine	NH	-	8.37 (d, 4.9)	-	8.36 (d, 4.6)
	Cα	49.8	3.91 (m)	49.8	3.92 (m)
	Cβ	17.3	1.14 (d, 7.1)	17.3	1.16
Alanine	NH	-	7.83 (d, 6.2)	-	7.82 (d, 6.1)
	Cα	47.9	4.22 m	48.0	4.23 (m)
	Cβ	18.0	1.16 (d, 7.1)	18.1	1.16
Valine	NH	-	8.14 (d, 4.5)	-	8.13 (d, 4.4)
	Cα	60.6	3.66 (dd, 8.4, 4.4)	60.6	3.67 (dd, 4.6, 8.2)
	Cβ	28.7	1.83 (m)	28.8	1.82 (m)
	Cγ	19.8	0.95 (d, 6.7)	19.7	0.95 (d, 6.6)
	Cγ	18.7	0.84 (d, 6.4)	18.8	0.84 (d, 6.4)
Valine	NH	-	7.74 (d, 9.8)	-	7.73 (d, 9.7)
	Cα	58.4	4.05 (m)	58.5	4.07 (m)
	Cβ	30.2	2.00 (m)	30.2	2.00 (m)
	Cγ	18.6	0.66 (d, 6.6)	18.6	0.66 (d, 6.6)
	Cγ	18.9	0.74 (d, 6.6)	18.9	0.74
GABA	NH	-	7.67 (t, 5.5)	-	7.65 (t, 5.3)
	Cα	38.5	3.11 (m), 2.94 (m)	38.5	3.11 (m), 2.95 (m)
	Cβ	25.9	1.69 (m), 1.55 (m)	25.9	1.65 (m), 1.58 (m)
	Cγ	32.9	2.11 (m), 1.95 (m)	32.9	2.11 (m), 1.94 (m)
Tryptophan	NH	-	8.0 (d, 7.1)	-	7.98 (d, 6.8)
	Cα	55.2	4.02 (m)	55.2	4.05 (m)
	Cβ	25.2	3.18 (d, 7.2)	25.2	3.20 (d, 8.5)
	NH	-	10.82 (d, 2.4)	-	10.81 (d, 2.0)
	C-2	123.7	7.08 (d, 2.3)	123.6	7.11 (d, 2.2)
	C-3	110.6	-	110.6	-
	C3a	127.1	-	127.1	-
	C-4	118.3	7.51 (d, 7.9)	118.3	7.54 (d, 7.9)
	C-5	121.0	7.06 (ddd, 7.9, 7.1, 1.1)	121.0	7.08 (t, 6.9)
	C-6	118.3	6.97 (ddd, 8.1, 7.1, 1.1)	118.3	6.97 (t, 7.2)
	C-7	111.4	7.32 (dt, 8.1, 0.8)	111.4	7.35 (d 8.1)
	C-7a	136.2	-	136.2	-
Leucine	NH	-	8.45 (d, 7.9)	-	8.43 (d, 7.7)
	Cα	51.2	4.07 (m)	51.2	4.09 (m)
	Cβ	39.4[a]	1.58 (m), 1.45 (m)	38.7	1.58 (m)
	Cγ	24.0	1.57 (m)	24.1	1.58 (m)
	Cδ	23.4	0.84 (d, 6.4)	23.4	0.84 (d, 6.4)
	Cδ	20.3	0.75 (d, 6.0)	20.4	0.74

[a]Confirmed by HSQC. Carbons signals at δ_C 171.1, 171.6, 171.6, 171.7, 171.9, 172.6 and 173.0 ppm.

Table S3: Summary of additional NRPS identified in the genome of *A. heteromorphus* CBS 117.55. Abbreviations as in main text; R = reductase.

fungiSMASH contig ID	NCBI GeneID	Putative NRPS size (aa)	Putative domain arrangement
2.2	37069286	4028	C_T-A-PCP-C-A-MT-PCP-C-A-PCP-TE
35.2	37070309	5593	A-PCP-C-A-E-PCP-C-A-E-PCP-A-C-PCP-C-PCP
57.3	37069398	1436	C-A-PCP-R
119.1	37067680	5115	A-PCP-A-PCP-C-A-PCP-C-A-PCP-C-A-PCP
139.1	37063074	2302	A-PCP-C-A-PCP-R
143.1	37069176	2452	A-PCP-C-A-PCP-C_T
150.1	37069727	9562	A-PCP-C-A-PCP-E-C-A-PCP-C-A-PCP-E-C-A-PCP-E-C-A-PCP-E-C-A-PCP-C_T
161.1	37069763	2775	A-PCP-C-A-PCP-E (truncated; close to scaffold edge)
172.1	37061517	671	A-PCP (truncated; close to scaffold edge)
172.2	37067500	4765	A-PCP-C-A-PCP-C-A-PCP-E-A-PCP-C (truncated; close to scaffold edge)
172.5	37069805	1931	A-PCP-C-A-PCP (truncated; close to scaffold edge)
